# Transcriptome analysis of air-breathing land slug, *Incilaria* fruhstorferi reveals functional insights into growth, immunity, and reproduction

**DOI:** 10.1186/s12864-019-5526-3

**Published:** 2019-02-26

**Authors:** Bharat Bhusan Patnaik, Jong Min Chung, Hee Ju Hwang, Min Kyu Sang, Jie Eun Park, Hye Rin Min, Hang Chul Cho, Neha Dewangan, Snigdha Baliarsingh, Se Won Kang, So Young Park, Yong Hun Jo, Hong Seog Park, Wan Jong Kim, Yeon Soo Han, Jun Sang Lee, Yong Seok Lee

**Affiliations:** 1 Department of Life Science and Biotechnology, College of Natural Sciences, Soonchunhyang University, 22 Soonchunhyangro, Shinchang-myeon, Asan, Chungchungnam-do 31538 South Korea; 2 School of Biotech Sciences, Trident Academy of Creative Technology (TACT), F2-B, Chandaka Industrial Estate, Chandrasekharpur, Bhubaneswar, Odisha 751024 India; 3 Biological Resource Center, Korea Research Institute of Bioscience and Biotechnology (KRIBB), 181, Ipsin-gil, Jungeup-si, Jeollabuk-do 56212 South Korea; 4 Nakdonggang National Institute of Biological Resources, Biodiversity Conservation and Change Research Division, 137, Donam-2-gil, Sangju-si, Gyeongsangbuk-do 37242 South Korea; 5 College of Agriculture and Life Science, Chonnam National University, 77 Yongbong-ro, Buk-gu, Gwangju, 61186 South Korea; 6 Research Institute, GnC BIO Co., LTD, 621-6 Banseok-dong, Yuseong-gu, Daejeon, 34069 Republic of Korea; 7 Institute of Basic Science, Soonchunhyang University, 22 Soonchunhyangro, Shinchang-myeon, Asan, Chungchungnam-do, 31538 South Korea

**Keywords:** *Incilaria fruhstorferi*, Transciptome, de novo analysis, Immunity, Sex-linked genes, Simple sequence repeats, Tollip, Peptidoglycan recognition protein

## Abstract

**Background:**

*Incilaria (= Meghimatium) fruhstorferi* is an air-breathing land slug found in restricted habitats of Japan, Taiwan and selected provinces of South Korea (Jeju, Chuncheon, Busan, and Deokjeokdo). The species is on a decline due to depletion of forest cover, predation by natural enemies, and collection. To facilitate the conservation of the species, it is important to decide on a number of traits related to growth, immunity and reproduction addressing fitness advantage of the species.

**Results:**

The visceral mass transcriptome of *I. fruhstorferi* was enabled using the Illumina HiSeq 4000 sequencing platform. According to BUSCO (Benchmarking Universal Single-Copy Orthologs) method, the transcriptome was considered complete with 91.8% of ortholog genes present (Single: 70.7%; Duplicated: 21.1%). A total of 96.79% of the raw read sequences were processed as clean reads. TransDecoder identified 197,271 contigs that contained candidate-coding regions. Of a total of 50,230 unigenes, 34,470 (68.62% of the total unigenes) annotated to homologous proteins in the Protostome database (PANM-DB). The GO term and KEGG pathway analysis indicated genes involved in metabolism, phosphatidylinositol signalling system, aminobenzoate degradation, and T-cell receptor signalling pathway. Many genes associated with molluscan innate immunity were categorized under pathogen recognition receptor, TLR signalling pathway, MyD88 dependent pathway, endogenous ligands, immune effectors, antimicrobial peptides, apoptosis, and adaptation-related. The reproduction-associated unigenes showed homology to protein fem-1, spermatogenesis-associated protein, sperm associated antigen, and testis expressed sequences, among others. In addition, we identified key growth-related genes categorized under somatotrophic axis, muscle growth, chitinases and collagens. A total of 4822 Simple Sequence Repeats (SSRs) were also identified from the unigene sequences of *I. fruhstorferi*.

**Conclusions:**

This is the first available genomic information for non-model land slug, *I. fruhstorferi* focusing on genes related to growth, immunity, and reproduction, with additional focus on microsatellites and repeating elements. The transcriptome provides access to greater number of traits of unknown relevance in the species that could be exploited for in-depth analyses of evolutionary plasticity and making informed choices during conservation planning. This would be appropriate for understanding the dynamics of the species on a priority basis considering the ecological, health, and social benefits.

**Electronic supplementary material:**

The online version of this article (10.1186/s12864-019-5526-3) contains supplementary material, which is available to authorized users.

## Background

Gastropod slugs are known to inhabit dynamic ecosystems with spectacular local abundances. Most of the slug species were able to expand their habitat requirements over in mountains. But, lately the distributional range of the species has spread to lowlands due to crop irrigation and watering of gardens. *Incilaria fruhstorferi* (NCBI txid 414,506; *syn. Meghimatium fruhstorferi* or *Philomycus fruhstorferi* Collinge, 1901) is a medium to large air-breathing slug belonging to superfamily Arionidae and family Philomycidae [1]. The members of this family including the *Incilaria* species show restricted distribution. *I. fruhstorferi* species has been recorded from Japan, Taiwan, and South Korea (Chuncheon, Busan, Deokjeokdo provinces and Jeju Island) [2].

Considering the local distributions of *Incilaria* species and for its sustainable protection in the wild, it is imperative to implement informed conservation planning practices. This would help to understand habitat-level requirements as well as the potential of acclimatization and adaptation of the species to changing environmental conditions. In this regard, the genomic information of the species would help to screen the relevant phenotypes influencing the distributional range of the species. Currently, the only available genomic information for *I. fruhstorferi* in the National Canter for Biotechnology Information (NCBI) is the cytochrome oxidase subunit I (COI) gene and the C-type lectins viz. Incilarin A, B and C [3]. Although Incilarin could play a putative role in host defense through the recognition of molecular patterns in microbial cell surfaces, a detailed functional characterization is lacking. Further, the lack of genomic information is an impediment towards understanding the ecological adaptation strategies of *Incilaria* sp. in the wild with reference to growth, immunity, and reproduction. Hence, cataloguing the genomic resources would guide successful establishment of the species in the wild.

The advent of effective and cost-efficient next-generation sequencing approaches have led to an increased information on non-model species at the genomic and transcriptomic levels. Conservation biologists and ecologists have found appropriate applications and an added incentive for protecting declining biodiversity in the wake of global climate change. Transcriptomics approaches have identified the regulatory genes with major impacts on development, immunity, and reproduction in many molluscan species including *Aplysia californica* [4], *Crassostrea virginica* [5]*, Biomphalaria glabrata* [6], *Radix auricularia* [7], and the Manila clam, *Ruditapes philippinarum* [8]. Further, completed mitochondrial genomes of gastropods, bivalves, and cephalopods have been used to understand the phylogeny of the taxon [9, 10]. By and large, the transcriptome projects involving molluscs have included commercially exploited species and/or endangered species. This includes the critically endangered land snail, *Satsuma myomphala* [11]; Korea endangered freshwater pearl bivalve, *Cristaria plicata* [12], Australian mollusc, *Dicathais orbita* [13], Sydney rock oyster, *Saccostrea glomerata* [14], and the freshwater snail, *Oncomelania hupensis* [15], among others. The above studies identified genes with major impacts on ecologically relevant traits. Furthermore, transcriptome analysis has been useful in large-scale screening of microsatellites such as the simple sequence repeats (SSRs) and single nucleotide polymorphisms (SNPs). In the case of molluscan species, these microsatellites would be valuable in genetic improvement of stocks through marker-assisted breeding and understanding the diversity of the species within and among populations [16, 17]. In our earlier studies, we have taken the lead in cataloguing the genomic resources for Korean endemic land snails, *Aegista chejuensis* and *Aegista quelpartensis* [18], and the threatened snail, *Ellobium chinense* [19] as part of prioritized conservation efforts in South Korea.

In the present study, the Illumina HiSeq 4000 platform was used to acquire and annotate the transcriptome of air-breathing land slug, *Incilaria fruhstorferi*. This is to investigate the ecological dynamics of the species through screening of growth, immunity, and reproduction related genes. We conducted a de novo assembly of the transcriptome, screened the ORF containing transcripts, and annotated the same to homologous sequences in nucleotide and protein databases including the locally curated Protostome database (PANM-DB) [20]. We screened repeats predominant in the transcriptome and identified potential SSR markers for effective use in population genetic studies. Furthermore, we investigated genes related to growth, reproduction, and immune function with special reference to the pathogen recognition receptor PGRP-SC2 and Toll-interacting protein, also known as Tollip, using phylogenetic analysis. Hence, by utilizing paired-end (PE) Illumina transcriptome sequencing, we provide the first set of genomic information in the non-model land slug *I. fruhstorferi*. Further, with the screening of adaptation related genes, we investigate the plasticity of the species to sustain in varied climatic zones through compatibility in terms of biological, ecological, and physiological responses. Further, we have screened candidate genes involved in Toll signalling pathway, apoptotic pathway, and inflammatory response pathways. We assume that the transcriptome resources would provide unbiased access to phenotypic screens of many traits in the species. This would promote beneficial hybridization as a means of conservation planning.

## Methods

### Sample collection

**Fig. 1:**
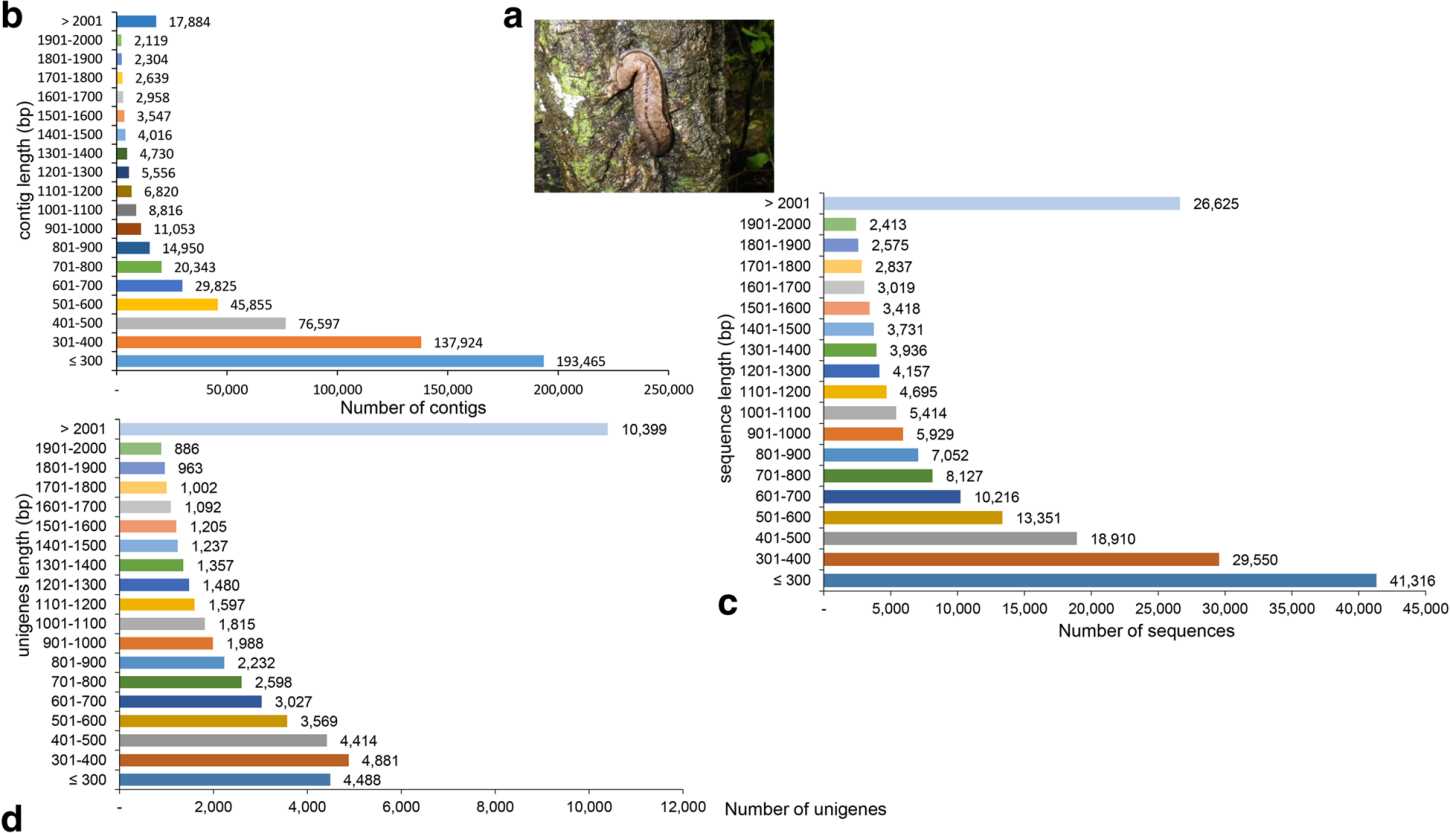
Size distribution of *I. fruhstorferi* (**a**) visceral mass assembled sequences. The clean reads were clustered using the Trinity de novo assembly, redundancy from the clustered sequences were removed using the TransDecoder program, defining the potential coding regions. (**b**) Contig length distribution, (**c**) Non-redundant sequences length distribution, (**d**) unigene length distribution

Specimens of *I. fruhstorferi* were collected during July 2015, from a location close to Sammak-gil, Cheong-pyeong-ri, Buksan-myeon, Chuncheon-si, Gangwon-do province, Republic of Korea. A total of 3 specimens ranging from 5 to 7 g were collected. The air-breathing slug, *I. fruhstorferi* is shown in Fig. 1a. After transferring the specimens to the laboratory, the visceral mass tissue (containing all the organs of the digestive and reproductive system) were dissected and immediately placed into liquid nitrogen until RNA preparation. The study follows ethical principles of use of experimental animals in biomedical research [21].

### RNA extraction and Illumina sequencing

Total RNA was extracted from the visceral mass tissue using the TRIzol reagent according to manufacturer’s instructions, and treated with RNase-free DNaseI. The extracted RNA was checked for concentration, purity and integrity using NanoDrop-2000 spectrophotometer and 2100 Bioanalyzer. In this case, the concentration of RNA was 3.15 ng/μl in a volume of 18 μl = 56.7 ng/μl and was considered as RNA input for library preparation and downstream processing. mRNA-seq sample preparation kit (Illumina, CA, USA) was used to construct an mRNA-seq library following the manufacturer’s instructions. Briefly, mRNA was purified from total RNA using oligodT magnetic beads. The mRNA was broken into short fragments of 200 nt using an RNA fragmentation kit (Ambion, TX, USA). The short mRNA fragments were reverse-transcribed into first strand cDNA using random primers and reverse transcriptase and second strand cDNA with RNase H and DNA polymerase I. After purification using QIAquick PCR extraction kit, the cDNA was ligated to sequencing adapters with paired-end (PE) Adapter Oligo Mix using T4 DNA ligase. DNA fragments of the desired size (200 ± 25 bp) were sequenced on the Illumina HiSeq 4000 sequencing platform to generate 125 bp PE reads. All the raw data obtained from sequencing were registered with NCBI Sequence Read Archive (SRA) under SRR5936593, BioProject-PRJNA398441, and BioSample-SAMN07510659.

### Processing of sequencing data and de novo assembly

Before the processing of clean reads for downstream assembly and annotation analysis, the raw sequencing reads were cleaned for low-quality sequences (Cutadapt 1.18 with default parameters), such as the adapter sequences and repeated reads [22]. The quality of the raw reads in fastq format was assessed using FastQC software version 0.11.5 (http://www.bioinformatics.babraham.ac.uk). The clean reads, thus obtained were carried forward for transcriptome de novo assembly using the Trinity short read program under the default settings of a minimum allowed length of 200 bp. Under this program, the Illumina shortreads are clustered together to form the contigs (‘Inchworm’ assembly step), contigs clustered and processed to de Bruijin graph (‘Chrysalis’), and extraction of all probable sequences from individual components de Bruijin graphs that are parallelized (‘Butterfly’). Redundancy from the clustered datasets was removed using cd-hit-est version 4.6.6 [23], with TransDecoder release version 2.0.3 (https://github.com/TransDecoder/TransDecoder/wiki) as a protein-prediction tool. BUSCO version 3.0.1 [24] using the metazoan_odb9 [25] was used to create a lineage dataset and assess the completeness of the transcriptome.

### Homology search and functional annotation

The non-redundant unigenes were annotated to the locally curated comprehensive protein database called PANM reference database (PANM-DB) using BLASTx. The database version 2.0 release contained a total of 7,571,246 protein sequences covering the Protostomes (Arthropoda, Mollusca, and Nematoda) [26]. BLASTx searches with an E-value threshold of 1.0E-5 were also used to identify homologous sequences in the Swiss-Prot protein sequence and UniGene nucleotide sequence databases. The EuKaryotic Orthologous Groups (KOG)-DB was also scanned for understanding the categorization of unigenes to specific functional descriptors such as ‘Cellular Processes and Signalling’, ‘Information Storage and Processing’, ‘Metabolism’, and ‘Poorly characterized’ (https://www.ncbi.nlm.nih.gov/COG/). The InterProScan feature of BLAST2GO professional suite (https://www.blast2go.com) was utilized to annotate the conserved domains in the unigenes. Further, the Gene Ontology (GO) annotations were retrieved with an E-value threshold of 1.0E-5 and classified into three categories at level 2 (biological process, cellular component, and molecular function). The GO functional classification of the unigenes was finally plotted using Web Gene Ontology Annotation Plot version 2.0 (WEGOv2.0) software [27]. Pathway analysis of unigene sequences were analyzed using the Kyoto Encyclopaedia of Genes and Genomes (KEGG) database release 84.0 (http://www.genome.jp/tools/kaas/).

### Gene discovery related to immunity, reproduction, and growth

A keyword search was employed to identify the candidate genes (involved in *I. fruhstorferi* immunity, reproduction, and growth) from the BLASTx annotated PANM-DB. Keywords included representative names of genes involved in different stages of molluscan immunity, cell signalling process, sex-determination, reproduction, and growth. Further, the GO terms and KEGG classification information was also utilized to identify the putative function of the gene products of the transcripts (the translated proteins). A comprehensive network of immunity-related transcripts was categorized under ‘Pathogen Recognition Receptor’ , ‘TLR Signalling Pathway (Adapter proteins, MyD88-dependent pathway)’ , ‘Endogenous Ligands’ , ‘Immune Effectors’ , ‘Antimicrobial Peptides’ , ‘Cytokines and Cytokine Receptors’ , and ‘Apoptosis’.

For evaluating the assembly and annotation obtained for *I. fruhstorferi*, we performed PCR-sequencing based approach. Total RNA was extracted from *I. fruhstorferi* whole- body using the Trizol method. cDNA was synthesized using AccuPower RT PreMix (Bioneer, Korea) with Oligo (dT)_12–18_ primer on a MyGenie 96 thermal cycler (Bioneer, Korea). To screen the transcriptome-derived unigenes for Tollip and PGRP-SC2, we generated a database using the FASTA file derived from NCBI with the keyword “gastropoda + gene name”. Subsequently, we used the database for local-BLAST to find the unigene which has the target gene. The screened Tollip and PGRP-SC2 sequences were used as the subject sequence for primer designing using Primer3 ver 4.0 (http://bioinfo.ut.ee/primer3-0.4.0/). The primers for PCR validation are as follows: Fwd-5′-ACTTTCTGCGTGCTGGATCT-3′ and Rev- 5′-TTCCTACACGCACTCGACAG-3′ (Tollip); Fwd-5′-CCAGTATTTTTGACACCAAGTTCA-3′ and Rev-5′-GGCCAGGTTCTTATCTCTTGG-3′ (PGRP-SC2). β-actin gene was considered as an endogenous control (Fwd-5′-GTGCCAGACGCCTAACAGTA-3′ and Rev-5′-GATGTCACGAACAATCTCACG-3′). PCR (35 cycles) was conducted using PowTaq polymerase (GnC company, Daejeon, Korea) at an annealing temperature of 54 °C.

### Bioinformatics analysis

The *I. fruhstorferi* unigenes showing homology to Tollip and PGRP-SC2 sequences in PANM DB were subjected to FGENESH gene-finding software using the online Softberry suite [28]. The ORF sequences confirmed using the online tool ORFPredictor [29] were used as queries against the NCBInr database to find the homologous sequences. Once validated, the translated amino acid sequences were used as query for the predictive analysis of protein sequence and structure. The physicochemical properties of the protein sequences were analysed using DNASTAR LASERGENE ver.7.1 Protean tool (http://www.dnastar.com/). SignalP at http://www.cbs.dtu.dk/services/SignalP/ was used to examine the presence of signal peptide. Transmembrane regions were predicted using the TMHMM Server v.2.0 (http://www.cbs.dtu.dk/services/TMHMM/). The domain architecture of the protein sequences were retrieved using the SMART domain analysis program at http://smart.embl-heidelberg.de/. The secondary structure was predicted using PSIPRED program at http://bioinf.cs.ucl.ac.uk/psipred_new/. All the pairwise and multiple sequence alignments were performed using ClustalX2 program [30]. The alignment files (. aln extension) generated were analyzed in GeneDoc sequence visualization software for Windows [31]. The phylogenetic tree was constructed using the maximum-likelihood method with the bootstrap trials set to 1000. The phylogenetic tree was visualized using the Molecular Evolutionary Genetics Analysis (MEGA) ver7.0 suite at https://www.megasoftware.net/.

### Repeats and microsatellite marker discovery

The Perl script program MicroSAtellite (MISA) (http://pgrc.ipk-gatersleben.de/misa/) was used to detect Simple Sequence Repeats (SSRs) as di-, tri-, tetra-, penta-, and hexanucleotide repeats from unigene sequences >2Kb. The homology-based repeat search program, RepeatMasker (version 4.0.6) was used to screen representative repeats such as the ‘Short Interspersed Nuclear Elements (SINEs)’, ‘Long Interspersed Nuclear Elements (LINEs)’ , ‘Long Terminal Repeat (LTR) elements’, and ‘DNA elements’ https://www.girinst.org/ (http://ftp.genome.washington.edu/RM/RepeatMasker.html).

## Results

### Sequencing reads, quality control, and de novo assembly

A mRNA-Seq library was constructed after isolation of Total RNA from the visceral mass tissue of *Incilaria fruhstorferi*. The total number of raw reads achieved for *I. fruhstorferi* transcriptome was 121,442,128. The pre-processing pipeline of raw reads included the trimming of the adapter sequences from the read pairs using the Cutadapt program, filtering low-quality reads (> 50% of bases having Q-value ≤20), and ambiguous bases (Additional file 1: Table S1). On an average, 0.6% of reads were discarded and length of reads after trimming was 300.2 bp. A total of 117,541,612 high-quality sequencing reads (16,896,672,304 bases) that constitutes 96.79% sequences (92.14% bases) were obtained from the transcriptome quality control. De novo assembly of the clean sequence reads was conducted using the Trinity assembly, generating 591,401 contigs with the largest contig size of 15,717 bp. About 31.12% of the contigs were considered ≥ 500 bp in length, with an overall mean length of 559.3 bp. Out of the total of 591,401 contig sequences, 197,271 sequences with potential ORF were screened including 107,658 sequences of lengths ≥500 bp. TGICL (TIGR gene indices clustering tool) clustering of the assembled sequences with potential open reading frames (ORF) identified 50,230 unigene sequences (67,667,335 bases), with the smallest to largest unigenes ranging from 135 bp to 26,139 bp. A statistical summary of Trinity assembly, TransDecoder utility, and the de novo assembled unigenes is depicted in Table 1. Further, the unigenes showed a greater length distribution when compared with the contigs and the assembled sequences. Almost 32.71% of contigs were ≤ 300 bp and only 3.02% was above 2001 bp (Fig. 1b). This is in contrast to 20.94% (≤ 300 bp) and 13.5% (> 2000 bp) in the ORF containing transcripts obtained by TransDecoder program (Fig. 1c). Almost 20.7% unigenes showed lengths of > 2000 bp, increasing the possibility of obtaining full-length transcripts (Fig. 1d). Based on BUSCO, we assessed the “completeness” of the *I. fruhstorferi* transcriptome. A total of 978 number BUSCOs were searched, out of which 691 (91.8%) were complete and single-copy BUSCOs, 206 complete and duplicated BUSCOs (21.1%), 50 (5.1%) fragmented, and 31 (3.1%) missing BUSCOs.

**Table 1:** Statistical summary of *Incilaria fruhstorferi* transcriptome

Total number of raw reads	
-Number of sequences	121,442,128
-Number of bases	18,337,761,328
Total number of clean reads	
-Number of sequences	117,541,612
-Number of bases	16,896,672,304
-Mean length of contig (bp)	143.8
-N50 length of contig (bp)	151
-GC % of contig	40.87
High-quality reads (%)	96.79 (sequences), 92.14 (bases)
Contig information	
-Total number of contig	591,401
-Number of bases	330,746,158
-Mean length of contig (bp)	559.3
-N50 length of contig (bp)	637
-GC % of contig	38.04
-Largest contig (bp)	15,717
-No. of large contigs (≥ 500 bp)	184,017
After TransDecoder	
-Total number of sequence	197,271
-Number of bases	202,308,633
-Mean length of sequence (bp)	1025.5
-N50 length of sequence (bp)	1757
-GC % of sequence	39.87
-Largest sequence (bp)	15,717
-No. of large sequence (≥ 500 bp)	107,658
Unigene information	
-Total number of unigenes	50,230
-Number of bases	67,667,335
-Mean length of unigene (bp)	1347.1
-N50 length of unigene (bp)	2069
-GC % of unigene	39.72
-Length ranges (bp)	135–26,139

### Sequence annotation of unigenes for homologous matches

**Fig. 2:**
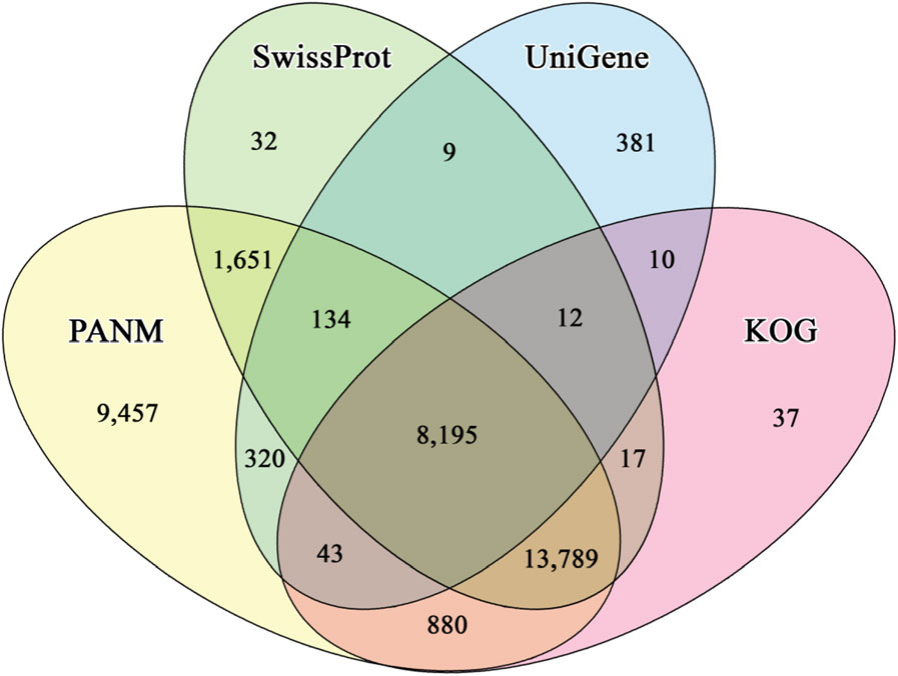
Annotation of *I. fruhstorferi* unigenes against the public protein and nucleotide databases. Venn diagram showing homologous matches of unigenes to PANM DB, UniGene, SwissProt, and KOGDB (either specific or overlapping)

Of the 50,230 unigenes, 34,470 (68.62%) sequences showed matches to homologous sequences in PANM-DB at an E-value cut-off of 1.0E-5, followed by 16,629 (33.11%) in Swiss-Prot, and 6624 (13.19%) in NCBI’s UniGene database. Annotation statistics of *I. fruhstorferi* unigenes against the public protein and nucleotide databases is represented in Table 2. In total, 35,204 unigenes (70.09%) showed matches to homologous sequences in the databases. Out of the annotated sequences, 20,174 sequences (57.31%) showed lengths of ≥ 1000 bp. Most of the unigenes (34,470) showed homologous matches to PANM-DB. Further, 15,760 sequences (31.38%) showed no homologous matches to the database. Possibly, most of the non-annotated sequences are shorter with lesser likelihood of conserved domains and motifs. Further, the non-annotation can be ascribed to orphaned untranslated regions (UTRs), sequences from uncharacterized genes, or sequences unique to *I. fruhstorferi*. The Venn diagram illustrated in Fig. 2 reveals that 9457 sequences (27.44%) out of the 34,470 show specific annotation to the homologous proteins in PANM-DB. A total of 13,789 (40%) sequences, those that annotated to homologous sequences in PANM-DB also found hits in SwissProt and KOG DB, and 8195 sequences (23.77%) annotated to all the four databases. A greater number of sequence annotation hits including the unique hits were represented under PANM-DB.

**Table 2:** Annotation of *I. fruhstorferi* unigene sequences against public protein and nucleotide databases. The unigenes are classified based on their sizes

Databases	All transcripts	≤ 300 bp	300–1000 bp	≥ 1000 bp
PANM-DB	34,470	2118	12,429	19,923
UNIGENE	9105	281	2200	6624
SwissProt	23,840	611	6600	16,629
KOG	22,984	616	6382	15,986
GO	15,660	382	3721	11,557
KEGG	81	–	13	68
IPS	18,238	481	4711	13,046
ALL	35,204	2186	12,844	20,174

### Homology characteristics and functional annotation of unigenes

BLASTx analysis was performed using 34,470 unigenes against PANM-DB to characterize the homology matrices (Additional file 2: Figure S1). The E-value distribution reveals that 44.59% of the sequences show homology at 1.0E-50 to 1.0E-5 (Additional file 2: Figure S1A). Majority of unigenes (37.15%) show an identity of 40–60%, while only 41 sequences (0.12%) show 100% identity to homologous sequences in PANM-DB (Additional file 2: Figure S1B). The highest number of unigenes (14,710 sequences; 42.67%) showed similarities in the range 60–80%, while only 111 sequences (0.32%) showed 100% similarity with the database sequences (Additional file 2: Figure S1C). The number of annotation hits as compared to no-hits increased in direct proportion to the length of unigenes (Additional file 2: Figure S1D). Sequences of lengths > 2001 bp showed the highest 9867 hits. It is obvious that with longer sequences the annotation improves because they are attributed with conserved protein domains representative of protein function. Further, considering the top-hit species distribution, 44.16 and 30.58% of the unigenes matched *Aplysia californica* and *Biomphalaria glabrata*, respectively (Additional file 3: Figure S2).

**Fig. 3:**
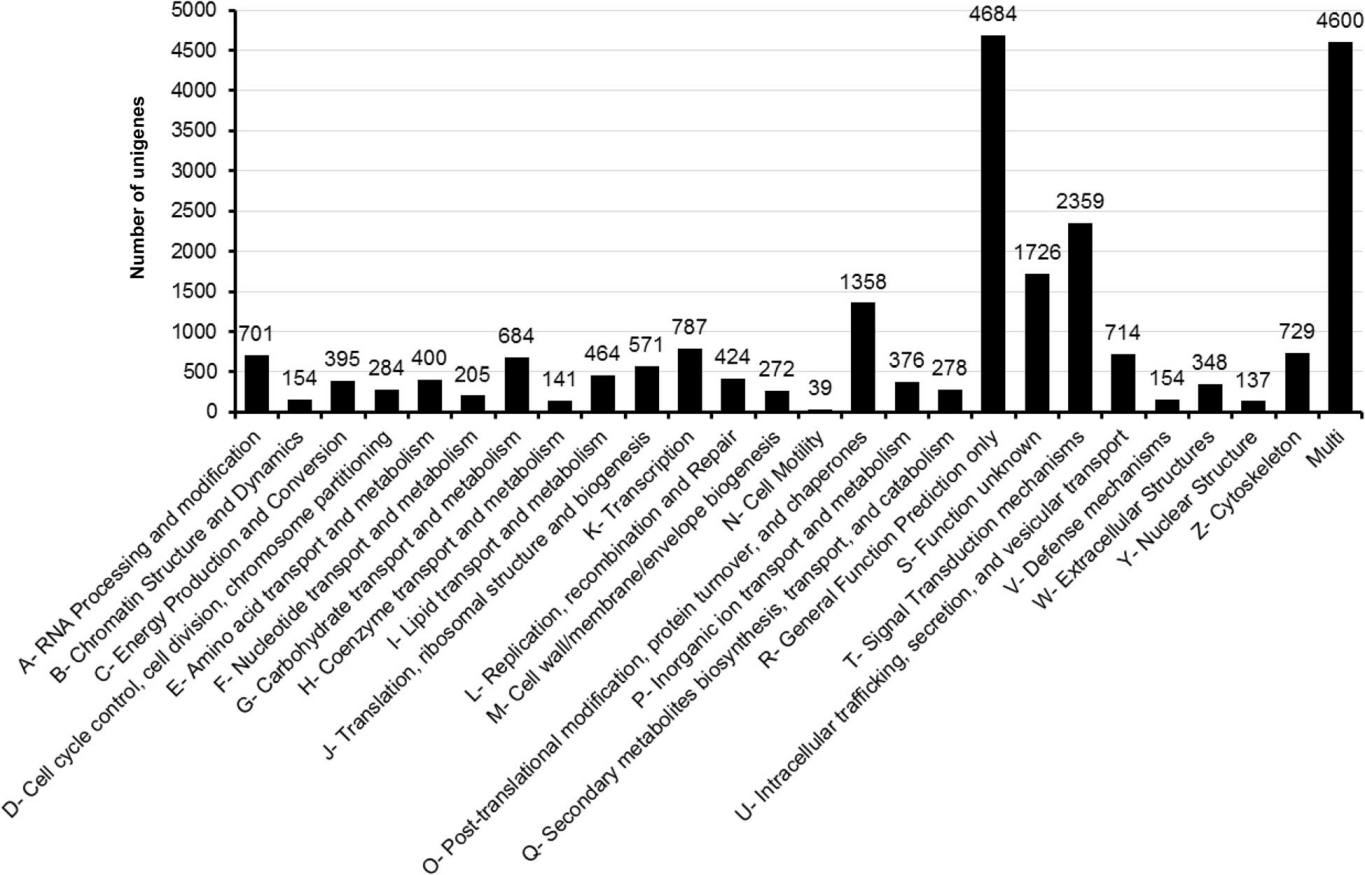
KOG functional classification of *I. fruhstorferi* unigenes. Of the 50,230 non-redundant unigene sequences, 22,984 classified under 25 functional KOG categories excluding the multifunctional category. Predominantly, the unigenes classified under R- General function and Multi category

Of the 50,230 unigenes, 18,238 showed classic and conserved InterPro domains. A total of 1271 unigenes showed the predominant Zinc finger (Znf), C2H2-type domain, followed by 985 and 703 sequences showing the P-loop containing nucleoside triphosphate hydrolase and Zinc finger RING/FYVE/PHD-type domains, respectively (Table 3). Other protein domains predominantly noticed in *I. fruhstorferi* unigene sequences include Protein kinase-like, Immunoglobulin-like fold, EGF-like, Galactose-binding, and Leucine-rich repeats. Further, to explore the functional direction of *I. fruhstorferi* unigenes, we annotated the sequences against the KOG, GO, and KEGG databases. A total of 22,984 sequences were annotated in the KOG database, out of which 69.55% of sequences had lengths of ≥ 1000 bp. Further, the sequences were classified under 25 KOG functional categories (Fig. 3). In the present annotation, 20.38% of unigenes were classified under R term (General function prediction only), followed by 10.26 and 7.51% under T (Signal transduction mechanisms) and S (function unknown) terms, respectively. A high proportion (20.01%) of sequences also classified to the ‘multi’ category (sequences belonging to more than one functional term). The least populated KOG terms included H (co-enzyme transport and metabolism), Y (nuclear structure), and N (cell motility) with 141, 137, and 39 unigene sequences, respectively.

**Table 3:** List of top-20 protein domains found in *I. fruhstorferi* unigene sequences

Domain	Description	Unigene (Nos.)
IPR013087	Zinc finger C2H2-type	1271
IPR027417	P-loop containing nucleoside triphosphate hydrolase	985
IPR013083	Zinc finger, RING/FYVE/PHD-type	703
IPR012337	Ribonuclease H-like domain	664
IPR000477	Reverse transcriptase domain	517
IPR011009	Protein kinase-like domain	478
IPR013783	Immunoglobulin-like fold	451
IPR000742	EGF-like domain	445
IPR000719	Protein kinase domain	416
IPR015943	WD40/YVTN repeat-like-containing domain	401
IPR002156	Ribonuclease H domain	381
IPR011989	Armadillo-like helical	372
IPR017986	WD40-repeat-containing domain	368
IPR016024	Armadillo-type fold	367
IPR016040	NAD(P)-binding domain	343
IPR008979	Galactose-binding domain-like	337
IPR000504	RNA recognition motif domain	323
IPR011990	Tetratricopeptide-like helical domain	313
IPR032675	Leucine-rich repeat domain, L domain-like	303
IPR020683	Ankyrin repeat-containing domain	293

**Fig. 4:**
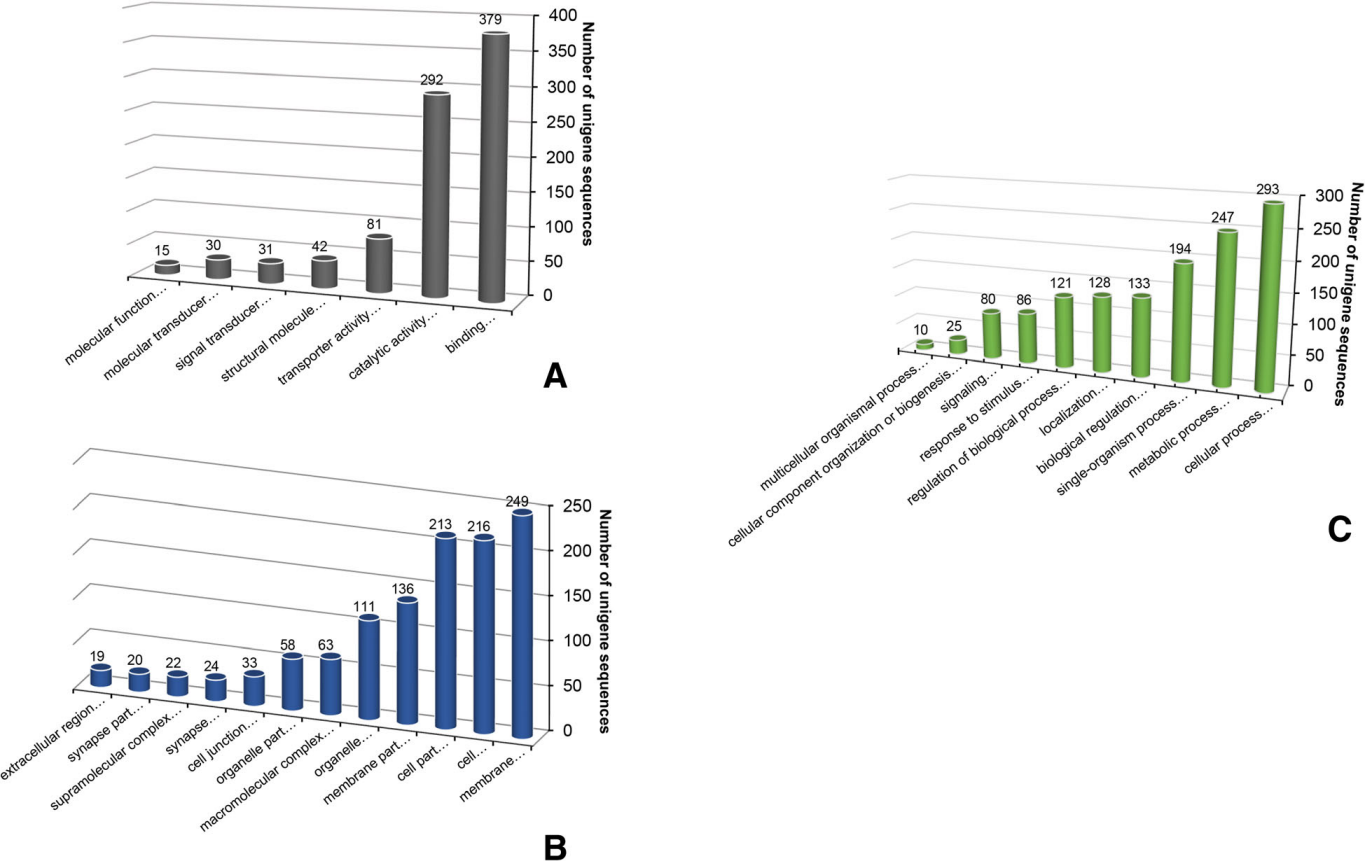
GO classification of *I. fruhstorferi* unigenes at level 2. The unigene sequences classified under different functional categories under the GO term notation of (**a**) Molecular Function, (**b**) Cellular Component, and (**c**) Biological Process

For high-quality functional analysis of genomic datasets, we used the bioinformatics platform Blast2GO v5.1 (https://www.blast2go.com/). GO-based annotation and KEGG pathway maps for the *I. fruhstorferi* genomic datasets were elucidated using the Blast2GO software platform. A total of 15,660 unigenes were annotated to GO functional categories such as ‘Molecular function’ , ‘Biological process’, and ‘Cellular component’ (Additional file 4: Figure S3). The maximum 32.63% of the sequences were classified under ‘Molecular function’ category followed by only 4.27 and 3.21% sequences classified under ‘Biological process’ and ‘Cellular component’ category, respectively. Further, 26.79% of sequences were classified to both ‘Biological Process’ and ‘Molecular function’ categories. A total of 2790 sequences (17.82%) were classified under all the three GO functional categories (Additional file 4: Figure S3A). Only 38.55% of sequences annotated against GO database were categorized under a single GO term (Additional file 4: Figure S3B). Rest of the sequences participated under more than one GO term. The GO terms (at level 2) for each of the three categories are shown in Fig. 4. Within the ‘Molecular function’ category, the unigenes predominantly were predicted under binding (GO: 0005488), followed by catalytic activity (GO: 0003824) and transporter activity (GO: 0005215) (Fig. 4A). In the ‘Cellular component’ category, the top-represented GO terms included membrane (GO: 0016020), cell (GO: 0005623), and cell part (GO: 0044464) (Fig. 4B). Under ‘Metabolic process’ category, the most prominent GO terms included cellular process (GO: 0009987), metabolic process (GO: 0008152), and single-organism process (GO: 0044699) (Fig. 4C). We further annotated the assembled genomic sequences of *I. fruhstorferi* to associated biological pathways using the KEGG database. Only 81 sequences of enzymes were classified under ‘Environmental Information Processing’ , ‘Metabolism’, and ‘Organismal Systems’ categories. These sequences are related to the 46 putative enzymes in the pathway (Table 4).

**Table 4:** KEGG Pathway distribution

KEGG pathways	Enzymes in the pathway	Sequences of enzymes
Environmental Information Processing		
Signal transduction		
Phosphatidylinositol signalling system	2	6
Metabolism		
Amino acid metabolism		
Alanine, aspartate and glutamate metabolism	1	1
Arginine biosynthesis	1	1
Cysteine and methionine metabolism	1	1
Phenylalanine metabolism	1	3
Phenylalanine, tyrosine and tryptophan biosynthesis	1	3
Biosynthesis of other secondary metabolites		
Neomycin, kanamycin and gentamicin biosynthesis	1	2
Streptomycin biosynthesis	1	2
Carbohydrate metabolism		
Amino sugar and nucleotide sugar metabolism	1	2
Citrate cycle (TCA cycle)	1	1
Fructose and mannose metabolism	2	3
Galactose metabolism	1	2
Glycolysis / Gluconeogenesis	2	4
Glyoxylate and dicarboxylate metabolism	1	1
Inositol phosphate metabolism	1	2
Pentose and glucuronate interconversions	1	1
Starch and sucrose metabolism	1	2
Energy metabolism		
Carbon fixation in photosynthetic organisms	1	2
Nitrogen metabolism	2	2
Global and overview maps		
Biosynthesis of antibiotics	4	6
Glycan biosynthesis and metabolism		
Mucin type O-glycan biosynthesis	1	1
N-Glycan biosynthesis	1	1
Other glycan degradation	1	1
Various types of N-glycan biosynthesis	1	1
Metabolism of cofactors and vitamins		
Folate biosynthesis	1	3
Porphyrin and chlorophyll metabolism	2	3
Thiamine metabolism	1	34
Metabolism of terpenoids and polyketides		
Terpenoid backbone biosynthesis	1	1
Nucleotide metabolism		
Purine metabolism	5	41
Xenobiotics biodegradation and metabolism		
Aminobenzoate degradation	1	3
Drug metabolism – other enzymes	1	1
Organismal Systems		
Immune system		
T cell receptor signalling pathway	2	5
Th1 and Th2 cell differentiation	1	2

### Characterization of repeating elements and microsatellites in *I. fruhstorferi* transcriptome

**Fig. 5:**
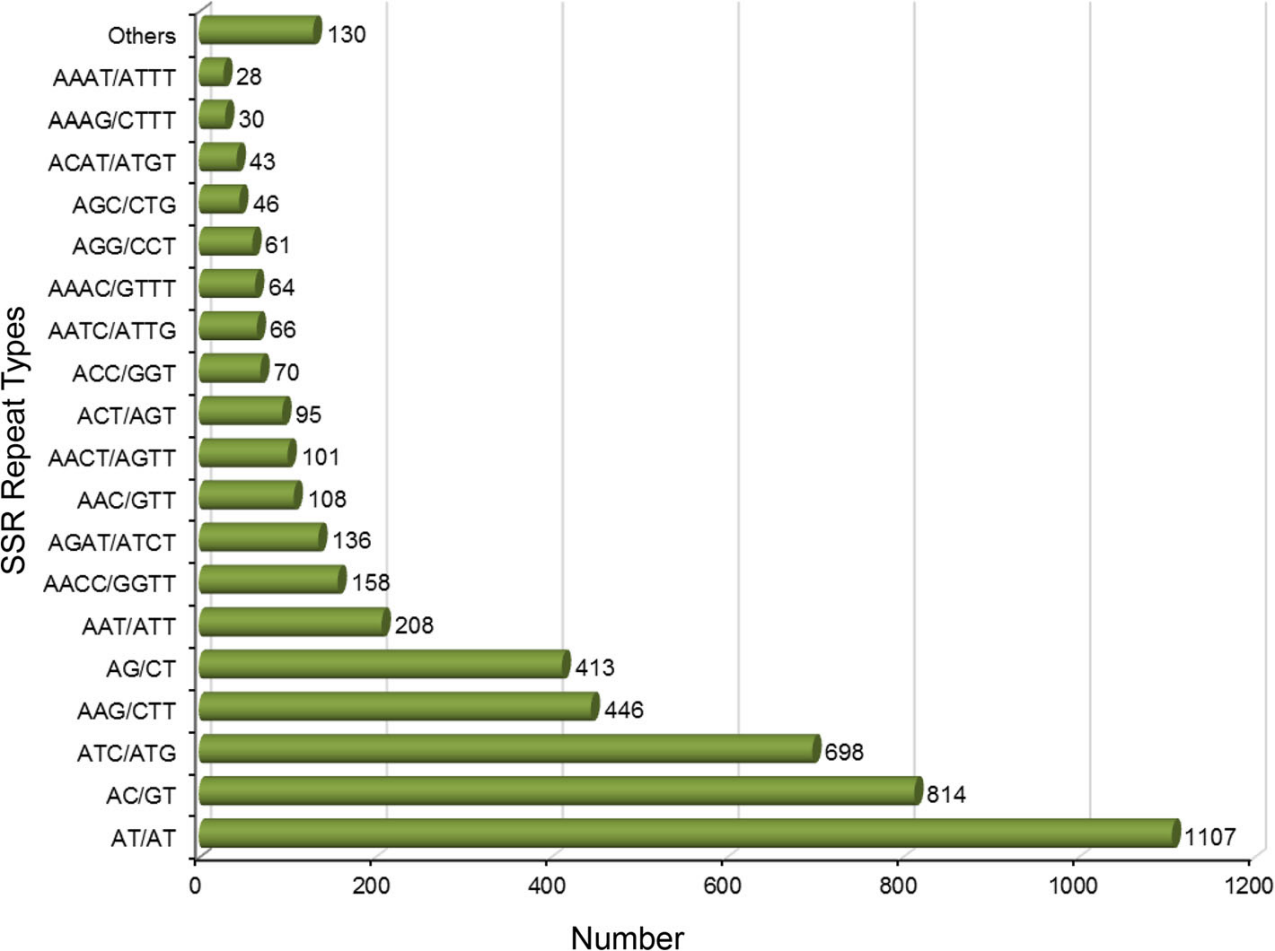
Distribution of Simple Sequence Repeat (SSR) types found in the unigenes of *I. fruhstorferi*. The most predominant repeat types included the dinucleotides AT/AT and AC/GT

Repeating elements promiscuous in the unigene sequences of *I. fruhstorferi* were analyzed using the Repeatmasker program (*Homo sapiens* as the species parameter) under the RepBase database (http://www.girinst.org). As suggested in Table 5, the prominent repeating elements found were the LINEs, DNA repeating elements such as hAT-Charlie and TcMar-Tigger, Simple repeats, and Low complexity regions. Under the SINEs, ALU (7 elements) and MIR repeat (4 elements) occupied lengths of 1593 and 253 bp, respectively. LINE repeats such as LINE1, LINE2, and L3/CR1 repeats were found occupying lengths of 11,676 bp, 822 bp, and 1196 bp, respectively. Among the retrotransposons, lesser number of LTR elements were found as compared to the non-LTR LINE elements. In the present study, the 50,230 assembled unigene sequences were screened for SSR markers using MISA (MicroSAtellite identification tool) (Table 6). A total of 4822 SSRs were identified in 3888 unigene sequences with 700 sequences containing more than 1 SSR. These SSRs were then classified on the basis of number of repeats to di-, tri-, tetra-, penta-, and hexanucleotide repeats. Mononucleotide repeats were not considered for analysis due to the possibility of homopolymer formation during Illumina sequencing. A maximum of dinucleotide repeats were noticed followed by trinucleotides and tetranucleotide repeats. Dinucleotide repeats existed in maximum six iterations, trinucleotides in maximum five iterations, tetra- and pentanucleotide repeats in maximum of four iterations. Further, in an attempt to classify the SSR repeat types, it was found that the dinucleotide repeat AT/AT (1107 SSR) and AC/GT (814 SSR) were the most predominant. Among the trinucleotide repeats, ATC/ATG and AAG/CTT with 698 and 446 SSRs were the dominant. AACC/GGTT and AGAT/ATCT repeats with 158 and 136 SSRs were the dominant tetranucleotide repeats. A summary of SSR repeat types is shown in Fig. 5.

**Table 5:** RepeatMasker based analysis of repeating elements in the *Incilaria fruhstorferi* unigenes

		Number of elements^a^	Length occupied	Percentage of sequence
SINEs:		12	1932 bp	0.00%
	ALUs	7	1593 bp	0.00%
	MIRs	4	253 bp	0.00%
LINEs:		642	125,258 bp	0.19%
	LINE1	78	11,676 bp	0.02%
	LINE2	14	822 bp	0.00%
	L3/CR1	19	1196 bp	0.00%
LTR elements:		28	5015 bp	0.01%
	ERVL	6	435 bp	0.00%
	ERVL-MaLRs	4	248 bp	0.00%
	ERV_class I	8	3489 bp	0.01%
	ERV_class II	6	501 bp	0.00%
DNA elements:		283	47,370 bp	0.07%
	hAT-Charlie	122	15,876 bp	0.02%
	TcMar-Tigger	55	7272 bp	0.01%
Unclassified:		2	102 bp	0.00%
Total interspersed repeats:			179,677 bp	0.27%
Small RNA:		55	4190 bp	0.01%
Satellites:		26	15,698 bp	0.02%
Simple repeats:		9067	407,633 bp	0.60%
Low complexity:		1481	77,420 bp	0.11%

^a^most repeats fragmented by insertions or deletions have been counted as one element

RepBase Update 20,160,829; RM database version 20,160,829

**Table 6:** Screening of Simple Sequence Repeats (SSRs) from unigene sequences of *Incilaria fruhstorferi*

Total number of sequences examined	50,230
Total size of examined sequences (bp)	67,667,335
Total number of identified SSRs	4822
Number of SSR containing sequences	3888
Number of sequences containing more than 1 SSR	700
Number of SSRs present in compound formation	471
Unit size	Number of SSRs
2	2335
3	1738
4	704
5	40
6	5

### Candidate genes in *I. fruhstorferi* immune defense system

A keyword search composed of a series of representative innate immunity and oxidative stress genes was used to screen candidate unigenes putatively responsible for *I. fruhstorferi* immune defense. The GO term and KEGG classification also provided sufficient information regarding the representative genes classified under “immune system”. An exhaustive summary of the candidate genes under immune defense categories such as ‘pathogen recognition receptor (PRR)’ , ‘TLR signalling pathway’, ‘adaptor proteins’ , ‘MyD88-dependent pathway’ , ‘endogenous ligands’ , ‘immune effectors’ , ‘antimicrobial peptides’ , ‘cytokines and cytokine receptors’ , ‘apoptosis-related factors’ , and ‘others’ are summarized (Additional file 5: Table S2). Overall, we were able to retrieve the extensive repertoire of genes that could be relevant to understand the molluscan innate immune signalling process and the specific host defense system against pathogens. Furthermore, genes involved in the unique features of the slug’s physiology have been summarized in Table 7. We would like to emphasize that the screening of heat shock protein and the aquaporin family genes from *I. fruhstorferi* transcriptome can be scaled-up for functional genomics approaches to understand the physiological rules of adaptation in the species.

**Table 7:** Genes of interest related to Adaptation/Physiology in the land slug, *Incilaria fruhstorferi*

Candidate genes	Unigenes ID	Length (bp)
Angiotensin-converting enzyme-like	If_Uni_08052, If_Uni_10667, If_Uni_38048	2711, 2722, 2206
Type-1 angiotensin II receptor-associated protein-like	If_Uni_23153	1732
Type-1 angiotensin II receptor B-like	If_Uni_28079, If_Uni_28268	2354, 2158
Adenylate cyclase isoform X1	If_Uni_27752, If_Uni_27753	4405, 4152
Adenylate cyclase type 2-like	If_Uni_27754, If_Uni_35219	2008, 2381, 1192
Adenylate cyclase	If_Uni_30138, If_Uni_32170, If_Uni_32295, If_Uni_35390, If_Uni_36571	2381, 1826, 2164, 400, 1026
5′-AMP-activated protein kinase	If_Uni_06408, If_Uni_10162, If_Uni_16111, If_Uni_17195, If_Uni_31806, If_Uni_45007, If_Uni_45226, If_Uni_49689	509, 2981, 1583, 5849, 1937, 1110, 1134, 416
Aquaporin, partial	If_Uni_13477, If_Uni_36565, If_Uni_43198	2249, 934, 2384
Aquaporin-4-like	If_Uni_27284, If_Uni_27285, If_Uni_40352, If_Uni_48767	2280, 2132, 475, 1184
Aquaporin AQPcic-like	If_Uni_30154, If_Uni_30155	1320, 1807
Aquaporin-8-like	If_Uni_30734	1971
Aquaporin AQPAn.G-like	If_Uni_37263, If_Uni_49973	1378, 1468
Aquaporin-9-like	If_Uni_39587	1799
Collagen alpha-1(I) chain-like	If_Uni_08744, If_Uni_08745, If_Uni_09485, If_Uni_09486, If_Uni_15928, If_Uni_27766, If_Uni_27767, If_Uni_29582, If_Uni_34918, If_Uni_37761, If_Uni_39405, If_Uni_39724	2643, 3984, 2558, 1269, 9047, 6483, 6691, 1652, 1063, 2081, 434, 2112
NMDA-type glutamate receptor	If_Uni_38285	1269
Glutamate receptor ionotropic, NMDA 2B-like	If_Uni_41604	278
Heat shock protein 70 B2-like	If_Uni_09052, If_Uni_09053, If_Uni_23324, If_Uni_23325, If_Uni_23326	1926, 3457, 2146, 2550, 2635
Heat shock 70 kDa protein cognate 4	If_Uni_23327, If_Uni_23328, If_Uni_33435, If_Uni_43426	2338, 2197, 2643, 2749
Heat shock 70 kDa protein 4	If_Uni_42286	4176
Heat shock 70 kDa protein 10	If_Uni_43360	285
Heat shock 70 kDa protein 13	If_Uni_36140, If_Uni_43584	879, 609
Heat shock 70 kDa protein 14	If_Uni_22068, If_Uni_22069, If_Uni_22070	1324, 4415, 4727
Hsp70-binding protein 1	If_Uni_41490	2947
Insulin receptor substrate 1	If_Uni_44383	1014
Mitogen-activated protein kinase 15	If_Uni_28827, If_Uni_28829	2104, 2417
calcium-independent phospholipase A2-gamma-like	If_Uni_28664, If_Uni_28665,	3187, 1485, 3167
group XV phospholipase A2-like	If_Uni_31585	1836
85/88 kDa calcium-independent phospholipase A2-like	If_Uni_32679, If_Uni_38324, If_Uni_41729	4506, 4556
stimulated by retinoic acid gene 6 protein	If_Uni_26478, If_Uni_26479, If_Uni_26480, If_Uni_26481, If_Uni_27267, If_Uni_27367, If_Uni_27368, If_Uni_27369, If_Uni_33908, If_Uni_46952	2296, 2213, 2242, 2205, 1938, 2115, 2198, 2107, 2373, 626
T-box transcription factor TBX20-like	If_Uni_21931, If_Uni_34429	3972, 1103
T-box transcription factor TBX1-B-like	If_Uni_25334, If_Uni_25335, If_Uni_03651, If_Uni_03826	2118, 1639, 2069, 2148
Solute carrier family 41	If_Uni_07293, If_Uni_24931, If_Uni_24932, If_Uni_24933, If_Uni_24934, If_Uni_24935, If_Uni_24936, If_Uni_35601	625, 2009, 2388, 2628, 1942, 2923, 2472, 978,
Solute carrier family 25	If_Uni_08851, If_Uni_08852, If_Uni_08853, If_Uni_08854, If_Uni_08855, If_Uni_10412, If_Uni_15275, If_Uni_15276, If_Uni_15277, If_Uni_15278, If_Uni_18168, If_Uni_30488, If_Uni_09808, If_Uni_33449, If_Uni_33450, If_Uni_34586, If_Uni_35960, If_Uni_38390, If_Uni_40358, If_Uni_41902, If_Uni_45240, If_Uni_47602, If_Uni_48696, If_Uni_00611	2061, 2136, 2163, 2157, 2185, 1962, 3220, 3019, 2473, 2614, 1212, 1614, 1552, 2559, 1424, 1249, 956, 767, 1736, 944, 822, 505, 1512, 2039
Solute carrier family 40	If_Uni_09262, If_Uni_09263, If_Uni_22596, If_Uni_22597, If_Uni_30690	2419, 2212, 3284, 3739, 1694
Solute carrier family 43	If_Uni_09267, If_Uni_09268, If_Uni_37142, If_Uni_43186, If_Uni_06623	2046, 1893, 2929, 2355, 2393
Solute carrier family 22	If_Uni_10312, If_Uni_15224, If_Uni_19826, If_Uni_22045, If_Uni_24463, If_Uni_24464, If_Uni_24465, If_Uni_24466, If_Uni_24467, If_Uni_24468, If_Uni_24469, If_Uni_26545, If_Uni_28975, If_Uni_28976, If_Uni_32419, If_Uni_32420, If_Uni_34348, If_Uni_36091, If_Uni_36557, If_Uni_36908, If_Uni_37399, If_Uni_37943, If_Uni_43214, If_Uni_49180, If_Uni_50058, If_Uni_00679, If_Uni_04935	2182, 1013, 1576, 326, 2087, 2244, 2088, 1748, 2140, 3025, 1801, 2738, 1543, 2640, 945, 612, 687, 758, 551, 1147, 1086, 1172, 272, 450, 1259, 236, 300,
Solute carrier family 23	If_Uni_12119, If_Uni_36506, If_Uni_37977, If_Uni_40499, If_Uni_47883	2100, 699, 2314, 2418, 1370
Solute carrier family 35	If_Uni_12656, If_Uni_12657, If_Uni_14403, If_Uni_19966, If_Uni_27046, If_Uni_27993, If_Uni_29331, If_Uni_29332, If_Uni_32495, If_Uni_35319, If_Uni_39687, If_Uni_46340, If_Uni_49558, If_Uni_00045, If_Uni_06869	1525, 1417, 2119, 1745, 3306, 2026, 676, 1184, 1375, 1347, 1611, 791, 1075, 1160, 1455
Solute carrier family 12	If_Uni_15628, If_Uni_15629, If_Uni_15630, If_Uni_15631, If_Uni_17088, If_Uni_17089, If_Uni_24560, If_Uni_24561, If_Uni_24562, If_Uni_24563, If_Uni_24564, If_Uni_24565, If_Uni_24566, If_Uni_24902, If_Uni_24903, If_Uni_24904, If_Uni_28839, If_Uni_30662, If_Uni_39565, If_Uni_45407, If_Uni_48974	4001, 3652, 4273, 4107, 3486, 1747, 3201, 3299, 2542, 3349, 3300, 3498, 3172, 4000, 5098, 6979, 1660, 2388, 713, 1364, 1204
Solute carrier family 28	If_Uni_19488, If_Uni_19489, If_Uni_19743, If_Uni_24733, If_Uni_24734, If_Uni_24735, If_Uni_26540, If_Uni_26541, If_Uni_26542	2683, 2556, 3035, 9234, 5184, 9389, 2972, 2846, 3184
Solute carrier family 15	If_Uni_27273, If_Uni_28577, If_Uni_30039, If_Uni_30040, If_Uni_31286, If_Uni_31287, If_Uni_32935, If_Uni_39335	2378, 2135, 2193, 1741, 3147, 3270, 1729, 357,
Solute carrier family 46	If_Uni_32274, If_Uni_43893	2899, 368,
Solute carrier family 13	If_Uni_34257, If_Uni_43800, If_Uni_48035	595, 552, 361
Solute carrier family 45	If_Uni_36376	1546

**Fig. 6:**
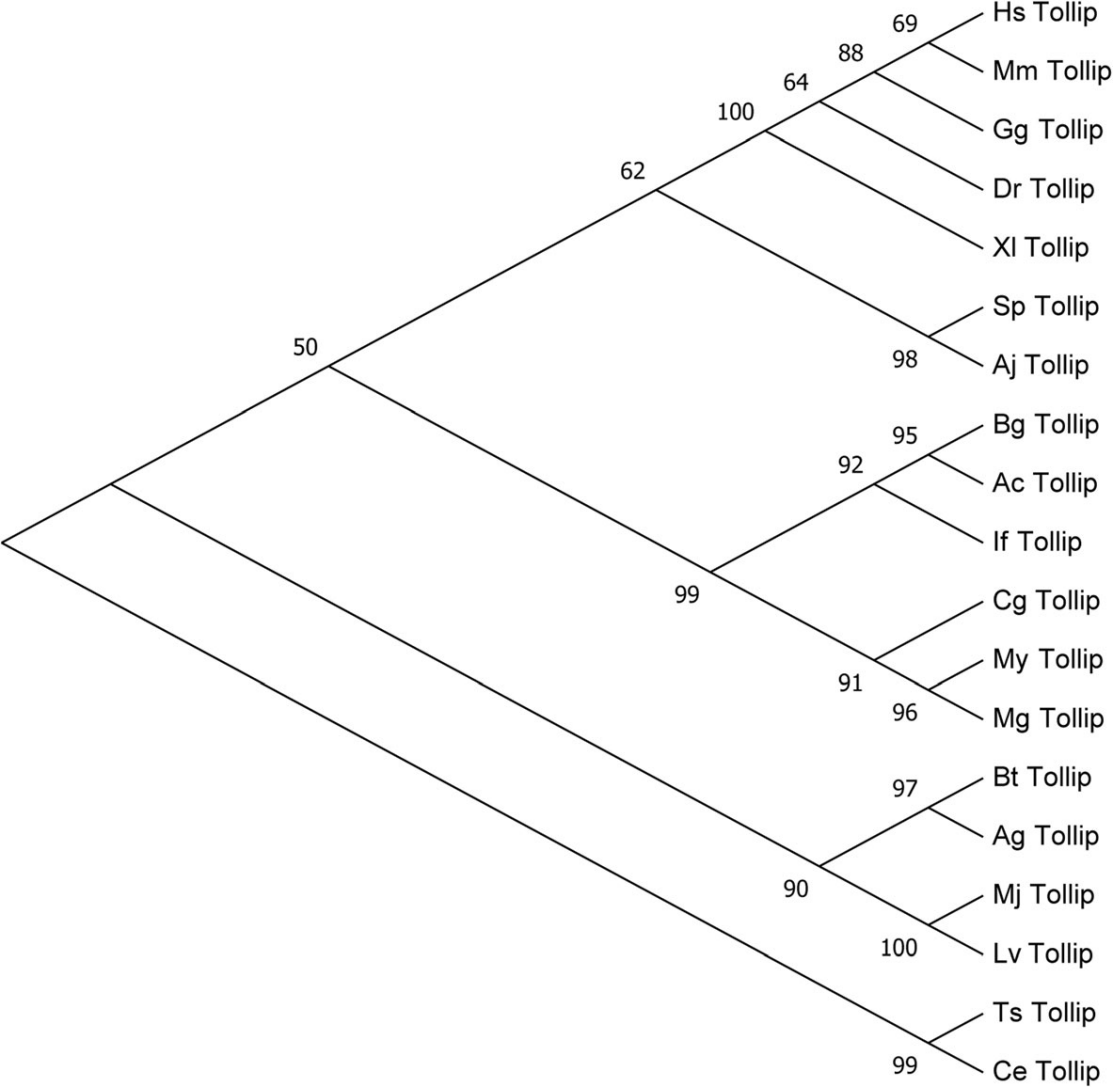
Phylogenetic relationships inferred for IfTollip using the maximum-likelihood method of MEGA7 program. The evolutionary distances were computed using the Poisson correction method. The tree was bootstrapped (1000 replications) and the values are indicated by numbers at nodes. GenBank accession numbers downloaded from Ensembl and NCBI are: HsTollip, Q9H0E2.1; MmTollip, Q9QZ06; GgTollip, F1P006; XlTollip, Q3B8H2;AjTollip, AHA83602.1; AgTollip, XP_562476; MjTollip, BAK19511; LvTollip, AET79206; CgTollip, EKC34473; MgTollip AHI17285; CeTollip, NP_492757; SpTollip, XP_001196148; DrTollip, AAH46009.1; PyTollip, AK062848.1; TsTollip, XP_003379806.1; BgTollip, XP_013073937.1 and AcTollip XP_012936924.1

From the transcriptome survey of *I. fruhstorferi*, we have screened unigenes homologous to PRRs such as the Toll-like receptors (TLRs), C-type lectins (CTLs), other lectin classes (I-type lectin, Malectin, Collectin, Selectin, Galectin), fibrinogen-related proteins (FRPs), and scavenger receptors (SRs), among others. Further, we have targeted the Tollip (IfTollip) and the Peptidoglycan Recognition Protein-SC2 (IfPGRP_SC2) for an elaborate in silico analysis covering the conserved domain architecture and phylogenetic position among orthologs. Further, to prove the accuracy of transcriptome results (validation of gene assembly and annotation), a PCR-sequencing based approach targeting IfTollip and IfPGRP-SC2 were successfully used. The sequences of PCR products thus obtained were aligned with the screened unigenes representing the target genes, revealing 100% identity (Additional file 6: Figure S4). This study did not include the functional characterization of IfTollip and IfPGRP_SC2 due to the difficulties in maintaining the experimental model and limited sample availability. We predicted the full-length ORF and the translated amino acid sequence of *I. fruhstorferi* Tollip (IfTollip) using bioinformatics analysis (Additional file 7: Figure S5). The IfTollip ORF composed of 840 bp encoding a polypeptide of 279 amino acids. The predicted molecular weight of IfTollip is 31.38 kDa, with an isoelectric point (pI) of 6.21. Multiple sequence alignment of Tollips from representative invertebrate and vertebrate species followed by SMART domain analysis revealed three conserved domains including Tom-1 binding domain (TBD), conserved core domain 2 (C2), and coupling of ubiquitin to endoplasmic reticulum degradation (CUE) domain (Additional file 8: Figure S6). Secondary structure prediction of IfTollip showed two and three α-helices at the N-terminal and the C-terminal (CUE domain), respectively (Additional file 9: Figure S7). The C2 domain was conspicuously represented by β-sheets and connecting loops. Furthermore, a phylogenetic tree was constructed using full-length amino acid sequences of Tollip from other species (Fig. 6). IfTollip was grouped closer to Tollips from molluscs showing most close association with *B. glabrata* and *A. californica*. This suggests that the evolutionary position of Tollip is conserved across phylum.

**Fig. 7:**
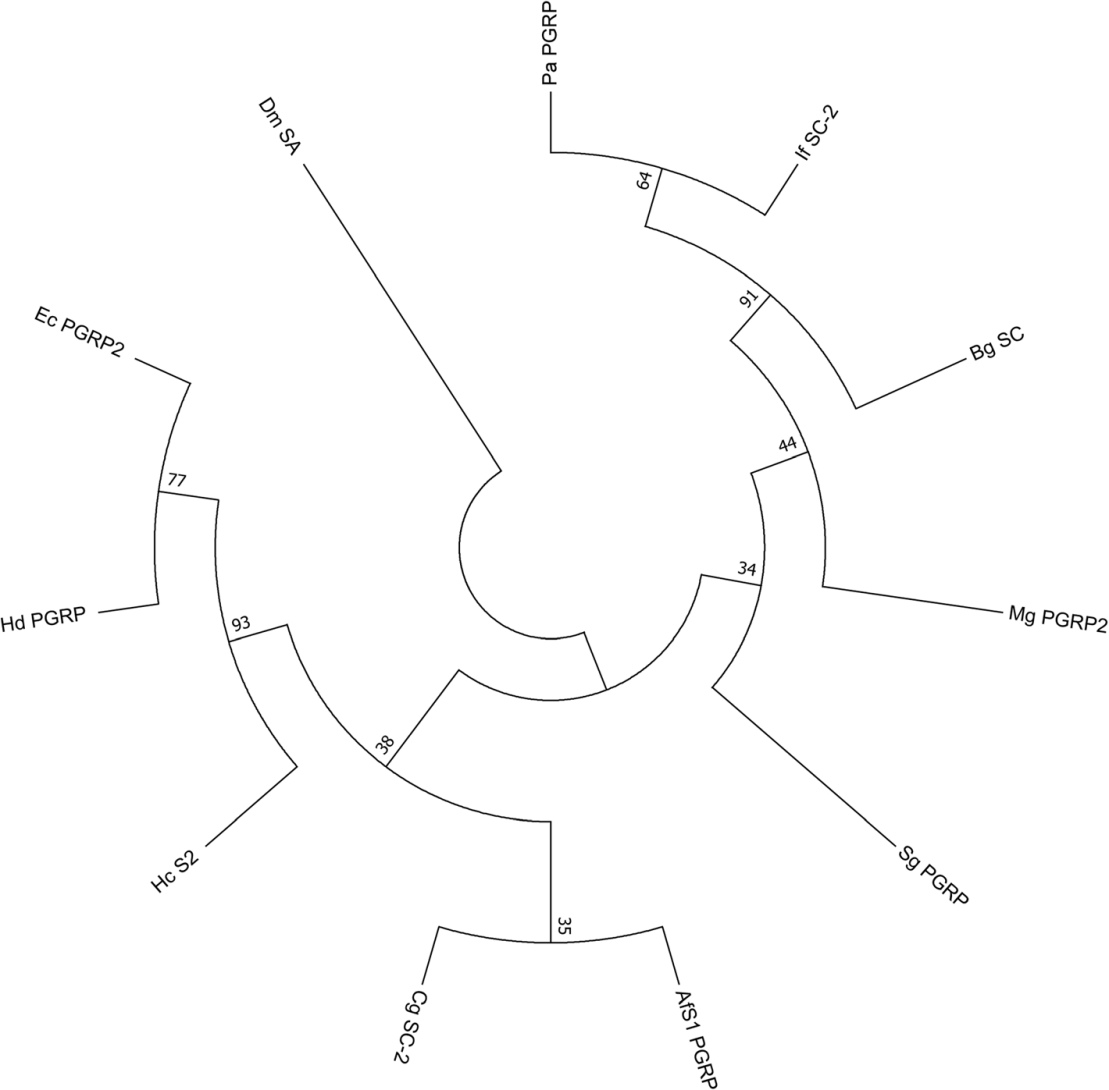
Phylogenetic analysis of If_PGRP_SC-2. The PGRP domain was considered for the analysis using the maximum likelihood method of MEGA7 program. The tree was bootstrapped (1000 replications) and the values are indicated by numbers at the nodes. The GenBank accession numbers are:: Pa_PGRP, No. JF831447.1; Bg_SC, No. XM_013209309.1; Sg_PGRP, No. JN642118.1; Hd_PGRP, No. KF554145.1; Ec_PGRP2, No. AY956812.1; Hc_S2, No. KF941201.1; AfS1_PGRP, No. AY987008.1; Cg_SC-2, No. XM_011424461.2; Mg_PGRP2, No. KP125936.1 and Dm_SA, No. AF207540.1

**Fig. 8:**
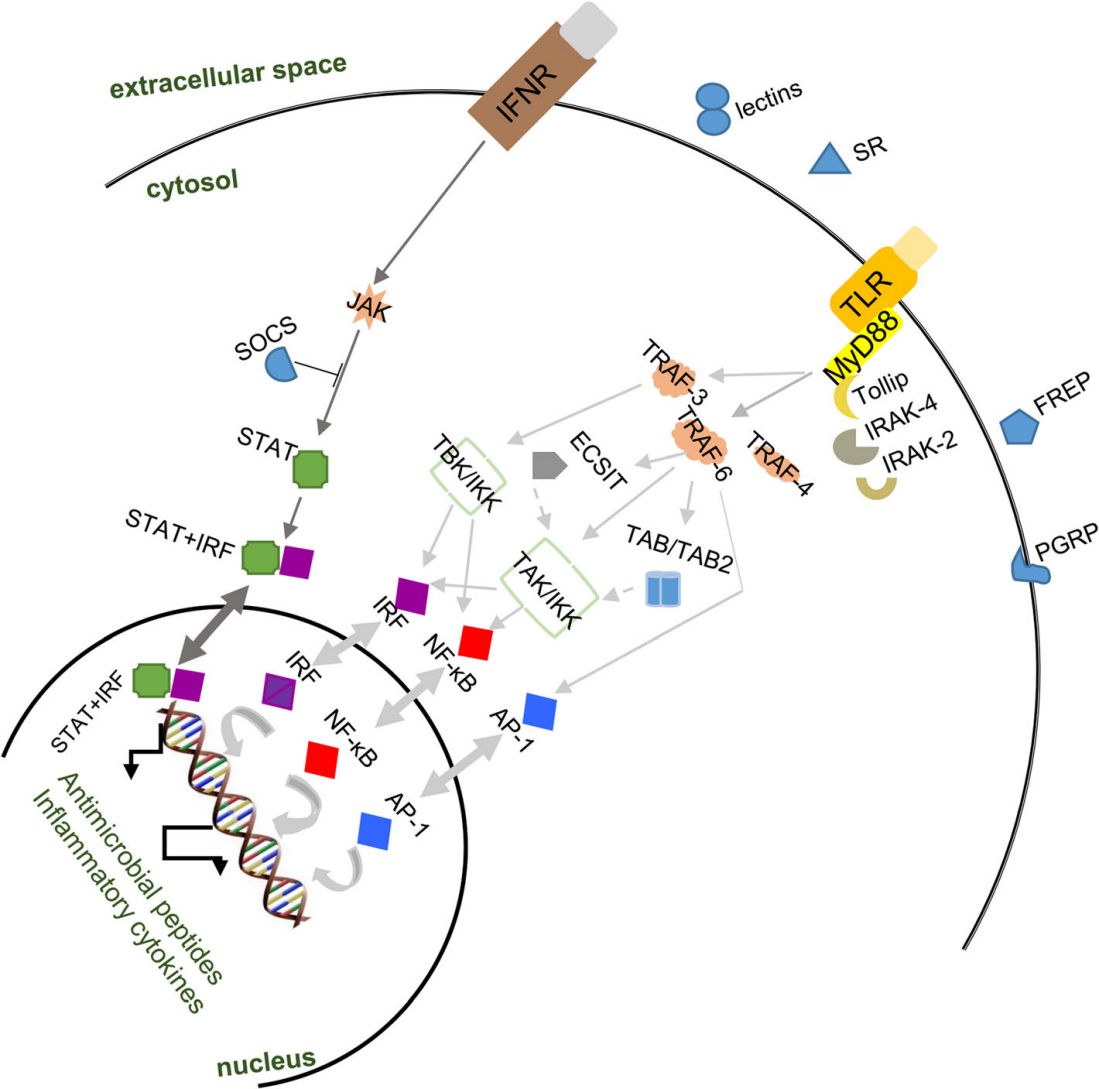
The schematic representation of TLR and JAK-STAT pathways postulated in the slug *I. fruhstorferi*. IFNR, interferon receptor; JAK, janus kinase; SOCS, suppressor of cytokine signaling; STAT, signal transducer and activator of transcription; IRF, interferon regulatory factor; SR, scavenger receptors; FREP, fibrinogen-related protein; PGRP, peptidoglycan recognition protein; IRAK, interleukin-1 receptor-associated kinase; TRAF, tumor necrosis factor receptor associated factor; ECSIT, evolutionarily conserved signaling intermediate in Toll pathway; TAB, tumor growth factor-beta-activated kinase; TBK, TANK-binding kinase; IKK, IκB kinase; TAK, transforming growth factor-beta activated kinase; NF-κB, nuclear factor kappa B; AP, activator protein

In *I. fruhstorferi* transcriptome, we located a putative PGRP-SC homolog (If_PGRP_SC-2) comprising of 519 bp ORF encoding a protein of 172 amino acid residues (Additional file 10: Figure S8). If_PGRP_SC2 showed a predicted molecular mass of 19 kDa and a theoretical isoelectric point of 6.75. In silico analysis of the protein sequence revealed the characteristic overlapping of the PGRP and N-acetylmuramoyl-L-alanine amidase domains (amidase_2 domain). Further, If_PGRP_SC2 lacked a signal peptide sequence. Multiple sequence comparisons (Additional file 11: Figure S9) revealed the conserved amidase activity motifs (Zn^2+^ − binding sites at His-40, Tyr-75, His-149, and Cys-155), and the disulphide bridges. Our results reveal that ‘If_PGRP_SC2’ like other insect PGRPs are cross-linked with a single disulphide bond. Further, the residues utilized for DAP-type PGN binding (Gly-60, Trp-61, and Arg-80) are found to be conserved. If_PGRP_SC2 protein shows three α-helices and five β-strands based on predictive secondary structure analysis (Additional file 12: Figure S10). According to the phylogenetic analysis If_PGRP_SC-2 was closely clustered with *Physella acuta* PGRP and *B. glabrata* PGRP-SC with a strong bootstrap support (Fig. 7). Further, we have deciphered the TLR signalling cascade hypothesized for *I. fruhstorferi* in Fig. 8 (detailed in the discussion section).

Among the prime endogenous ligands, all classes of HSPs have been found in the *I. fruhstorferi* transcriptome including the HSP70, HSP90, HSP60, small HSPs, hypoxia-inducible factors and stress-induced phosphoprotein. We also identified enzymes involved in reactive oxygen species (ROS) and reactive nitrogen intermediate (RNI) pathways, including the dual oxidase (DUOX), superoxide dismutase (SOD), glutathione peroxidase (GPx), Nitric oxide synthase (NOS), catalase (CAT), peroxiredoxin, glutathione synthetase and glutathione-s-transferase. Furthermore, classes of cathepsins and lysozyme were also noticed, that could participate in the phagocytic defense mechanisms in the pulmonate species. Enzymes that maintain balance at the level of ROS produced and removed includes the antioxidant enzymes screened from the *I. fruhstorferi* transcriptome such as SOD (superoxide dismutase, Cu-Zn like, Cu-like and Mn-like), CAT (catalase), and GPx-7 (glutathione peroxidase). The redox proteins screened include thioredoxin, thioredoxin-1, thioredoxin domain containing protein-3, − 5, − 9, − 12, − 15, − 16, − 17, peroxiredoxin-2, − 4, − 5, − 6, glutaredoxin-2, and − 3. Considering the conserved status of the antioxidant defense mechanisms in molluscs including the components identified in the present study, we confirm the existence of a potent defense system guarding against ROS and RNIs.

Furthermore, 22 transcripts of cathepsins belonging to B, L, F, Z, and O forms have been screened from the *I. fruhstorferi* transcriptome. Incilarin A and B showing homology to animal C-type lectins were also screened in the present study. We found transcripts of caspases, Baculoviral IAP repeat containing proteins (IAPs), apoptotic inducible factors (AIF), apoptosis regulator, apoptosis inhibitor 1, Bax, and Bcl-2 from the de novo analysis of *I. fruhstorferi* transcriptome.

### Candidate genes related to sex determination and reproduction

The sex determination genes identified from the transcriptome includes the *high mobility group (HMG) domain proteins*, transcription factor *Sox-2*, protein *MAB-21*, *Wnt*, *beta-catenin 1*, *Wilms tumor protein 1*, *GADD45*, and *armadillo* proteins (Additional file 13: Table S3). Further, we made a comparative analysis of sex-related regulatory transcripts from *Mus musculus* (mouse), *Drosophila melanogaster* (fly), *Caenorhabditis elegans* (worm), *C. hongkongensis* (oyster), and *I. fruhstorferi* (Table 8). The mammalian homologues of *Sox*, *GATA-binding protein (GATA*), *ATP-dependent helicase (ATRX)*, *Hedgehog acyltransferase (Hhat)*, *Growth arrest and DNA damage inducible protein 45 (GADD45)*, *RSpondin 1*, *Sex determining protein fem*, and *Wilms tumor protein 1 (WT1)* were detected in *Incilaria* transcriptome.

**Table 8:** Comparative analyses of sex-related genes from *Mus musculus*, *Drosophila melanogaster*, *Caenorhabditis elegans*, *Crassostrea hongkongensis* and *Incilaria fruhstorferi*

		Sex-linked genes				
Gene common name	Gene full name	Mouse	Fly	Worm	Oyster	Slug
Sox (E group)	*Sry-related HMG-box*	yes *(Sox8, Sox9, Sox10)*		yes *(sox100B)*	yes *(Sox8)*	
Sox (B1 group)		yes *(Sox3)*			yes *(Sox3)*	yes *(Sox2)*
Sox (A group)	*Sex-determining region Y*	yes *(Sry)*				yes *(Sry)*
GATA	*GATA-binding protein*	yes *(GATA4)*			yes *(GATA4)*	yes *(GATA14, GATA1, GATA10)*
MAP 3 K	*Mitogen-associated protein3 kinase*	yes *(MAP 3 K1, MAP 3 K4)*			yes *(MAP 3 K1, MAP 3 K4)*	
ATRX	*ATP-dependent helicase*	yes	yes *(dATRX)*	yes *(xnp-1)*	yes	yes
Hhat	*Hedgehog acyltransferase*	yes	yes	yes	yes	yes
Wnt4	*wingless-type family member 4*	yes	yes		yes	
β-catenin	*beta-catenin*	yes	*armadillo*		yes	yes
CYP11B	*Cytochrome P450 family 11 subfamily B*	yes				
CYP19A1	*Cytochrome P450 family 19 subfamily A1*	yes				
fgf9	*fibroblast growth factor 9*	yes				
GADD45	*Growth arrest and DNA-damage inducible protein 45*	yes			yes	yes *(GADD45 gamma, GADD45 beta)*
RSPO1	*RSpondin1*	yes				yes
Fem1/3	*Sex-determining protein fem*		yes	yes	yes	yes
LHX9	*LIM homeobox gene 9*	yes			yes	
WT1	*Wilms tumor protein 1*	yes				yes
Sf1	*Steroidogenic factor 1*	yes				
CBX-2	*Chromobox-homolog 2*	yes	yes			
EMX-2	*Empty Spiracles homeobox 2*	yes				
AMH and AMHR	*Anti-Mullerian hormone and AMH Receptor*	yes				
DMRT	*Doublesex and mab-3 related transcription factor*	yes	*dsx*	*MAB-3*		
DHH	*Desert Hedgehog*	yes	yes			
Kdm3a	*Histone demethylase*	yes	yes			
Dax1	*Dosage-sensitive Sex Reversal Adrenal Hypoplasia critical region on X-chromosome*	yes			yes	
Six	*sineoculis homeobox*	yes	yes	yes	yes	
GSDF	*Gonadal soma derived growth factor*	yes				
PDGF	*Platelet-derived growth factor*	yes *(PDRFα, PDRFβ)*				
AR	*Androgen Receptor*	yes				
FOXL2	*Forkhead box protein L2*	yes			yes	
FST	*follistatin*	yes			yes	
Era	*Estrogen receptor*	yes			yes	
Sdc	*Sex-determination and dosage compensation*	yes	yes	yes		
Tra	*Transformer*	yes	yes	yes	yes	yes
Fru	*fruitless*		yes			

Under the reproduction-related transcripts, we identified unigenes related to *sperm flagellar protein, spermatogenesis-associated protein, sperm surface protein, spermidine synthase, spermine oxidase, sperm-associated antigens, testis expressed sequences, vitellogenin*, and kinases in the present study (Additional file 13: Table S3). In the *Incilaria* transcriptome, sperm associated antigen − 1, − 6, − 7, − 16, and − 17 were noticed. The overall scope of sex-determination and reproduction related genes from the air-breathing land slug, *I. fruhstorferi* provides substantive evidence for understanding the molluscan sex-development and differentiation.

### Candidate genes related to growth and muscle development

In the present study, transcriptome characterization identified transcripts putatively related to the somatotrophic axis and muscle growth in *I. fruhstorferi*. The candidate genes related to somatotrophic axis includes *insulin-related peptide*, *epidermal growth factor receptor*, *mollusk derived growth factor*, *adenosine deaminase* and other transcription factors (Additional file 14: Table S4). Further, we identified transcripts such as *actin*, *profilin*, *tropomyosin* related to muscle growth and other transcripts such as *chitinase*, *collagen*, *apolipophorins*, *dermatopontins*, *perlucin* and *calcitonin* related to overall growth and development. Here, we have identified transcripts putatively related to *actin*, *actin-1*, *actin-2-like*, *actin-5C-like*, and *actin-related protein − 3, − 5, − 6*, and *− 8*. We also found profilin-like and profilin-4-like transcripts involved in the restructuring of the actin cytoskeleton. It is speculated that profilins could regulate actin cytoskeleton through the control of actin polymerization. In the present study, we have also identified some proteins of unknown function in muscle growth. Hence, further studies at the single-gene level are warranted for more information on the regulatory mechanisms underlying growth and development in molluscs. This information would certainly advance the knowledge of candidate growth biomarkers related to molluscan development.

## Discussion

The construction of a complete mRNA-seq library was considered necessary for the characterization of regulatory transcripts in *I. fruhstorferi*, a pulmonate slug species with restricted distribution. The slug species has not been categorized under the protective list of faunal biodiversity. This makes the slug population more susceptible towards habitat degradation and environmental perturbations. The basic idea of this study was to explore the fitness traits regulating adaptation and survival of the species in the wild. The microsatellite markers directly found on the expressed transcripts is considered useful for population diversity assessment and marker-assisted breeding. For the processing of *I. fruhstorferi* transcriptome, we used the Illumina Hi-Seq 4000 platform with paired-end reads layout. Further, we considered FastQC statistics and Cutadapt data for an initial quality assessment of Illumina sequencing data as these are the preferred choices to arrive at clean datasets (with Phred quality ≥ Q20) [12, 32, 33]. To get the non-redundant unigenes, the clean reads were assembled using the Trinity platform and clustered using the TGICL package. The unigenes were further explored for the functional annotation and microsatellite identification. The average length of unigenes obtained in this study was higher compared to the transcriptome assembly of other molluscs, including *Cristaria plicata* (737.1) [12], *Satsuma myomphala* (571.7 bp) [11], *Aegista chejuensis*/*Aegista quelpartensis* (735.4/705.6) [18] and *Pinctada maxima* (407 bp) [34].

As a measure of quality, we characterized the completeness of the assembly using BUSCO assessment tool. BUSCO employs a set of genes selected from major species clade at the OrthoDB catalogue of orthologues. We were able to capture the complete expected gene content in case of *Incilaria* transcriptome, wherein at least 91.8% were single-copy and complete BUSCO and only 3.1% missing BUSCO. Our transcriptome assembly statistics showed a lower number of complete and duplicated BUSCOs when compared with transcriptome assembly of black tiger shrimp *Penaeus monodon* [35]. Further, when considering the annotation of *I. fruhstorferi* unigenes, PANM database (Protostome database covering the protein sequences from Nematoda, Arthropoda, and Mollusca groups) was considered prominent. Most homologous sequence hits in the de novo transcriptome analysis of non-model organisms such as the pearl mollusc *C. plicata* [12], land snails *Aegista chejuensis* and *Aegista quelpartensis* [18], and the diving beetle *Cybister chinensis* [36] were observed with the homologous sequences in PANM-DB. The top-hit species distribution against the PANM-DB sequences was found biased towards completely sequenced genomes. This is the reason for over-representation of *A. californica* (19,945 protein-coding genes) and *B. glabrata* (25,539 protein-coding genes) genomic sequences. The top-hit species distribution analysis is common for the annotation of non-model species with very little or no genomic information available. Further, the lack of slug genomics data is reflected in the absence of related species sequences from the BLAST results.

The functional annotation of *I. fruhstorferi* unigenes using the InterPro conserved domain search revealed the most conspicuous zinc finger, protein-kinase, immunoglobulin-fold, and carbohydrate-binding domains. The most classical C2H2 Znfs are known to have versatile functions, including DNA (DNA-binding domains of transcription factors), RNA, and protein contacts [37]. Our results support transcriptome studies in the land snail, *Satsuma myomphala* [11] and the pearl bivalve, *C. plicata* [12], wherein the C2H2 Znfs were found to be the most conserved domain with representations of immunoglobulin-like fold domain. Protein kinase-like domain attributes critical phosphorylation functions to the intracellular protein kinases related to signal extrapolation during key metabolic, cellular, or immune processes. Immunoglobulin-like and EGF-like fold domains commonly provide interaction surfaces to other proteins via their beta-sheets [38]. Galactose-binding domains are properties of lectins and are conspicuously found in several molluscan species including the mussel *Mytilus galloprovincialis* [39], Eastern oyster *Crassostrea virginica* [40], and the Manila clam *Ruditapes phillippinarum* [41]. The KOG-based classification is also routinely used for functional descriptor analysis of newly sequenced genomes. [42]. The KOG database, first created in 1997, has gone through several updates, and currently has significant genome coverage. Our results support earlier reports of KOG classification analysis in molluscs such as in the Asian clam, *Corbicula fluminea* [43], pearl bivalve, *C. plicata* [12], and the land snail, *S. myomphala* [11]. GO annotations are based on evidences presented in the form of GO ‘evidence codes’. Generally, evidence code distribution shows the over-representation of ‘electronic’ annotations that are not genetically tested, hence may infer higher false positives. Therefore, it is to be concluded with reference to GO annotations that the interpretation of unigenes relates only to the ‘predicted’ function. The predominant evidence codes distributed for *I. fruhstorferi* unigenes were IEA (inferred from electronic annotation), and hence either homology-based and/or other experimental or sequence information. The GO classification represented in the present study showed similarities to the GO term annotations for *Crassostrea hongkongensis* [44] and *C. plicata* [12]. Again, KEGG is a unified database connecting genomic information to biological pathways and provides a foresight towards the putative enzymes in the genome of the species [45].

Repeatmasker is used to screen DNA sequences for repeating elements or low complexity regions. These include interspersed repeats, including retroelements and DNA transposons [46]. Especially, the retroelements make up a family of transposons proposed to have important roles in adaptive processes. These elements are under sufficient selective pressure following environmental stress and could be attributed to specific adaptations across the taxa. These elements are the forerunners for genetic variation and contributors to phenotypic plasticity [47, 48]. Further, the discovery of microsatellites from transcriptomics analysis of non-model species has gained momentum in the last decade due to the direct presence of these polymorphisms in the coding sequence and its transferability function. These microsatellites would represent annotated markers within genes that could be directly related to abiotic and biotic stressors [49]. Largely, the traditional methodologies of marker mining such as the enrichment of genomic DNA libraries, followed by clone sequencing has been replaced with the more reliable, high-throughput, and cost-efficient Next Generation Sequencing (NGS) based transcriptome characterization [50, 51]. SSRs and Single Nucleotide Polymorphism (SNP) markers screened from transcriptomics analysis of non-model species is currently applied at the field for genetic diversity assessments and population structure dynamics having wider applicability in conservation genomics. Consistent with our results, the dinucleotide repeats were the most abundant repeats in the transcriptomics profile of other molluscan species such as the endangered land snail, *S. myomphala* [11], endangered Neritid mollusc, *Clithon retropictus* [52], and oyster, *C. hongkongensis* [44].

At the level of molluscan transcriptome, very few reports have addressed the immune defense systems so as to make an effective adjudication of the immune surveillance mechanism against biotic and abiotic stressors. The only information on genes and pathways related to innate immune system has been reported in the endangered freshwater pearl bivalve, *C. plicata* [12], Mediterranean mussel, *Mytilus galloprovincialis* [53], and gastropod, *Rapana venosa* [54]. With the transcriptome information of the pulmonate slug, *I. fruhstorferi* now available in the public domain (https://www.ncbi.nlm.nih.gov/sra/SRX3096547[accn]), greater thrust would be laid on the functional characterization of immunity and physiology-attributed genes. As the case with molluscs, self/non-self-discrimination can be considered as the most fundamental step in invertebrate immunity. With molluscans establishing niches in diverse habitats, ecological adaptation is vital to overall sustainability of species in the wild. Pathogen recognition receptors (PRRs) show great diversity in establishing one-to-one contact with pathogen-associated molecular patterns (PAMPs) at the extracellular, membrane, or intracellular surfaces, modulating immune signaling pathways related to the humoral defense response [55, 56]. We considered the full-length ORFs of Tollip and PGRP_SC2 obtained from *Incilaria* transcriptome for a detailed understanding of the putative immune signaling cascade. Tollip is an intracellular partner for TLR signaling cascade elements. In the IL-1R pathway, Tollip blocks Interleukin-1 receptor associated kinase-1(IRAK-1) phosphorylation, thus checking the IL-1 induced signaling. Further, Tollip directly interacts with TLR2 or TLR4 to negatively regulate TLR induced signaling by a similar mechanism [57]. Tollip has been cloned and characterized from Yesso scallop *Patinopecten yessoensis* (PyTollip), [58] grouper *Epinephelus tauvina* [59], crustacean *Litopenaeus vannamei* [60], and sea cucumber *Apostichopus japonicus* [61]. The predicted ORF sequence of *I. fruhstorferi* Tollip gene (IfTollip) was smaller than PyTollip (867 bp ORF encoding 288 amino acids) [58]. Among the conserved domains represented in Tollip are the C2 domain and the CUE domain. The conserved motifs of C2 domain included the basic residues at positions 87 (Arg), 132 (Arg), and 144 (His) responsible for Phosphaditylinositol-3 phosphate (PtdIns3P) and Phosphatidylinositol-1,4 diphosphate [PtdIns (4,5) P_2_] recognition and binding [62]. The CUE domain comprised of the conserved ‘Met-Phe-Pro’ sequence responsible for ubiquitin binding [63, 64]. This is true in most cases, however, according to a previous report, Tollip structure is more unstable in primates compared to arthropod groups suggesting selective pressure at the residue-level in higher organisms [65]. Peptidoglycan Recognition Proteins (PGRPs) bind to peptidoglycan present in the cell surface of bacteria promoting lysis and/or phagocytosis of the cell [66, 67]. PGRPs (both long and short forms) have been identified in few molluscan species such as *Haliotis discus discus* [68], *Solen grandis* [69], *Argopecten irradians* [70], and *Chlamys farreri* [71]. Six short form PGRPs with conserved amidase activity have been revealed from *Crassostrea gigas* genome [72]. The lack of a signal peptide in the *I. fruhstorferi* PGRP_SC2 (If_PGRP_SC2) was in agreement to the short PGRPs screened from the EST database of deep- sea mussel, *Bathymodiolus azoricus* (Ba-PGRP 2 and Ba-PGRP 4) [73]. Further, the predicted secondary structure analysis for If_PGRP_SC2 show consistency with the three-dimensional model of BaPGRP 2 [73] and *Drosophila* PGRP-LB residues [74].

A large repertoire of PRRs including lectins, scavenger receptor (class F), Down syndrome cell-adhesion molecule (DSCAM), thioester-containing proteins (TEPs), PGRP, and fibrinogen-related proteins (FREPs) have been screened from *I. fruhstorferi* transcriptome. An expansion in the PRR repertoire is expected as large number of genome and transcriptome sequencing projects involving molluscan species have been completed [72]. However, the discovery of scavenger receptor family of PRRs has been limited with only reports from oyster *Pinctada martensii* and scallop *C. farreri* [75]. We have identified scavenger receptor class F member from *I. fruhstorferi* transcriptome. In any case, PRRs have developed an extensive network of self/non-self-discrimination in molluscs defining flexible and specific responses to microbial challenges. This provides survival advantage to the molluscs in the absence of adaptive immune mechanisms. An understanding of the TLR signaling pathway is relevant in the context of *Incilaria* species transcriptome as this would be a reference for molecular immunologists to excavate the adaptation mechanisms in the wild populations. We have suggested a predictive model for the TLR signaling pathway in *I. fruhstorferi*. The TLR signaling pathway is the most promiscuous among molluscs with genes identified and putative functions annotated in model and non-model species [76–78]. TLRs bind with PAMPs and elicit MyD88-dependent or independent signaling pathway. The MyD88 dependent pathway has been well known in molluscan immunity. In this case, TNF (tumor necrosis factor) receptor-associated factor 6 (TRAF6)/TRAF3 is recruited in the cytosol upon activation of the TLR-MyD88 signaling cascade. MyD88-dependent TLR pathway is involved in apoptosis and antimicrobial functions and have been identified in molluscs including *C. farreri*, *M. galloprovincialis*, and *Cyclina sinensis* [79, 80]. TRAF3 is proposed to mediate intranuclear signal processing through TANK-binding kinase 1 (TBK1)-mediated phosphorylation of interferon regulatory factor (IRF). IRF is translocated to the nucleus where it elicits the expression of inflammatory cytokines (type I IFN). IFNs activate the Janus activated kinase –signal transducer and activator of transcription (JAK-STAT) signaling pathway [81]. We have screened IRF1, STAT2, STAT4, STAT5 as putative transcription factors mediating JAK-STAT signaling and suppressor of cytokine signaling 5 (SOCS5) as negative regulator of the pathway. Further, TRAF6 mediated NF-kB signaling via transforming growth factor-beta activated kinase (TAK) and activator protein 1 (AP1) signaling has been proposed for *I. fruhstorferi*. A mitochondrial-like ECSIT (evolutionarily conserved signaling intermediate in Toll pathway) has also been screened that could collect signals from TRAF6 resulting in the generation of reactive oxygen species (ROS). Further, an indirect interaction may exist for ECSIT eliciting NF-kB signaling. TRAF6 mediated NF-κB signaling via tumor-growth factor beta activated kinase1/2 (TAB1/TAB2) can also be postulated from the current study. Our findings are in agreement with similar studies in Zhikong scallop *C. farreri* [82], and *S. glomerata* [14]. Furthermore, contrary to genome of oyster species such as *S. glomerata* and *C. gigas*, we were unable to screen the components of mitochondrial antiviral signaling protein (MAVS)-dependent RIG-I-like receptor (RLR) signaling pathway, indispensable for antiviral immunity in the molluscs. To sum up, besides the core elements of Toll and JAK-STAT signaling pathway, accessory components such as Tollip, IL-1 receptor-associated kinase (IRAK) were also identified in the present study, indicating the completeness of signaling pathways in *I. fruhstorferi*. Furthermore, the HSPs (preferentially HSP70 and HSP90 class) activate kinases and are regarded as negative and positive regulators of NF-kB signaling [83, 84]. In case of freshwater molluscs, *Bellamya bengalensis* and *Lamellidans marginalis* inducible NOS (iNOS) have been associated with phagocytic activity [85], while DUOX has been known to be expressed in non-phagocytic cells such as the gills in oyster, *C. gigas* and the scallop, *M. yessoensis* in response to heavy metals [86]. Earlier, we have discussed the TRAF6-ECSIT pathway for mitochondrial ROS generation through the TLR-MyD88 signaling pathway in the species. Besides roles in innate immunity ROS are also implicated in oxidative stress signaling, apoptosis, cell growth and differentiation [87]. In agreement to our results, cathepsin transcripts have also been identified from *S. glomerata* transcriptome [14] and hemocytes of mussel, *C. plicata* [88] and oyster *C. virginica* [89]. Cathepsin Z (CTSZ) is a lysosomal product and is implicated in immune reactions against bacteria such as *Vibrio parahaemolyticus*, *Listeria monocytogenes*, and bacterial cell surface carbohydrates such as LPS in disk abalone, *Haliotis discus discus* [90]. Transcripts coding for lysozyme and bactericidal permeability-increasing protein (BPI) in *I. fruhstorferi* confirms the presence of bactericidal activity as an additional means of immune defense. The full-length transcript of metallothionein gene has been studied in *I. fruhstorferi* for understanding the phylogeny of the species under the molluscan clade [91]. Considering the apoptosis cascade and in agreement to apoptosis core components deciphered for *Incilaria* species, it could be speculated that AIF could drive apoptosis in a caspase-dependent manner in the molluscan species [92]. Apaf-1 mediated apoptotic cascade seems absent in the molluscs and since apaf-1 has not been observed in any other mollusc, the apoptotic cascades might work independent of apaf-1. Among the extrinsic apoptosis pathway components, TNF-α factor in association with TNF receptor-associated factor could recruit several caspases. This lay credence to the importance of apoptotic mechanisms in *I. fruhstorferi* health and sustainability in the wild. The cataloguing of immune-related functional transcripts from the de novo assembled transcriptome of the air-breathing pulmonate *I. fruhstorferi* clearly provides a defense screen that could be exploited for research on molluscan adaptation and survivability in the wild.

Sex differentiation is indirectly influenced by environmental factors such as light, temperature, nutritional conditions, and reproductive physiology of the species. Moreover, over evolutionary periods, genetic effects of sex determination have accumulated. Transcriptome resources could be vital to direct the discovery of specific pathways of sex determination and successful reproduction strategies. Gonadal transcriptome analysis in molluscan species has identified candidate genes involved in sex-determination/differentiation and reproduction for commercial exploitation of molluscs on one hand [44, 93, 94] and informed conservation planning of the threatened and endangered species on the other hand [12]. This is the first exhaustive study towards identification of molecular mechanisms underpinning reproduction in the hermaphroditic slug species. An information on the regulatory genes associated with the reproductive biology of male and female sections of *Incilaria* species would be significant towards interpreting the population structure dynamics and conservation of germplasm. As *Incilaria* is a hermaphrodite, the spermatozoa and ova are produced in an ovotestis, and released through a single hermaphroditic duct. Hence, the species was not sexed for transcriptome characterization. The transcripts of sex-determining protein *fem-1* (feminization-1) largely populated in the *Incilaria* transcriptome were also detected in *C. plicata* [12], *Haliotis discus hannai* [95], and *P. margaritifera* transcriptome [93]. This transcript is one of the many associated with hermaphrodite phenotypes in *Caenorhabditis elegans* [96]. The Sox family of transcription factors involved in the regulation of development and differentiation of germ cells have been identified in a number of invertebrates [97]. In blood clam, *Tegillarca granosa*, Sox homologues (Sox2, Sox8, Sox9, and Sox14*)* with significant difference in gene expression between males and females have been identified [94]. In the transcriptome of *C. plicata*, the HMG-box domain family members included Sox5, Sox6, Sox9, Sox11, Sox15, and SoxB2 [12]. *Sox30,* defined to be involved in the differentiation of male gametes was found to be highly expressed in the testis of the Manila clam *R. philippinarum* [98]. Homologs of β-catenin was screened from the *I. fruhstorferi* transcriptome. β-catenin activation leads to the synthesis of female sex-determining pathway. The armadillo (ARM) repeat region in molluscs is a β-catenin ortholog reported in *C. hongkongensis*, *C. gigas*, and *C. farreri* [44, 99, 100]. Several ARM proteins (ARM-1, − 2, − 3, − 4, − 6, − 7, − 8) were discovered in *I. fruhstorferi* transcriptome. Homologs of Wilms tumor protein 1 (WT-1) identified from the current study could essentially activate the anti-mullerian hormone receptor essential for mammalian urogenital development. Homologs of WT-1 and anti-mullerian hormone were not reported from the oyster, *C. hongkongensis* [44]. Although, the homologs of vertebrate *DMRT (Doublesex and MAB-3 transcription factor)* were identified from the *C. gigas* and *C. plicata* transcriptome [12, 44], it was not evident in the *C. hongkongensis* and *Incilaria* transcriptome. Further, our results are consistent to the screening of reproduction-associated transcripts in *C. plicata* [12] and the land snail, *S. myomphala* [11]. Spermatogenesis-associated protein 6 was found highly expressed in male tissues of the abalone *H. discus hannai* playing a role in flagellar mortality [95]. Most of the transcripts found are suggested to be associated with male reproductive tissues. Sperm flagellar protein 2, sperm-associated antigen 16 and 17, spermatogenesis associated protein 1 and 4, and testis-specific serine threonine protein kinase were identified from the scallop, *Nodipecten subnodosus* by suppressive subtractive hybridization and pyrosequencing [101].

Genes related to growth and muscle development is necessary as it provides an informed decision in aquaculture breeding programs towards the selection of fitness genes for improved performance of domesticated stocks. Further with the advent of genomics technologies, gene function on expressed quantitative trait locus phenotypes has provided valuable fundamental knowledge to be applied to molecular assisted breeding programs [102]. It has also facilitated genomics approaches for improved understanding of molecular mechanisms involved in growth. Gene discovery in non-model species using the next-generation sequencing platforms has been successful in cataloguing transcripts with fundamental roles in growth and muscle development, including *actin*, *myosin*, and *tropomyosin* [103, 104]. Our results are consistent with the findings in the South African abalone species, *Haliotis midae* wherein insulin-like growth factors and insulin-like growth factor binding proteins has improved the growth pertinent to aquaculture breeding [105]. The growth factors play critical role in the development of somatotrophic axis and form the skeletal muscle in finfishes [106]. Molluscan insulin-related peptide is involved in glucose metabolism and growth as well as the regulation of germ cell proliferation and maturation [107]. The molluscan insulin-related peptide has been identified from other molluscs such as the snail, *Lymnaea stagnalis* [108], bivalve, *Patinopecten yessoensis* [109], slug, *A. californica* [110], and the garden slug, *Deroceras reticulatum* [111]. Considering the muscle growth in molluscs, *actin* gene is critical in contraction, cell signaling and establishment and management of cell junctions and has been isolated from *A. californica* [112], *Haliotis* species [113, 114], *Chlamys farreri* [115], and *Rapana venosa* [116].

## Conclusions

In this study, we assembled the transcriptome of land slug, *I. fruhstorferi*, and described candidate genes involved in immunity, sex, and growth. The assembled transcriptome was considered complete by BUSCO method. Considering that this air-breathing slug is an important node in the food web with restricted habitats, the intactness of different immune signalling modes and adaptation-related genes could provide for a compelling evidence for informed conservation planning. Furthermore, identification of microsatellites repeats/markers in the coding regions would be crucial for discovering the species in newer habitats, exploring distribution ranges and study of population diversity.

## Additional files

Additional file 1: **Table S1.** Pre-processing of raw reads obtained from *Incilaria fruhstorferi* transcriptome using Illumina Next-Generation sequencer. (DOCX 13 kb)

Additional file 2: **Figure S1.** Homology statistics of *I. fruhstorferi* unigenes against PANM DB. BLASTx annotation of the unigenes to PANM DB at an E-value threshold of 1.0E-5 was used for the statistical summary. (A) E-value distribution, (B) Identity distribution, (C) Similarity distribution, (D) Sequence hits/non-hits correlated to the length of unigenes. (TIF 771 kb)

Additional file 3: **Figure S2.** Distribution of top-hit species in PANM DB matched to *I. fruhstorferi* visceral mass unigenes using BLASTx. An E-value cutoff of 1.0E-5 was utilized for the homology matching. Quite predictably, the highest matches are observed with the molluscan model, *Aplysia californica*. (TIF 566 kb)

Additional file 4: **Figure S3.** Gene Ontology (GO) based functional mapping of *I. fruhstorferi* unigenes. (A) Venn diagram showing the distribution of unigenes to three GO function categories, viz. Biological Process, Cellular Component, and Molecular Function, (B) Number of unigenes assigned to GO terms per sequence. (DOCX 13 kb) (TIF 607 kb)

Additional file 5: **Table S2.** Classification of *Incilaria fruhstorferi* candidate genes to the innate immune signaling process. (DOCX 26 kb)

Additional file 6: **Figure S4.** Validation of the *I. fruhstorferi* transcriptome assembly and annotation using PCR-sequencing approach. (A) RT-PCR analysis of the whole-body sample using gene-specific primers. M: 100 bp DNA marker; lane-1: 207 bp Tollip gene product; lane-2: PGRP-SC2 gene product; lane-3: actin-2 gene product. (B) Clustal X2 based pairwise alignment of transcriptome-derived Tollip sequence and PCR-product sequence. (C) Clustal X2 based alignment of transcriptome-derived PGRP-SC2 member and PCR product sequence. (TIF 1682 kb)

Additional file 7: **Figure S5.** The full-length nucleotide sequence for *I. fruhstorferi* Tollip (Toll interacting protein; IfTollip). The predicted ORF with the translated protein sequence is boxed. The conserved C2 and CUE domain of Tollip protein is shown in orange and blue colors, respectively. (TIF 757 kb)

Additional file 8: **Figure S6.** Multiple sequence alignment (MSA) of the amino acid sequence of IfTollip protein with representative Tollip amino acid sequences from invertebrates and vertebrates. The alignment was conducted using Clustal X2 (version 2.0) and represented with GeneDoc. The internal and terminal gaps are represented by dashes. The highly conserved C2 and CUE domains are shown using orange and green arrows. Asterisks indicate the conserved residues in the C2 domain responsible for PtdIns3P and PtdIns (4,5) P_2_ recognition and binding. The conserved ubiquitin-binding motifs found in the CUE domain are boxed. (TIF 2504 kb)

Additional file 9: **Figure S7.** Secondary structure prediction of IfTollip using PSIPHRED (version 3.3). Cylinders in pink represent alpha helices, yellow bars represent beta strands and black lines represent coils. (TIF 384 kb)

Additional file 10: **Figure S8.** The full-length nucleotide sequence for *I. fruhstorferi* Peptidoglycan Recognition Protein SC-2 (If_PGRP_SC-2). The predicted ORF with the translated protein sequence is boxed. The conserved PGRP and overlapping amidase_2 domains are underlined. (TIF 742 kb)

Additional file 11: **Figure S9.** Multiple sequence alignment (MSA) of the amino acid sequence underlying the conserved PGRP domain of If_PGRP_SC-2 protein with representative amino acid sequences from other invertebrates. The alignment was conducted using Clustal X2 (version 2.0) and represented using graphical user interface. The black and grey regions indicate the positions of amino acid identity and similarity, respectively. The residues boxed are associated with recognition of Diaminopimelic acid-type (DAP-type) PGN. ▲: Zn^2+^ binding sites, s: cysteines predicted to form disulphide bridges. (TIF 1684 kb)

Additional file 12: **Figure S10.** Secondary structure prediction of If_PGRP_SC-2 using PSI-PRED (version 3.3). Cylinders in pink represent alpha helices, yellow bars represent beta strands and black lines represent coils. (TIF 295 kb)

Additional file 13: **Table S3.** Candidate Sex-Determination and Reproduction related genes from *I. fruhstorferi* unigenes. (DOCX 20 kb)

Additional file 14: **Table S4.** Genes of interest related to growth in the land slug, *Incilaria fruhstorferi*. (DOCX 22 kb)

## Abbreviations

AIF: Apoptosis inducing factor
AP: Activator protein
ARM: Armadillo-repeat region
ATRX: ATP-dependent helicase
BLAST: Basic local alignment search tool
BPI: Bactericidal permeability-increasing protein
BUSCO: Benchmarking universal single-copy orthologs
CAT: Catalase
COI: Cytochrome oxidase I
CTL: C-type lectins
CUE: Coupling of ubiquitin conjugation to endoplasmic reticulum degradation
DMRT: Double-sex and MAB-3 transcription factor
DSCAM: Down syndrome cell-adhesion molecule
DUOX: Dual-oxidase
ECSIT: Evolutionarily conserved signalling intermediate in Toll pathway
FREP: Fibrinogen-related proteins
GO: Gene ontology
GPx: Glutathione peroxidase
HSP: Heat shock proteins
IRAK: Interleukin-1 associated kinase
IRF: Interferon regulatory factors
JAK-STAT: Janus kinase-signal transducers and activators of transcription
KEGG: Kyoto encyclopaedia of genes and genomes
KOG: Clusters of orthologous groups
LINE: Long interspersed nuclear elements
LTR: Long-terminal repeats
MEGA: Molecular Evolutionary Genetics Analysis
MISA: Microsatellite identification tool
MyD88: Myeloid differentiation factor88
NCBI: National Centre for Biotechnology Information
NOS: Nitric oxide synthase
ORF: Open reading frame
PAMP: Pathogen-associated molecular patterns
PANM-DB: Protostome database
PCR: Polymerase chain reaction
PE: Paired-ends
PGRP: Peptidoglycan recognition protein
PRR: Pattern recognition proteins
RNI: Reactive nucleotide intermediates
ROS: Reactive oxygen species
RT: Reverse-transcriptase
SINE: Short interspersed nuclear elements
SNP: Single nucleotide polymorphism
SOCS: Suppressor of cytokine signalling
SOD: Superoxide dismutase
SR: Scavenger receptors
SRA: Sequence read archive
SSR: Simple sequence repeats
TBD: TBK1/IKKi-binding domain
TBK: TANK binding kinase
TEP: Thioester-containing proteins
TGICL: TIGR gene indices clustering tool
TLR: Toll-like receptors
TNF: Tumor-necrosis factor
TRAF: Tumor necrosis factor receptor-associated factor
UTR: Untranslated regions
WEGO: Web gene ontology annotation plot
