# Supplementary material for: Transcriptome analysis of air-breathing land slug, Incilaria fruhstorferi reveals functional insights into growth, immunity, and reproduction
Source: BMC Genomics. 2019 Feb 26;20:154. doi: 10.1186/s12864-019-5526-3 (PMC6390351; doi:10.1186/s12864-019-5526-3)
Supplement: Supplementary file 1 — Table S1. Pre-processing of raw reads obtained from Incilaria fruhstorferi transcriptome using Illumina Next-Generation sequencer. (DOCX 13 kb) [file 12864_2019_5526_MOESM1_ESM.docx]

**Additional file 1: Table S1.** Pre-processing of raw reads obtained from *Incilaria fruhstorferi* transcriptome using Illumina Next-Generation sequencer.

| Total number of raw reads | |  |  |  |  |
| --- | --- | --- | --- | --- | --- |
| - Number of sequences | | 60,721,064 |  |  |  |
| - Number of bases | | 18,337,761,328 |  |  |  |
| Total read pairs processed | | 60,721,064 |  |  |  |
| - Read 1 with adapter | | 2,755,455 | 4.5% |  |  |
| - Read 2 with adapter | | 2,910,138 | 4.8% |  |  |
| Pairs written (passing filters) | | 6,0721,064 |  |  |  |
| Total base pairs processed (bp) | | 18,337,761,328 |  |  |  |
| - Read 1 (bp) | | 9,168,880,664 |  |  |  |
| - Read 2 (bp) | | 9,168,880,664 |  |  |  |
| Total written (filtered) (bp) | | 18,231,462,024 |  |  |  |
| - Read 1 (bp) | | 9,122,668,227 |  |  |  |
| - Read 2 (bp) | | 9,108,793,797 |  |  |  |
| average length after trimming (bp) | | 300.2 |  |  |  |
| percent of reads discards (%) | | 0.6% |  |  |  |
| Program | | Cutadapt | | | |
| Adapter 1 sequence | | AGATCGGAAGAGCACACGTCTGAACTCCAGTCAC | | | |
| Adapter 2 sequence | | AGATCGGAAGAGCGTCGTGTAGGGAAAGAGTGTAGATCTCGGTGGTCGCCGTATCATT | | | |
