# Supplementary figures and images for: Transcriptome analysis of air-breathing land slug, Incilaria fruhstorferi reveals functional insights into growth, immunity, and reproduction
Source: BMC Genomics. 2019 Feb 26;20:154. doi: 10.1186/s12864-019-5526-3 (PMC6390351; doi:10.1186/s12864-019-5526-3)

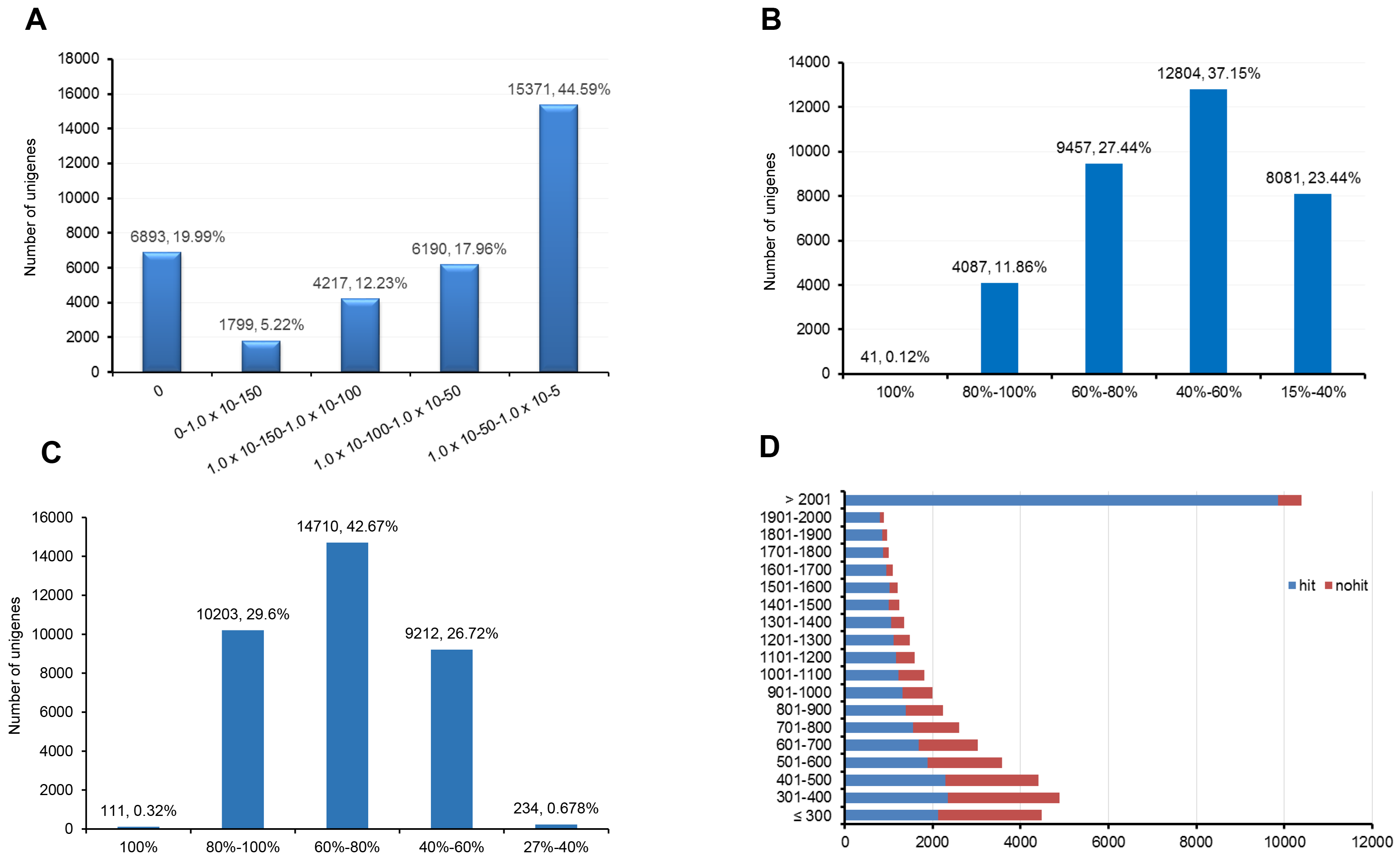

Supplement: Supplementary file 2 — Figure S1. Homology statistics of I. fruhstorferi unigenes against PANM DB. BLASTx annotation of the unigenes to PANM DB at an E-value threshold of 1.0E-5 was used for the statistical summary. (A) E-value distribution, (B) Identity distribution, (C) Similarity distribution, (D) Sequence hits/non-hits correlated to the length of unigenes. (TIF 771 kb) [file 12864_2019_5526_MOESM2_ESM.tif]

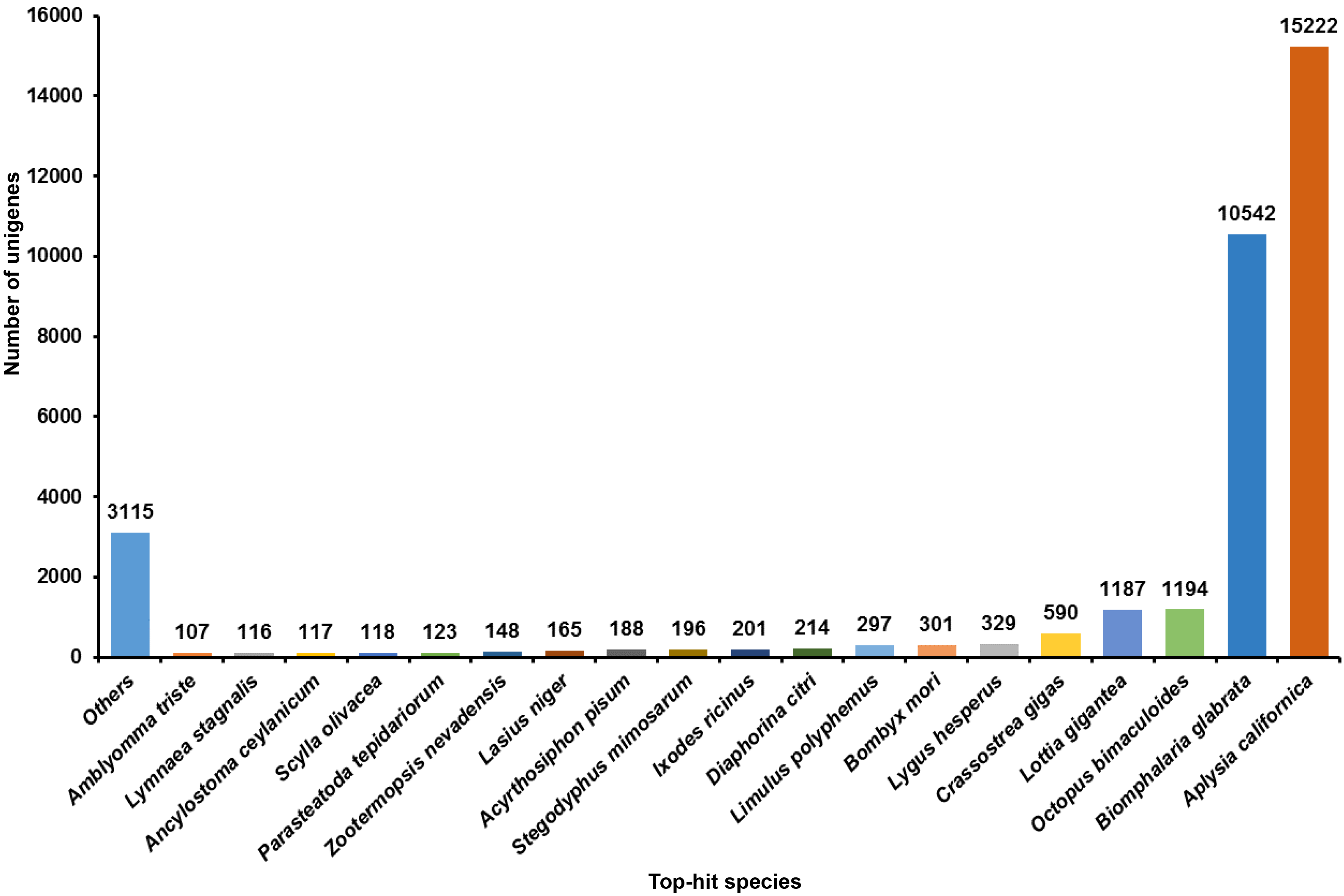

Supplement: Supplementary file 3 — Figure S2. Distribution of top-hit species in PANM DB matched to I. fruhstorferi visceral mass unigenes using BLASTx. An E-value cutoff of 1.0E-5 was utilized for the homology matching. Quite predictably, the highest matches are observed with the molluscan model, Aplysia californica. (TIF 566 kb) [file 12864_2019_5526_MOESM3_ESM.tif]

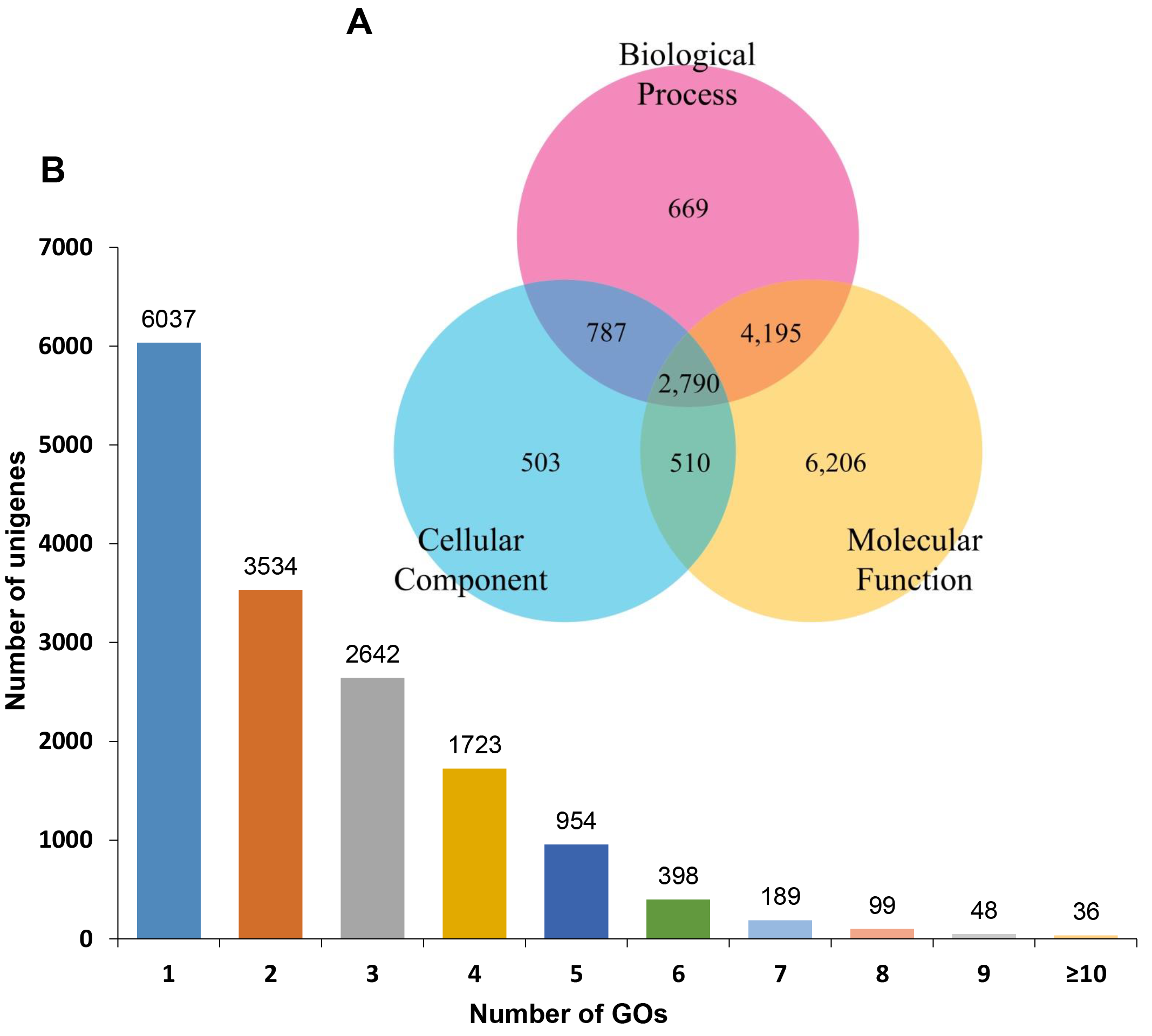

Supplement: Supplementary file 4 — Figure S3. Gene Ontology (GO) based functional mapping of I. fruhstorferi unigenes. (A) Venn diagram showing the distribution of unigenes to three GO function categories, viz. Biological Process, Cellular Component, and Molecular Function, (B) Number of unigenes assigned to GO terms per sequence. (DOCX 13 kb) (TIF 607 kb) [file 12864_2019_5526_MOESM4_ESM.tif]

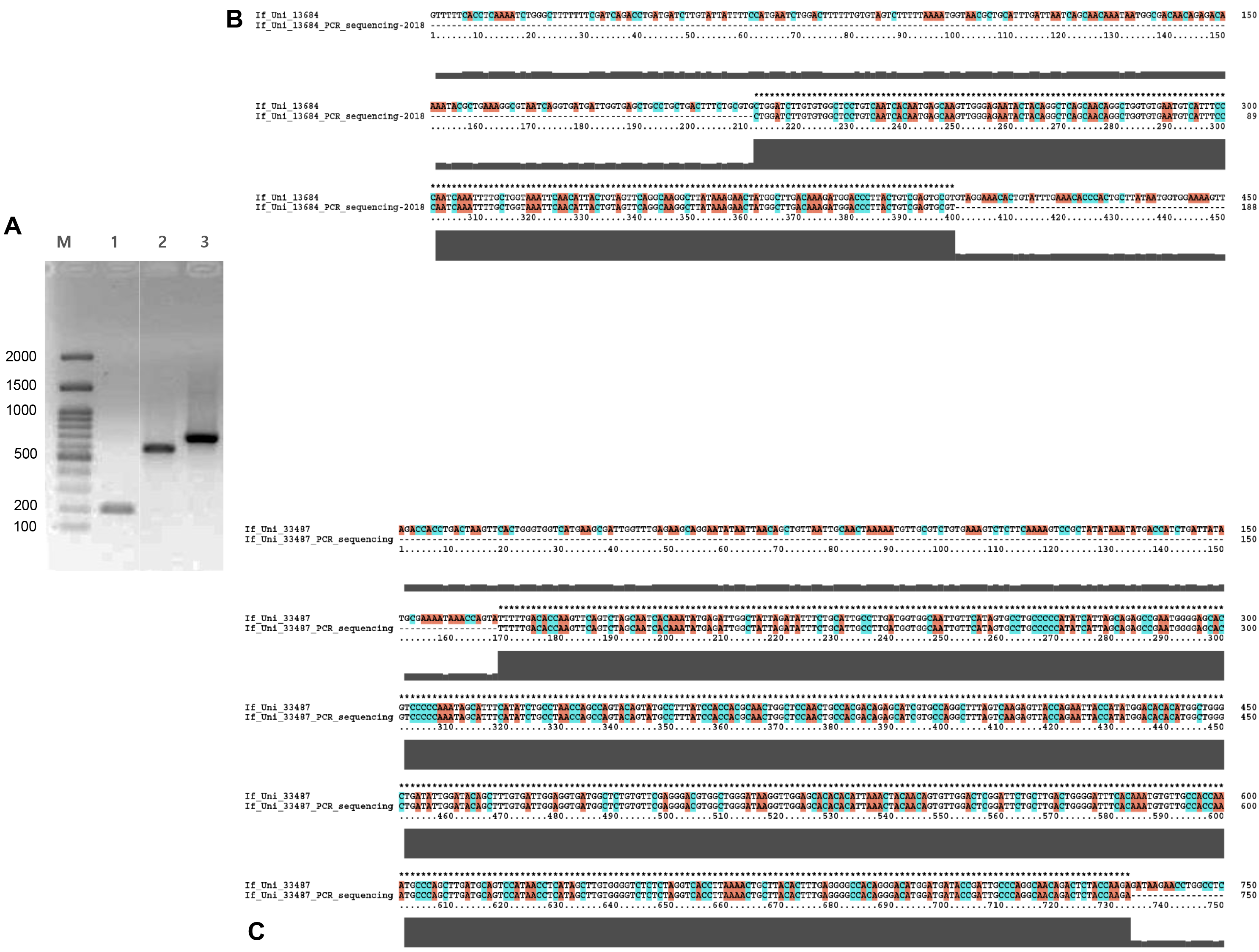

Supplement: Supplementary file 6 — Figure S4. Validation of the I. fruhstorferi transcriptome assembly and annotation using PCR-sequencing approach. (A) RT-PCR analysis of the whole-body sample using gene-specific primers. M: 100 bp DNA marker; lane-1: 207 bp Tollip gene product; lane-2: PGRP-SC2 gene product; lane-3: actin-2 gene product. (B) Clustal X2 based pairwise alignment of transcriptome-derived Tollip sequence and PCR-product sequence. (C) Clustal X2 based alignment of transcriptome-derived PGRP-SC2 member and PCR product sequence. (TIF 1682 kb) [file 12864_2019_5526_MOESM6_ESM.tif]

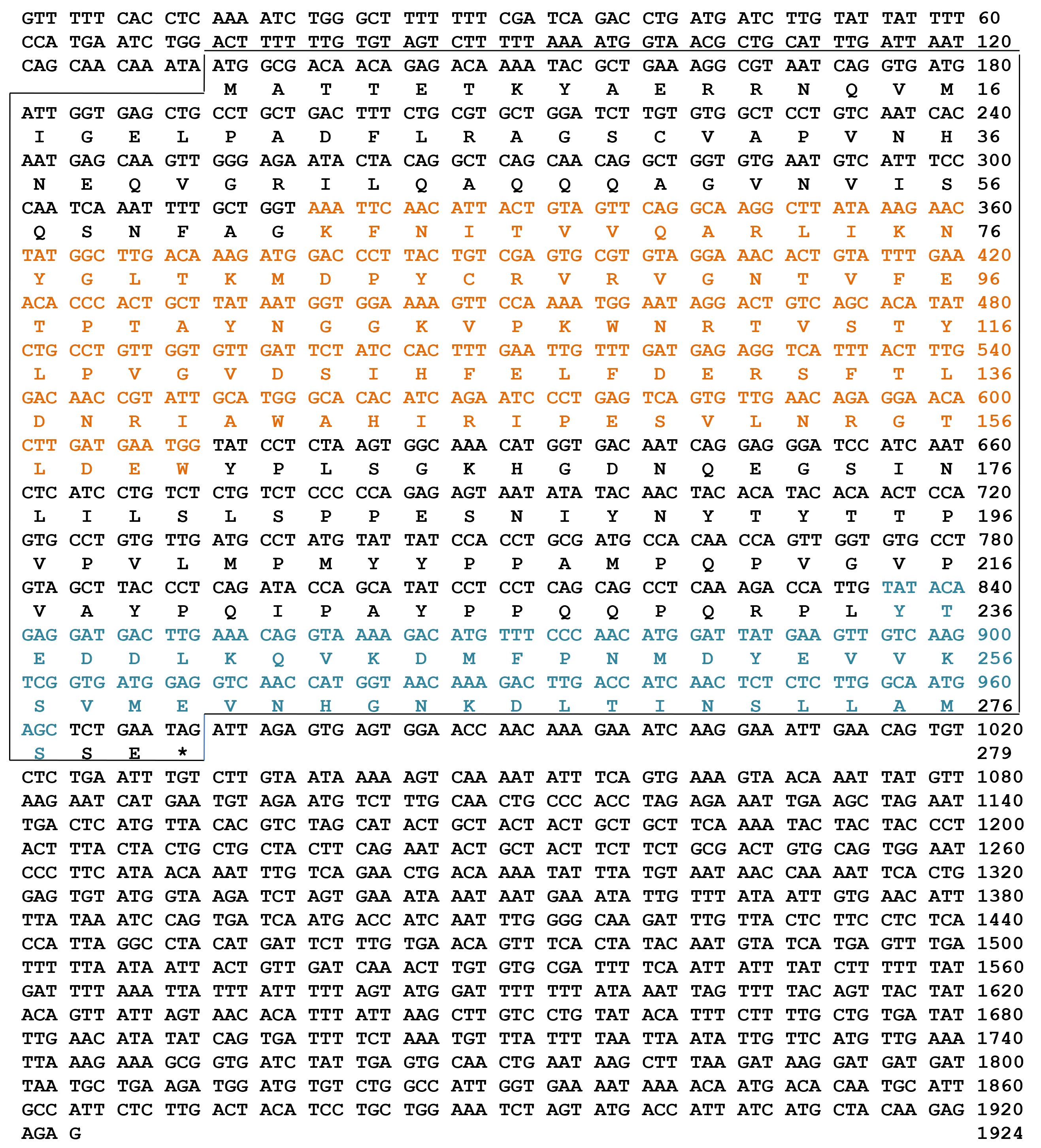

Supplement: Supplementary file 7 — Figure S5. The full-length nucleotide sequence for I. fruhstorferi Tollip (Toll interacting protein; IfTollip). The predicted ORF with the translated protein sequence is boxed. The conserved C2 and CUE domain of Tollip protein is shown in orange and blue colors, respectively. (TIF 757 kb) [file 12864_2019_5526_MOESM7_ESM.tif]

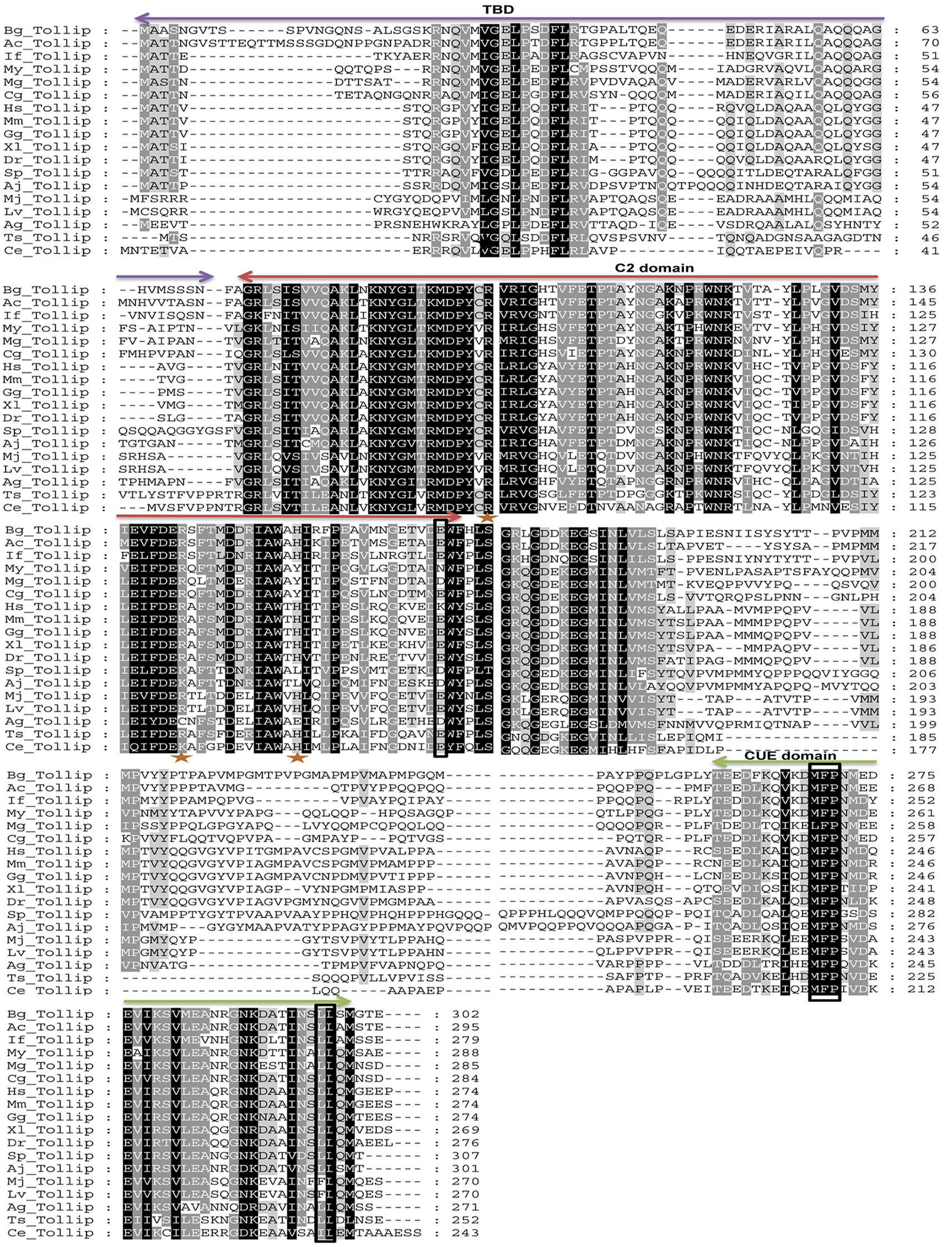

Supplement: Supplementary file 8 — Figure S6. Multiple sequence alignment (MSA) of the amino acid sequence of IfTollip protein with representative Tollip amino acid sequences from invertebrates and vertebrates. The alignment was conducted using Clustal X2 (version 2.0) and represented with GeneDoc. The internal and terminal gaps are represented by dashes. The highly conserved C2 and CUE domains are shown using orange and green arrows. Asterisks indicate the conserved residues in the C2 domain responsible for PtdIns3P and PtdIns (4,5) P2 recognition and binding. The conserved ubiquitin-binding motifs found in the CUE domain are boxed. (TIF 2504 kb) [file 12864_2019_5526_MOESM8_ESM.tif]

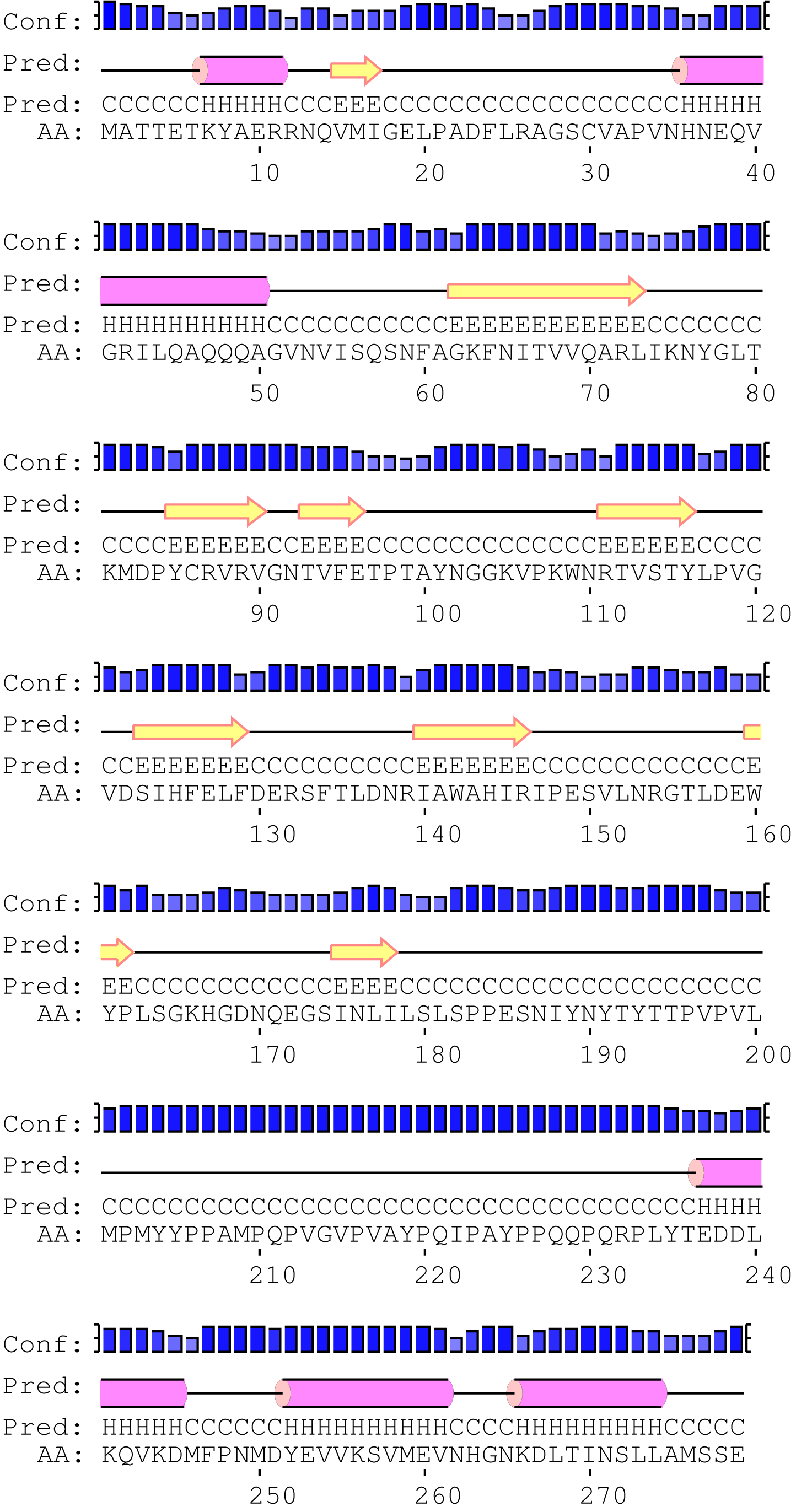

Supplement: Supplementary file 9 — Figure S7. Secondary structure prediction of IfTollip using PSIPHRED (version 3.3). Cylinders in pink represent alpha helices, yellow bars represent beta strands and black lines represent coils. (TIF 384 kb) [file 12864_2019_5526_MOESM9_ESM.tif]

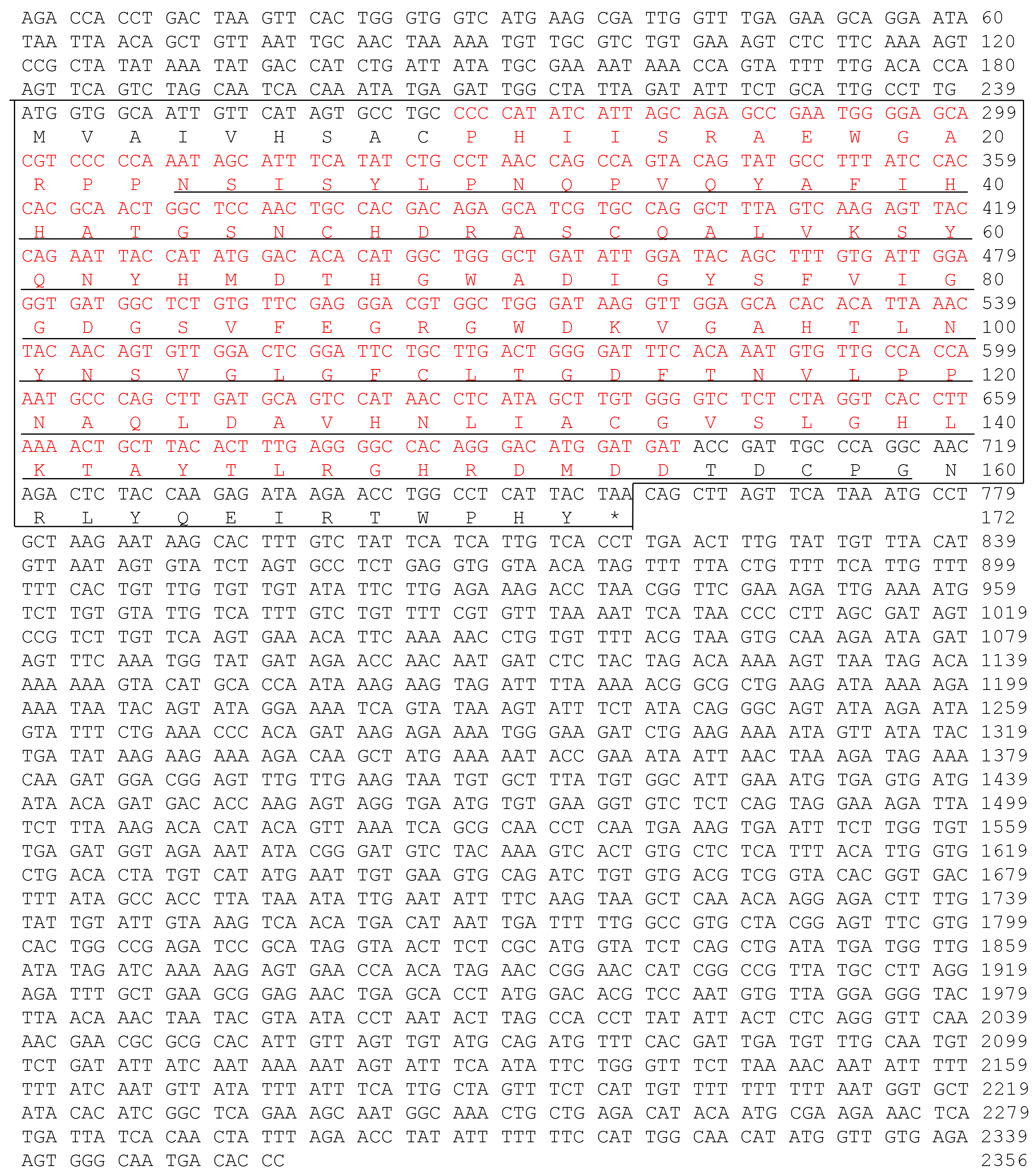

Supplement: Supplementary file 10 — Figure S8. The full-length nucleotide sequence for I. fruhstorferi Peptidoglycan Recognition Protein SC-2 (If_PGRP_SC-2). The predicted ORF with the translated protein sequence is boxed. The conserved PGRP and overlapping amidase_2 domains are underlined. (TIF 742 kb) [file 12864_2019_5526_MOESM10_ESM.tif]

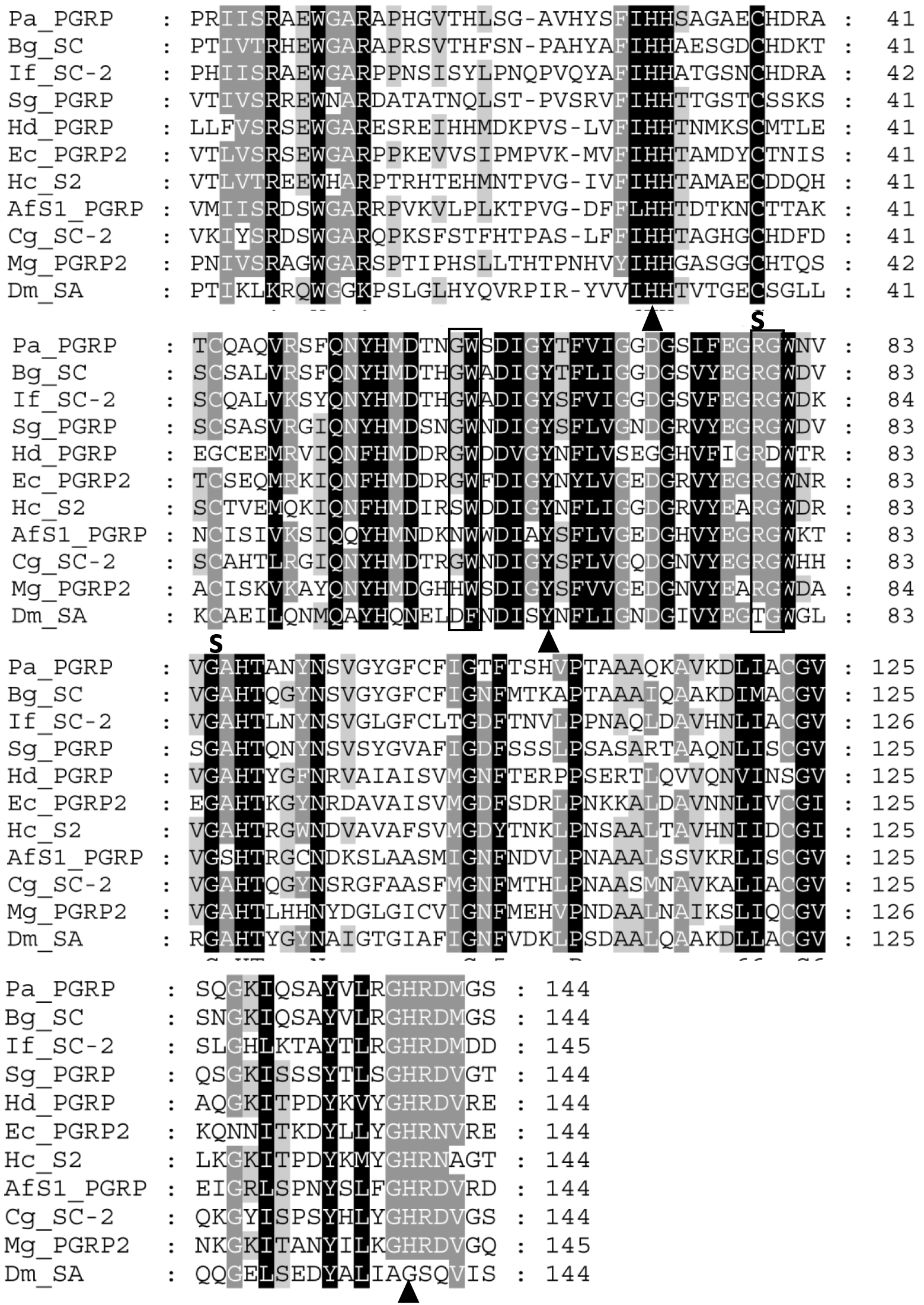

Supplement: Supplementary file 11 — Figure S9. Multiple sequence alignment (MSA) of the amino acid sequence underlying the conserved PGRP domain of If_PGRP_SC-2 protein with representative amino acid sequences from other invertebrates. The alignment was conducted using Clustal X2 (version 2.0) and represented using graphical user interface. The black and grey regions indicate the positions of amino acid identity and similarity, respectively. The residues boxed are associated with recognition of Diaminopimelic acid-type (DAP-type) PGN. ▲: Zn2+ binding sites, s: cysteines predicted to form disulphide bridges. (TIF 1684 kb) [file 12864_2019_5526_MOESM11_ESM.tif]

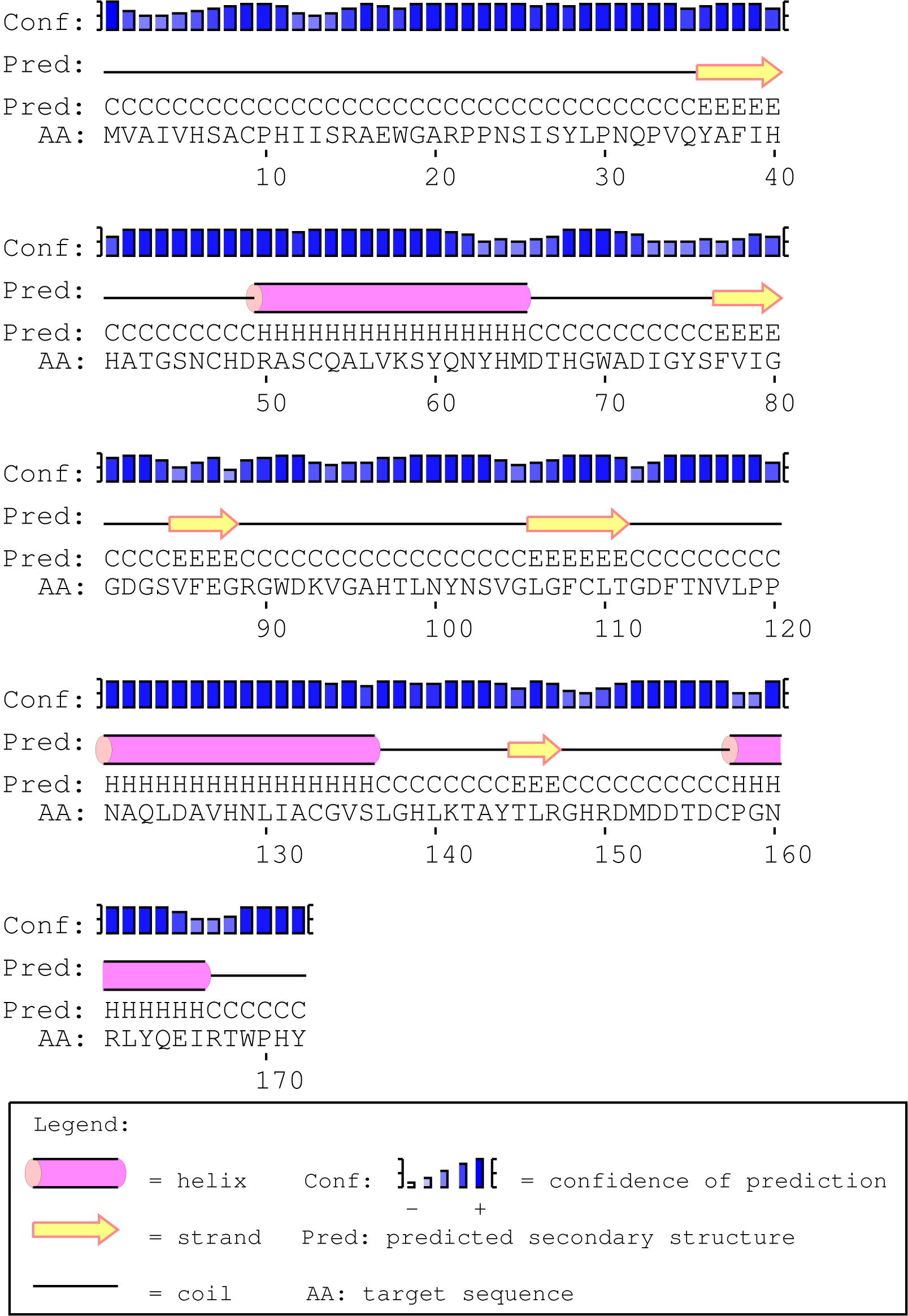

Supplement: Supplementary file 12 — Figure S10. Secondary structure prediction of If_PGRP_SC-2 using PSI-PRED (version 3.3). Cylinders in pink represent alpha helices, yellow bars represent beta strands and black lines represent coils. (TIF 295 kb) [file 12864_2019_5526_MOESM12_ESM.tif]
