# Supplementary material for: Transcriptome analysis of air-breathing land slug, Incilaria fruhstorferi reveals functional insights into growth, immunity, and reproduction
Source: BMC Genomics. 2019 Feb 26;20:154. doi: 10.1186/s12864-019-5526-3 (PMC6390351; doi:10.1186/s12864-019-5526-3)
Supplement: Supplementary file 5 — Table S2. Classification of Incilaria fruhstorferi candidate genes to the innate immune signaling process. (DOCX 26 kb) [file 12864_2019_5526_MOESM5_ESM.docx]

**Additional file 5: Table S2:** Classification of *Incilaria fruhstorferi* candidate genes to the innate immune signalling process.

| Candidate genes | Unigenes ID | | Length (bp) |
| --- | --- | --- | --- |
| PRR pathogen recognition receptor | | | |
| Immune signalling pathway | | | |
| Toll-like receptor | If_Uni_49242 | | 476 |
| Toll-like receptor 3 | If_Uni_30095, If_Uni_35302, If_Uni_36691, If_Uni_38466, If_Uni_42320, If_Uni_45580, If_Uni_46975, If_Uni_49493 | | 1671, 1240, 666, 1375, 980, 1554, 959, 1284 |
| Toll-like receptor 5 | If_Uni_49505 | | 715 |
| Toll-like receptor 13 | If_Uni_04699, If_Uni_07149, If_Uni_21888, If_Uni_35559, If_Uni_49074, If_Uni_49863 | | 300, 2611, 951, 1250, 1054, 902 |
| Tolloid-like protein 1 | If_Uni_28210, If_Uni_30172, If_Uni_30795 | | 593, 2122, 1433 |
| Tolloid-like protein 2 | If_Uni_28209, If_Uni_28211, If_Uni_31308, | | 1052, 921, 2151, |
| Protein toll-like | If_Uni_33526, If_Uni_41564 | | 2463, 3057 |
| Peptidoglycan-recognition protein SC2 | If_Uni_33487, If_Uni_44378, If_Uni_47103 | | 2356, 1091, 922 |
| C-type lectin 5 | If_Uni_32167, If_Uni_35169 | | 1099, 708 |
| C-type lectin domain family 6 member A | If_Uni_40952 | | 559 |
| C-type lectin lectoxin-Lio3-like, partial | If_Uni_42546, If_Uni_48572 | | 486, 621 |
| C-type lectin domain family 4 member E | If_Uni_45780 | | 1229 |
| I-type lectin-like protein 1 | If_Uni_07147, If_Uni_09894, If_Uni_31360 | | 5602, 3725, 2725 |
| Sialic acid binding lectin | If_Uni_35002 | | 981 |
| Chitinase-like lectin | If_Uni_35566, If_Uni_47665 | | 885, 455 |
| Techylectin-5A-like | If_Uni_01353, If_Uni_04134, If_Uni_07532, If_Uni_09220, If_Uni_21591, If_Uni_32983, If_Uni_39412 | | 1371, 1724, 286, 499, 1310, 1696, 717, |
| Plectin-like | If_Uni_13259, If_Uni_14155, If_Uni_44195 | | 5277, 4341, 1299 |
| Malectin-A-like | If_Uni_34065 | | 1009 |
| Malectin-B-like | If_Uni_41002 | | 1905 |
| Collectin-12 | If_Uni_13429, If_Uni_17218, If_Uni_34460 | | 3567, 334, 251 |
| P-selectin-like | If_Uni_47105 | | 664 |
| Endoplasmic reticulum lectin 1-like | If_Uni_27593 | | 3646 |
| Ficolin-1 | If_Uni_21266, If_Uni_28108, If_Uni_29943, If_Uni_43736, If_Uni_48703 | | 843, 2092, 1345, 289, 1802 |
| Ficolin-2 | If_Uni_21302 | | 796 |
| Macrophage mannose receptor 1 | If_Uni_10535, If_Uni_20098, If_Uni_22464, If_Uni_22560, If_Uni_24178, If_Uni_26749, If_Uni_26821, If_Uni_32322, If_Uni_35155, If_Uni_45924 | | 781, 1246, 2728, 2316, 1676, 6437, 3933, 5235, 451, 1179 |
| Galectin | If_Uni_43909 | | 290 |
| Galectin-4 | If_Uni_08465, If_Uni_08466, If_Uni_16808, If_Uni_28515, If_Uni_38412 | | 1808, 1841, 1783, 831, 4094 |
| Galectin-6 | If_Uni_17384, If_Uni_28514 | | 1193, 500 |
| Probable ATP-dependent RNA helicase DDX58 | If_Uni_26330, If_Uni_42529 | | 4654, 495 |
| Interferon-induced helicase C domain-containing protein 1 | If_Uni_40305 | | 919 |
| Thioester-containing protein | If_Uni_35099, If_Uni_37193, If_Uni_41859, If_Uni_41862, If_Uni_41886 | | 963, 1381, 4734, 4737, 4710 |
| Fibrinogen-like protein A | If_Uni_31956 | | 1710 |
| Fibrinogen-related protein 2 | If_Uni_19981 | | 2379 |
| Scavenger receptor class F member 1 | If_Uni_06239, If_Uni_06240, If_Uni_06241 | | 1790, 1497, 1525 |
| Soluble scavenger receptor cysteine-rich domain-containing protein SSC5D | If_Uni_12407 | | 767 |
| Scavenger receptor cysteine-rich domain superfamily protein | If_Uni_28816 | | 1598 |
| Down syndrome cell adhesion molecule-like protein 1 | If_Uni_25845, If_Uni_25846, If_Uni_42141 | | 1454, 1340, 1198 |
| Stabilin-2-like | If_Uni_27538, If_Uni_28510, If_Uni_28511, If_Uni_29909, If_Uni_29910, If_Uni_34499, If_Uni_36623, If_Uni_41759, If_Uni_45569 | | 899, 1708, 1562, 2950, 3088, 1192, 533, 415, 397 |
| TLR Signalling pathway | | | |
| Adaptor proteins | | | |
| Myeloid differentiation primary response protein MyD88 | If_Uni_29126, If_Uni_49700 | | 1653, 1241 |
| Tumor necrosis factor alpha-induced protein 3 | If_Uni_05305, If_Uni_23222, If_Uni_23223, If_Uni_23224, If_Uni_23225 | | 3252, 3832, 3919, 4081, 4082 |
| Sterile alpha and TIR motif-containing protein | If_Uni_42983 | | 666 |
| TRAF3-interacting protein 1 | If_Uni_17269, If_Uni_17270, If_Uni_17374 | | 469, 462, 2078 |
| TRAF-type zinc finger domain-containing protein 1 | If_Uni_30200 | | 2115 |
| MYD88 dependent pathway | | | |
| Interleukin-1 receptor-associated kinase 1 | | If_Uni_29826, If_Uni_34551, If_Uni_45053, If_Uni_45313 | 1850, 1432, 1066, 628 |
| interleukin-1 receptor-associated kinase 4 | | If_Uni_02873, If_Uni_15505, If_Uni_15506 | 1364, 2016, 2119 |
|  | |  |  |
| Toll-interacting protein-like | | If_Uni_13684, If_Uni_13685, If_Uni_47840 | 1924, 1719, 1416 |
| TNF-receptor associated factor 2-like | | If_Uni_31626 | 2485 |
| TNF-receptor associated factor 4-like | | If_Uni_34945, If_Uni_48285 | 912, 892 |
| TNF-receptor associated factor 6-like | | If_Uni_28345, If_Uni_38146 | 2366, 1313 |
| TGF-beta-activated kinase 1 and MAP3K7-binding protein 2 | | If_Uni_33233 | 3543 |
| Serine/threonine-protein kinase TBK1 | | If_Uni_05979, If_Uni_26306, If_Uni_28825, If_Uni_35452 | 3571, 3647, 3583, 608 |
| Evolutionarily conserved signalling intermediate in Toll pathway, mitochondrial-like | | If_Uni_15538, If_Uni_15539 | 1804, 903 |
| Nuclear factor NF-kappa-B p105 subunit-like | | If_Uni_43165 | 698 |
| Transcription factor AP-1-like | | If_Uni_07415, If_Uni_29479 | 2067, 1625 |
| Interferon regulatory factor 1 | | If_Uni_34231 | 1369 |
| Interferon regulatory factor 2-binding protein | | If_Uni_31407 | 3114 |
| Tyrosine-protein kinase JAK2 | | If_Uni_19633, If_Uni_38658, If_Uni_39709 | 4187, 4393, 2112 |
| Signal transducer and activator of transcription 2 | | If_Uni_33439 | 385 |
| Signal transducer and activator of transcription 4 | | If_Uni_24906 | 4115 |
| Signal transducer and activator of transcription 5B | | If_Uni_27847, If_Uni_27848, If_Uni_38940, If_Uni_48650 | 1629, 1038, 523, 372 |
| Signal transducer and activator of transcription c | | If_Uni_19642 | 428 |
| Mitogen-activated protein kinase 1 | | If_Uni_06628, If_Uni_22689, If_Uni_37327 | 1825, 1678, 1713 |
| Mitogen-activated protein kinase 7 | | If_Uni_35999, If_Uni_44957 | 1368, 509 |
| Mitogen-activated protein kinase 14 | | If_Uni_47839, If_Uni_48270, If_Uni_49942 | 1406, 1616, 1390 |
| Mitogen-activated protein kinase 15 | | If_Uni_28827, If_Uni_28829 | 2104, 2417 |
| Dual specificity mitogen-activated protein kinase kinase 1 | | If_Uni_19703 | 2435 |
| Dual specificity mitogen-activated protein kinase kinase 4 | | If_Uni_35161 | 821 |
| Dual specificity mitogen-activated protein kinase kinase 5 | | If_Uni_47186 | 1419 |
| Dual specificity mitogen-activated protein kinase kinase 6 | | If_Uni_16379, If_Uni_16977 | 2594, 2058 |
| Dual specificity mitogen-activated protein kinase kinase 7 | | If_Uni_18770, If_Uni_31869 | 2720, 1738 |
| Mitogen-activated protein kinase kinase kinase 1 | | If_Uni_13325 | 12283 |
| Mitogen-activated protein kinase kinase kinase 2 | | If_Uni_30597, If_Uni_39139, If_Uni_48469 | 1237, 410, 645 |
| Mitogen-activated protein kinase kinase kinase 4 | | If_Uni_40353, If_Uni_43883 | 328, 466 |
| Mitogen-activated protein kinase kinase kinase 7 | | If_Uni_33344, If_Uni_33700, If_Uni_37621 | 1930, 2236, 3186 |
| Mitogen-activated protein kinase kinase kinase 15 | | If_Uni_30407, If_Uni_31326 | 1568, 2830 |
| Mitogen-activated protein kinase kinase kinase kinase 5 | | If_Uni_42647 | 323 |
| Protein pellino-like | | If_Uni_42057 | 1009 |
| Suppressor of cytokine signalling 5 | | If_Uni_38955 | 894 |
| NF-kappa-B inhibitor-like protein 1 | | If_Uni_30762 | 2987 |
| NF-kappa-B inhibitor zeta-like | | If_Uni_35246 | 836 |
| NF-kappa-B inhibitor cactus-like | | If_Uni_40218, If_Uni_43678, If_Uni_48190 | 1929, 2741, 1633 |
| NF-kappa-B essential modulator-like | | If_Uni_46407 | 724 |
| NF-kappa-B inhibitor-interacting Ras-like protein 1-like | | If_Uni_09292, If_Uni_09293, If_Uni_09294, If_Uni_09295, If_Uni_09296, If_Uni_10491 | 1938, 1512, 1383, 2650, 2142, 1844 |
| NF-kappa-B inhibitor alpha-like | | If_Uni_28034, If_Uni_28035 | 1744, 1188 |
| C-Jun-amino-terminal kinase-interacting protein 4 | | If_Uni_25956, If_Uni_31124, If_Uni_32451, If_Uni_47123, If_Uni_49644 | 5135, 1793, 1334, 863, 539 |
| Serpin B6-like | | If_Uni_20795 | 1981 |
| ENDOGENOUS LIGANDS | |  |  |
| Heat shock protein 70 B2-like | | If_Uni_09052, If_Uni_09053, If_Uni_23324, If_Uni_23325, If_Uni_23326 | 1926, 3457, 2146, 2550, 2635 |
| Heat shock 70 kDa protein cognate 4 | | If_Uni_23327, If_Uni_23328, If_Uni_33435, If_Uni_43426 | 2338, 2197, 2643, 2749 |
| Heat shock 70 kDa protein 4 | | If_Uni_42286 | 4176 |
| Heat shock 70 kDa protein 10 | | If_Uni_43360 | 285 |
| Heat shock 70 kDa protein 13 | | If_Uni_36140, If_Uni_43584 | 879, 609 |
| Heat shock 70 kDa protein 14 | | If_Uni_22068, If_Uni_22069, If_Uni_22070 | 1324, 4415, 4727 |
| Hsp70-binding protein 1 | | If_Uni_41490 | 2947 |
| hsp90 co-chaperone Cdc37 | | If_Uni_09168, If_Uni_09169 | 1730, 2992 |
| 60 kDa heat shock protein | | If_Uni_26767, If_Uni_26768, If_Uni_26769 | 2475, 2527, 1910 |
| 10 kDa heat shock protein | | If_Uni_46878 | 715 |
| Heat shock protein 83-like (p) | | If_Uni_15132, If_Uni_38595, If_Uni_41620 | 3002, 804, 2946 |
| Heat shock protein 75 kDa, mitochondrial-like (p) | | If_Uni_21652, If_Uni_33869 | 3766, 5097 |
| Heat shock protein beta-11-like (p) | | If_Uni_30326 | 1314 |
| Small heat shock protein p36-like (p) | | If_Uni_40345 | 421 |
| Heat shock protein Hsp-12.2-like (p) | | If_Uni_44603, If_Uni_48992 | 1045, 1231 |
| Stress-induced-phosphoprotein 1 | | If_Uni_40412 | 1871 |
| HSPB1-associated protein 1 | | If_Uni_21147 | 2224 |
| Hypoxia-inducible factor 1-alpha | | If_Uni_36282, If_Uni_41890, If_Uni_47693 | 916, 394, 609 |
| prolyl 3-hydroxylase 1-like | | If_Uni_48015 | 571 |
| prolyl 3-hydroxylase 2-like | | If_Uni_39229 | 356 |
| prolyl 4-hydroxylase subunit alpha-1 | | If_Uni_12213, If_Uni_38051 | 1862, 1208 |
| prolyl 4-hydroxylase subunit alpha-2 | | If_Uni_00017, If_Uni_07383, If_Uni_08618, If_Uni_10898, If_Uni_30123, If_Uni_30124, If_Uni_33135, If_Uni_33454, If_Uni_37205, If_Uni_43067, If_Uni_45220, If_Uni_46192 | 3086, 1031, 1765, 1888, 988, 1134, 2130, 1404, 1874, 380, 651, 997 |
| CREB binding protein | | If_Uni_26021, If_Uni_26022, If_Uni_32356, If_Uni_37019, If_Uni_47182 | 6508, 6207, 1822, 604, 708 |
| TAF5-like RNA polymerase II p300/CBP-associated factor-associated factor 65 kDa subunit 5L-like | | If_Uni_31113 | 2342 |
| TAF6-like RNA polymerase II p300/CBP-associated factor-associated factor 65 kDa subunit 6L-like | | If_Uni_39535 | 351 |
| IMMUNE EFFECTORS | |  |  |
| Superoxide dismutase [Cu-Zn]-like | | If_Uni_08752, If_Uni_38009, If_Uni_09408 | 2011, 1031, 1752 |
| Superoxide dismutase [Mn] | | If_Uni_17990, If_Uni_17991 | 1348, 1642 |
| Copper chaperone for superoxide dismutase | | If_Uni_14598 | 1837 |
| Catalase | | If_Uni_24966, If_Uni_25499, If_Uni_42978 | 3306, 3408, 2436 |
| Glutathione peroxidase-like | | If_Uni_33478 | 2645 |
| Glutathione peroxidase 7 | | If_Uni_19757 | 1902 |
| Epididymal secretory glutathione peroxidase-like | | If_Uni_12329, If_Uni_32414 | 1104, 1085 |
| Nitric oxide synthase-interacting protein-like | | If_Uni_28782 | 2343 |
| Thioredoxin | | If_Uni_13662, If_Uni_33725, If_Uni_46042, If_Uni_46154, If_Uni_49023 | 829, 923, 564, 759, 1224 |
| Thioredoxin-1 | | If_Uni_38714 | 1832 |
| Thioredoxin-T | | If_Uni_45275, If_Uni_46609 | 1124, 771 |
| Thioredoxin domain-containing protein | | If_Uni_00620, If_Uni_08894 | 1742, 1891 |
| Thioredoxin domain-containing protein 3 | | If_Uni_26513, If_Uni_26514, If_Uni_26515 | 2659, 2312, 1931 |
| Thioredoxin domain-containing protein 5 | | If_Uni_11641, If_Uni_36206 | 2630, 645 |
| Thioredoxin domain-containing protein 9 | | If_Uni_29217, If_Uni_29218 | 1596, 1680 |
| Thioredoxin domain-containing protein 12 | | If_Uni_07430, If_Uni_07431, If_Uni_22571, If_Uni_22572, If_Uni_22756, If_Uni_23228, If_Uni_23229, If_Uni_23230, If_Uni_49697 | 1036, 1107, 1718, 2560, 3674, 2796, 3376, 2794, 1534 |
| Thioredoxin domain-containing protein 15 | | If_Uni_30385 | 2268 |
| Thioredoxin domain-containing protein 16 | | If_Uni_41325, If_Uni_41523, If_Uni_42128 | 343, 483, 810 |
| Thioredoxin domain-containing protein 17 | | If_Uni_14782 | 927 |
| Thioredoxin reductase 1 | | If_Uni_25440, If_Uni_25441, If_Uni_28618 | 2628, 4131, 1842 |
| Peroxiredoxin-2 | | If_Uni_33319 | 1911 |
| Peroxiredoxin-4 | | If_Uni_38410 | 1569 |
| Peroxiredoxin-5 | | If_Uni_03921, If_Uni_14477, If_Uni_17596, If_Uni_30885, If_Uni_44687 | 1131, 1233, 1949, 668, 1160 |
| Peroxiredoxin-6 | | If_Uni_19430, If_Uni_49162 | 1383, 1183 |
| Glutaredoxin | | If_Uni_02646 | 1361 |
| Glutaredoxin-2 | | If_Uni_10076, If_Uni_19190, If_Uni_20095, If_Uni_33874 | 1869, 1995, 2831, 2419 |
| Glutaredoxin-3 | | If_Uni_12645 | 2014 |
| Glutaredoxin-related protein 5, mitochondrial-like | | If_Uni_20226 | 1220 |
| Glutathione S-transferase-like | | If_Uni_47696, If_Uni_08892 | 458, 1294 |
| Glutathione S-transferase 1-like | | If_Uni_44857, If_Uni_44990, If_Uni_46987 | 1042, 1113, 843 |
| Glutathione S-transferase P 1-like | | If_Uni_45724 | 1948 |
| Glutathione S-transferase 2-like | | If_Uni_07822, If_Uni_47066 | 1025, 894 |
| Glutathione S-transferase 3 | | If_Uni_13763, If_Uni_18830, If_Uni_07635, If_Uni_07636 | 921, 825, 546, 733 |
| Glutathione S-transferase 4 | | If_Uni_07926, If_Uni_20246, If_Uni_29805 | 1244, 946, 1511 |
| Glutathione S-transferase 7 | | If_Uni_10789, If_Uni_12087, If_Uni_14555, If_Uni_14556, If_Uni_30238, If_Uni_30239,If_Uni_40300, If_Uni_44270, If_Uni_45604, If_Uni_47672 | 1243, 1906, 2389, 1783, 816, 914, 1817, 1010, 1081, 1883 |
| Glutathione S-transferase mu class protein | | If_Uni_49404 | 1154 |
| Glutathione S-transferase Mu 1-like | | If_Uni_44064 | 2880 |
| Glutathione S-transferase C-terminal domain-containing protein | | If_Uni_07943, If_Uni_11417, If_Uni_11418 | 2647, 2447, 1442 |
| Glutathione S-transferase U19 | | If_Uni_08893 | 4090 |
| Glutathione S-transferase omega-1 | | If_Uni_22103, If_Uni_22104, If_Uni_26463, If_Uni_26464, If_Uni_29916, If_Uni_29917 | 1080, 1115, 302, 501, 1081, 938 |
| Glutathione S-transferase kappa 1 | | If_Uni_13355 | 2494 |
| Glutathione S-transferase theta-2 | | If_Uni_13888 | 1147 |
| Glutathione S-transferase alpha-5 | | If_Uni_15401, If_Uni_15849, If_Uni_28697 | 1042, 741, 1304 |
| Glutathione S-transferase DHAR1, mitochondrial-like | | If_Uni_48302 | 458 |
| Glutathione synthetase | | If_Uni_13363 | 2663 |
| Glutamate–cysteine ligase regulatory subunit | | If_Uni_06947, If_Uni_26307 | 1555, 1563 |
| Glutamate–cysteine ligase catalytic subunit | | If_Uni_27263 | 2947 |
| Glutathione reductase, mitochondrial-like | | If_Uni_26054, If_Uni_26055 | 2035, 680 |
| Cytochrome b-245 light chain-like | | If_Uni_21508, If_Uni_21509, If_Uni_22238 | 694, 839, 657 |
| Cytochrome b-245 heavy chain-like | | If_Uni_37041 | 1883 |
| Dual oxidase-like | | If_Uni_48406 | 590 |
| Dual oxidase 2-like | | If_Uni_09765, If_Uni_09766, If_Uni_10604 | 12197, 6303, 6382 |
| Cathepsin B-like | | If_Uni_02852, If_Uni_02964, If_Uni_11107, If_Uni_16194, If_Uni_21319, If_Uni_21320, If_Uni_21624, If_Uni_21638, | 1579, 1162, 1873, 1750, 864, 379, 2486, 1231 |
| Cathepsin L1-like | | If_Uni_20284, If_Uni_24834, If_Uni_24835, If_Uni_33266 | 3376, 1322, 1322, 452 |
| Cathepsin L-like | | If_Uni_26178, If_Uni_26179, If_Uni_29936, If_Uni_33352, If_Uni_33267, If_Uni_45916 | 2791, 1233, 1148, 1917, 722, 810 |
| Cathepsin Z-like | | If_Uni_39242 | 2198 |
| Cathepsin F-like | | If_Uni_43950, If_Uni_49279 | 2658, 369 |
| Cathepsin O-like | | If_Uni_48281 | 1694 |
| G-type lysozyme | | If_Uni_18862 | 1392 |
| Lysozyme-like | | If_Uni_48479 | 1300 |
| ANTIMICROBIAL PEPTIDES | |  |  |
| Bactericidal permeability-increasing protein | | If_Uni_36732 | 977 |
|  | |  |  |
| CYTOKINES AND CYTOKINE RECEPTORS | |  |  |
| Macrophage migration inhibitory factor-like | | If_Uni_37905 | 2290 |
| OTHERS | |  |  |
| Septin-2 | | If_Uni_34008 | 1726 |
| Septin-4 | | If_Uni_45935, If_Uni_46306, If_Uni_46978 | 971, 1305, 817 |
| Septin-7 | | If_Uni_26579, If_Uni_26580, If_Uni_29414, If_Uni_30162, If_Uni_33622, If_Uni_41517, If_Uni_41518 | 1062, 1926, 2072, 2112, 2962, 2912, 2992 |
| Septin-11 | | If_Uni_43943 | 2359 |
| Apolipophorins | | If_Uni_16384, If_Uni_16385, If_Uni_42973 | 10902, 9355, 797 |
| Apolipoprotein B-100-like | | If_Uni_32444, If_Uni_39217 | 1138, 860 |
| CD63 antigen | | If_Uni_12430, If_Uni_22061 | 1740, 4974 |
| Chitinase-like protein | | If_Uni_00986, If_Uni_01277, If_Uni_11422, If_Uni_03370, If_Uni_13899, If_Uni_14759, If_Uni_17041, If_Uni_43048, If_Uni_17756, If_Uni_23341, If_Uni_24979, If_Uni_26112, If_Uni_26113, If_Uni_26114, If_Uni_26948, If_Uni_29418, If_Uni_33736, If_Uni_33737, If_Uni_43587, If_Uni_48457, If_Uni_49703, If_Uni_25075, If_Uni_10101, If_Uni_10102 | 2006, 1386, 3944, 1870, 2868, 2458, 2966, 2346, 1533, 1021, 2145, 1700, 1736, 1879, 3559, 1827, 2368, 2328, 279, 1779, 680, 3732, 524, 442 |
| Chitinase 3 | | If_Uni_03027, If_Uni_08588, If_Uni_15144, If_Uni_16700, If_Uni_16701, If_Uni_16702, If_Uni_16703, If_Uni_16704, If_Uni_16705, If_Uni_18667, If_Uni_25012, If_Uni_25013, If_Uni_25015, If_Uni_25016, If_Uni_37541, If_Uni_08589, If_Uni_05746, If_Uni_06062, If_Uni_24166, If_Uni_24167, If_Uni_24170, If_Uni_24978, If_Uni_18668, If_Uni_18669, If_Uni_25014, If_Uni_25017, If_Uni_25854, If_Uni_25855, If_Uni_17502, If_Uni_24836, If_Uni_24837, If_Uni_28758, If_Uni_47731 | 2077, 3237, 2160, 2120, 1891, 1884, 2433, 2465, 1998, 2981, 2962, 3420, 2792, 2946, 3438, 3024, 3175, 390, 3636, 6795, 3021, 1184, 2768, 2371, 2962, 2777, 3002, 3071, 602, 2353, 2293, 1014, 1394 |
| Endochitinase A | | If_Uni_17663, If_Uni_37443 | 262, 2037 |
| Endochitinase-like | | If_Uni_23342, If_Uni_23343, If_Uni_41456 | 1731, 2476, 937 |
| Chitinase domain-containing protein 1 | | If_Uni_35132 | 1519 |
| Calreticulin | | If_Uni_07705, If_Uni_07706, If_Uni_39458 | 2672, 2664, 1974 |
| Metallothionein | | If_Uni_31828, If_Uni_49751 | 310, 414 |
| Metallothionein 20-I | | If_Uni_14651, If_Uni_45145, If_Uni_49717 | 2318, 1109, 1333 |
| Incilarin B | | If_Uni_01826, If_Uni_32527, If_Uni_33021 | 404, 521, 834 |
| Incilarin A | | If_Uni_40354 | 500 |
| APOPTOSIS | |  |  |
| TNF-alpha factor | | If_Uni_06312 | 501 |
| LPS-induced TNF-alpha factor | | If_Uni_11035, If_Uni_30478, If_Uni_38623, If_Uni_41451 | 649, 758, 3542, 3464 |
| TNF receptor-associated factor 2-like | | If_Uni_31626 | 2485 |
| TNF receptor-associated factor 4-like | | If_Uni_34945, If_Uni_48285 | 912, 892 |
| TNF receptor-associated factor 6-like | | If_Uni_28345, If_Uni_38146 | 2366, 1313 |
| Apoptosis-inducing factor 1, mitochondrial | | If_Uni_31080, If_Uni_31081 | 2157, 2081 |
| Apoptosis-inducing factor 3-like | | If_Uni_09491, If_Uni_09492, If_Uni_09493, If_Uni_09494, If_Uni_09495 | 4921, 4699, 2797, 2912, 4700 |
| Apoptosis regulator BAX-like | | If_Uni_07100, If_Uni_37212 | 1053, 929 |
| Caspase activity and apoptosis inhibitor 1 | | If_Uni_04198, If_Uni_20151 | 2968, 3551 |
| Caspase-3-like | | If_Uni_10106, If_Uni_28319, If_Uni_34315, If_Uni_44745, If_Uni_47763 | 2363, 1416, 1471, 1315, 882 |
| Caspase-6-like | | If_Uni_19000 | 1362 |
| caspase-7-like | | If_Uni_32107, If_Uni_43906 | 1442, 413 |
| caspase-8-like | | If_Uni_26608 | 2422 |
| Caspase-9-like | | If_Uni_15234, If_Uni_39864 | 1903, 1779 |
| Baculoviral IAP repeat-containing protein 2 | | If_Uni_26290, If_Uni_43433, If_Uni_45424 | 3810, 420, 1019 |
| Baculoviral IAP repeat-containing protein 3 | | If_Uni_34549, If_Uni_34564, If_Uni_38842, If_Uni_41504, If_Uni_44592, If_Uni_46291 | 1643, 1641, 393, 239, 591, 722 |
| Baculoviral IAP repeat-containing protein 5 | | If_Uni_37526 | 982 |
| Baculoviral IAP repeat-containing protein 6 | | If_Uni_35610, If_Uni_41596, If_Uni_47637 | 516, 661, 1036 |
| Baculoviral IAP repeat-containing protein 7 | | If_Uni_07425, If_Uni_19448, If_Uni_30377, If_Uni_30378 | 631, 1689, 2275, 1757 |
| Interferon alpha-inducible protein 27 | | If_Uni_07528, If_Uni_14229, If_Uni_33895, If_Uni_40246, If_Uni_40621 | 1765, 1487, 2693, 1946, 2445 |
| Bcl-2-related protein A1 (p) | | If_Uni_08517, If_Uni_08523, If_Uni_17381, If_Uni_42837 | 2291, 5199, 1205, 287 |
| Bcl-2-like protein 2 (p) | | If_Uni_17362, If_Uni_40799 | 1681, 1432 |
| Bcl-2-related ovarian killer protein homolog B (p) | | If_Uni_29810, If_Uni_29811 | 1862, 1456 |
| BCL2/adenovirus E1B 19 kDa protein-interacting protein 3(p) | | If_Uni_32662, If_Uni_37591 | 1272, 1399 |
