# Supplementary material for: Transcriptome analysis of air-breathing land slug, Incilaria fruhstorferi reveals functional insights into growth, immunity, and reproduction
Source: BMC Genomics. 2019 Feb 26;20:154. doi: 10.1186/s12864-019-5526-3 (PMC6390351; doi:10.1186/s12864-019-5526-3)
Supplement: Supplementary file 13 — Table S3. Candidate Sex-Determination and Reproduction related genes from I. fruhstorferi unigenes. (DOCX 20 kb) [file 12864_2019_5526_MOESM13_ESM.docx]

**Additional file 13: Table S3:** Candidate Sex-Determination and Reproduction related genes from *I. fruhstorferi* unigenes.

| Candidate genes | Unigenes ID | Length (bp) |
| --- | --- | --- |
| Sex determination and differentiation related | | |
| Bkm protein, partial | If_Uni_32848 | 582 |
| WD repeat and HMG-box DNA-binding protein 1 | If_Uni_38092, If_Uni_43552, If_Uni_45660 | 1959, 328, 572 |
| High mobility group protein HMGI-C | If_Uni_04066, If_Uni_18207, If_Uni_19532, If_Uni_19533 | 1554, 1650, 1538, 1507 |
| HMG box-containing protein 4 | If_Uni_33076 | 2167 |
| Transcription factor Sox-2 | If_Uni_29379 | 1869 |
| Protein mab-21 | If_Uni_40510, If_Uni_16265, If_Uni_29770, If_Uni_48742 | 354, 2570, 2100, 536 |
| GATA zinc finger domain-containing protein 14 | If_Uni_27207, If_Uni_37511, If_Uni_41878, If_Uni_32259, If_Uni_36121 | 2799, 3530, 245, 1942, 517 |
| GATA zinc finger domain-containing protein 1 | If_Uni_32769 | 3842 |
| GATA-binding factor 2 | If_Uni_33870, If_Uni_43253 | 731, 339 |
| GATA-binding factor A | If_Uni_36825 | 849 |
| GATA zinc finger domain-containing protein 10 | If_Uni_48787 | 803 |
| MAP3K7-binding protein 1 | If_Uni_17697 | 1891 |
| MAP3K12-binding inhibitory protein 1 | If_Uni_19605, If_Uni_19606, If_Uni_19607, If_Uni_19608, If_Uni_19609, If_Uni_19628 | 1046, 1059, 1022, 1045, 1024, 1798 |
| MAP3K7-binding protein 2 | If_Uni_33233 | 3543 |
| MAP7 domain-containing protein 1 | If_Uni_05762, If_Uni_27096, If_Uni_49226 | 3568, 2475, 943 |
| MAP/microtubule affinity-regulating kinase 3 | If_Uni_10668, If_Uni_11752, If_Uni_28516 | 4864, 4742, 1759 |
| MAPK/MAK/MRK overlapping kinase | If_Uni_13482 | 2158 |
| MAP kinase-interacting serine/threonine-protein kinase 1 | If_Uni_32415, If_Uni_30206, If_Uni_38946 | 1069, 1076, 2036 |
| MAP kinase-activated protein kinase 2 | If_Uni_34044, If_Uni_38654 | 1448, 1877 |
| JNK1/MAPK8-associated membrane protein | If_Uni_34572 | 701 |
| MAP kinase-activating death domain protein | If_Uni_39356 | 520 |
| Transcriptional regulator ATRX | If_Uni_06018, , If_Uni_17932, If_Uni_26690, If_Uni_36083, If_Uni_38408, If_Uni_43097, If_Uni_45402, If_Uni_47888, If_Uni_11246 | 521, 2146, 6216, 916, 6377, 341, 1045, 528, 1337 |
| HHAT (Hedgehog acyltransferase) | If_Uni_28282, If_Uni_28283, If_Uni_28284, If_Uni_35562, If_Uni_40027, If_Uni_40967, If_Uni_45744, If_Uni_47180 | 2563, 2780, 2670, 494, 554, 921, 818, 867 |
| Wnt-1 | If_Uni_26347 | 1194 |
| beta-catenin 1 | If_Uni_11446 | 1897 |
| catenin beta | If_Uni_11892 | 4447 |
| cyp6 | If_Uni_32736, If_Uni_32737, If_Uni_45065 | 1188, 1231, 1181 |
| CYP95 | If_Uni_38389 | 1010 |
| Cytochrome P450 CYP44 | If_Uni_33928, If_Uni_33929, If_Uni_39536(p) | 2310, 2391, 512 |
| Wilms tumor protein 1 | If_Uni_30347 | 1165 |
| Fibroblast growth factor receptor 4 | If_Uni_04028, If_Uni_18914, If_Uni_18915 | 2594, 3142, 2557 |
| Fibroblast growth factor 18 | If_Uni_28940, If_Uni_47461 | 2633, 674 |
| fibroblast growth factor receptor 1 | If_Uni_34011, If_Uni_42307 | 1391, 447 |
| Fibroblast growth factor receptor | If_Uni_46026 | 493 |
| FGFR1 oncogene partner | If_Uni_02734, If_Uni_40079, If_Uni_40342, If_Uni_40400, If_Uni_40493 | 761, 1793, 1829, 1814, 1778 |
| GADD45 gamma | If_Uni_12848 | 3581 |
| GADD45 beta | If_Uni_46781 | 1060 |
| Growth arrest and DNA damage-inducible proteins-interacting protein 1 | If_Uni_19527, If_Uni_44790 | 1272, 965 |
| Probable E3 ubiquitin-protein ligase HERC4 | If_Uni_21856, If_Uni_21857, If_Uni_34001, If_Uni_42518 | 4048, 4174, 5665, 445 |
| Probable E3 ubiquitin-protein ligase HERC1 | If_Uni_26496, If_Uni_26497, If_Uni_29658, If_Uni_29659, If_Uni_39736, If_Uni_43224, If_Uni_46389 | 7095, 7810, 2518, 2607, 616, 382, 842 |
| E3 ubiquitin-protein ligase HERC2 | If_Uni_26552, If_Uni_26553, If_Uni_30702, If_Uni_33927 | 595, 7304, 2054, 1040 |
| Spondin-1 | If_Uni_05763, If_Uni_38348(p) | 360, 1888 |
| SCO-spondin-like | If_Uni_24270, If_Uni_24271, If_Uni_24273, If_Uni_26659, If_Uni_27061, If_Uni_27062, If_Uni_29417, If_Uni_31661, If_Uni_41552 | 3415, 4608, 474, 2344, 3753, 5096, 4936, 659, 316 |
| RPE-spondin | If_Uni_33837 | 3117 |
| Armadillo protein 1 | If_Uni_48460 | 1582 |
| Armadillo protein 2 | If_Uni_28876, If_Uni_28877 | 2031, 3112 |
| Armadillo protein 3 | If_Uni_13253, If_Uni_41419 | 4927, 3541 |
| Armadillo protein 4 | If_Uni_28376 | 3736 |
| Armadillo protein 6 | If_Uni_18357, If_Uni_18358, If_Uni_18359 | 1944, 1833, 659 |
| Armadillo protein 7 | If_Uni_15411, If_Uni_15412 | 1211, 1305 |
| Armadillo protein 8 | If_Uni_31330, If_Uni_31362 | 2694, 2718 |
| Reproduction related | | |
| Deadpan | If_Uni_00719 | 285 |
| Dual specificity testis-specific protein kinase 2 | If_Uni_30720, If_Uni_30721 | 2527, 1428 |
| Major royal jelly protein 1 | If_Uni_20006, If_Uni_20008, If_Uni_20009, If_Uni_20010, If_Uni_26692, If_Uni_26693, If_Uni_30602, If_Uni_47064, If_Uni_48739 | 761, 3519, 3041, 1789, 1910, 2165, 1301, 906, 1225 |
| Major royal jelly protein 2 | If_Uni_48314 | 1713 |
| Motile sperm domain-containing protein 2 | If_Uni_14761, If_Uni_23035, If_Uni_23036, If_Uni_23037 | 2741, 2926, 1910, 2228 |
| Nuclear autoantigenic sperm protein | If_Uni_23989, If_Uni_44396 | 3360, 1017 |
| Oocyte zinc finger protein | If_Uni_30920, If_Uni_36290, If_Uni_38224, If_Uni_43738 | 1734, 683, 1717, 1036 |
| Prostatic spermine-binding protein | If_Uni_19292, If_Uni_20725 | 1832, 3038 |
| Protein fem-1 | If_Uni_00131, If_Uni_00283, If_Uni_00486, If_Uni_00501, If_Uni_00654, If_Uni_01136, If_Uni_01448, If_Uni_01475, If_Uni_01478, If_Uni_01583, If_Uni_01875, If_Uni_01891, If_Uni_02033, If_Uni_02189, If_Uni_02232, If_Uni_02236, If_Uni_02487, If_Uni_02505, If_Uni_02515, If_Uni_02891, If_Uni_02892, If_Uni_03001, If_Uni_03660, If_Uni_03665, If_Uni_03668, If_Uni_03685, If_Uni_03832, If_Uni_03833, If_Uni_03834, If_Uni_03949, If_Uni_03950, If_Uni_04043, If_Uni_04097, If_Uni_04106, If_Uni_04117, If_Uni_04131, If_Uni_04200, If_Uni_04300, If_Uni_04316, If_Uni_04323, If_Uni_04393, If_Uni_04419, If_Uni_04427, If_Uni_04552, If_Uni_04553, If_Uni_04556, If_Uni_04564, If_Uni_04659, If_Uni_04745, If_Uni_04807, If_Uni_05067, If_Uni_05080, If_Uni_05263, If_Uni_06885, If_Uni_06933, If_Uni_07101, If_Uni_07338, If_Uni_09012, If_Uni_09110, If_Uni_09116, If_Uni_09322, If_Uni_09445, If_Uni_09653, If_Uni_09826, If_Uni_09914, If_Uni_10131, If_Uni_10628, If_Uni_10673, If_Uni_11312, If_Uni_11372, If_Uni_11638, If_Uni_12340, If_Uni_12930, If_Uni_12996, If_Uni_13076, If_Uni_13105, If_Uni_13216, If_Uni_13289, If_Uni_13534, If_Uni_13896, If_Uni_13917, If_Uni_14564, If_Uni_14746, If_Uni_15269, If_Uni_16046, If_Uni_17489, If_Uni_17537, If_Uni_17557, If_Uni_17641, If_Uni_17682, If_Uni_17689, If_Uni_17777, If_Uni_17791, If_Uni_17825, If_Uni_17854, If_Uni_17980, If_Uni_18051, If_Uni_18083, If_Uni_18129, If_Uni_18251, If_Uni_18301, If_Uni_18480, If_Uni_18607, If_Uni_18608, If_Uni_18609, If_Uni_18705, If_Uni_18706, If_Uni_18850, If_Uni_19057, If_Uni_19058, If_Uni_19062, If_Uni_19063, If_Uni_19067, If_Uni_19068, If_Uni_19069, If_Uni_19094, If_Uni_19379, If_Uni_19528, If_Uni_19871, If_Uni_19928, If_Uni_19939, If_Uni_19956, If_Uni_19970, If_Uni_20003, If_Uni_20334, If_Uni_20336, If_Uni_20513, If_Uni_20828, If_Uni_21006, If_Uni_21254, If_Uni_21375, If_Uni_21486, If_Uni_21779, If_Uni_21843, If_Uni_21844, If_Uni_21852, If_Uni_21947, If_Uni_22312, If_Uni_22555, If_Uni_22673, If_Uni_22697, If_Uni_22757, If_Uni_23088, If_Uni_23120, If_Uni_24421, If_Uni_24422, If_Uni_34393, If_Uni_35723, If_Uni_36655, If_Uni_39677, If_Uni_42176, If_Uni_42184, If_Uni_42802, If_Uni_43145, If_Uni_43226, If_Uni_44989, If_Uni_48159, If_Uni_49399, If_Uni_13746, If_Uni_18777, If_Uni_21663 | 276, 347, 425, 296, 289, 276, 245, 245, 317, 238, 330, 267, 245, 225, 336, 231, 317, 245, 347, 303, 253, 286, 275, 320, 288, 294, 325, 226, 334, 255, 263, 290, 275, 235, 267, 226, 251, 235, 259, 409, 319, 244, 251, 253, 270, 270, 252, 228, 311, 242, 358, 402, 256, 416, 362, 274, 788, 2622, 1769, 332, 384 , 530, 272, 6929, 559, 272, 939, 1652, 576, 658, 418, 320, 1622, 480, 227, 461, 410, 494, 1121, 294, 2028, 1350, 292, 575, 1375, 920, 340, 525, 2050, 864, 722, 363, 2183, 1038, 645, 359, 1322, 491, 436, 1154, 413, 460, 360, 388, 574, 334, 728, 410, 2849, 425, 402, 479, 314, 302, 678, 628, 664, 663, 862, 1365, 1476, 1257, 244, 646, 1552, 2631, 337, 372, 409, 574, 599, 530, 462, 530, 1009, 1689, 321, 354, 260, 287, 1613, 583, 816, 1691, 577, 236, 522, 288, 373, 393, 276, 261, 273, 569, 316, 540, 2657, 1107, 462 |
| Phosphorylase b kinase gamma catalytic chain, liver/testis | If_Uni_47808, If_Uni_49721 | 1457, 1400 |
| Sex-determining region Y protein | If_Uni_29886, If_Uni_29887,If_Uni_33274, If_Uni_39261 | 1016, 308, 307, 276 |
| Sex muscle abnormal protein 5 | If_Uni_44400 | 1124 |
| Sex hormone-binding globulin | If_Uni_47590 | 1458 |
| Sperm flagellar protein 1 | If_Uni_16240, If_Uni_16241 | 2287, 2175 |
| Sperm flagellar protein 2 | If_Uni_26320, If_Uni_26321, If_Uni_26322, If_Uni_34699, If_Uni_48338 | 5027, 4740, 1997, 900, 1570 |
| Spermatogenesis-associated serine-rich protein 1 | If_Uni_45418 | 1125 |
| Spermatogenesis-associated serine-rich protein 2 | If_Uni_15888 | 2955 |
| Spermatogenesis-associated protein 4 | If_Uni_27644 | 1864 |
| Spermatogenesis-associated protein 5 | If_Uni_20265, If_Uni_20483, If_Uni_37155, If_Uni_37229, If_Uni_31028, If_Uni_31029 | 1062, 1264, 1677, 1158, 2220, 2291 |
| Spermatogenesis associated protein 6 | If_Uni_27271, If_Uni_27272 | 1850, 1655 |
| Spermatogenesis-associated protein 7 | If_Uni_25828, If_Uni_25831, If_Uni_34844, If_Uni_25829, If_Uni_25830 | 2747, 2686, 786, 2767, 2819 |
| Spermatogenesis-associated protein 13 | If_Uni_13915, If_Uni_32059 | 2700, 1796 |
| Spermatogenesis-associated protein 20 | If_Uni_17309, If_Uni_48892 | 2878, 915 |
| Spermatogenesis-associated protein 22 | If_Uni_33213 | 1834 |
| Spermatogenesis-associated protein 24 | If_Uni_32185, If_Uni_48930 | 1365, 1197 |
| Spermatogenesis-defective protein 39 | If_Uni_06253, If_Uni_29450, If_Uni_40253 | 1536, 2373, 1757 |
| Sperm mitochondrial-associated cysteine-rich protein | If_Uni_27065 | 363 |
| sperm protein, partial | If_Uni_29952 | 888 |
| Sperm surface protein Sp17 | If_Uni_31479, If_Uni_31480 | 1003, 935 |
| Spermidine spermine n -acetyltransferase-like protein 1 | If_Uni_08425 | 922 |
| Spermidine synthase | If_Uni_28919, If_Uni_28920, If_Uni_38346 | 1797, 1767, 1058 |
| Spermine oxidase | If_Uni_14233, If_Uni_14234, If_Uni_14235, If_Uni_29154, If_Uni_29155, If_Uni_22643, If_Uni_23214, If_Uni_25662, If_Uni_25663, If_Uni_25664 | 3556, 3470, 3501, 3360, 2999, 2828, 2628, 3992, 3947, 3191 |
| Spermatozoon-associated protein kinase | If_Uni_15095, If_Uni_15882 | 1320, 2102 |
| Spermatogenesis-associated serine-rich protein 2 | If_Uni_15888 | 2955 |
| Sperm-tail PG-rich repeat-containing protein 2 | If_Uni_31713, If_Uni_06399 | 2039, 2263 |
| Sperm-associated antigen 1 | If_Uni_34162, If_Uni_38202, If_Uni_41985 | 3790, 3186, 4401 |
| Sperm-associated antigen 6 | If_Uni_16302 | 3460 |
| Sperm-associated antigen 7 | If_Uni_45775, If_Uni_46354 | 1001, 1346 |
| Sperm-associated antigen 16 | If_Uni_09674, If_Uni_11318, If_Uni_11319 | 1198, 1085, 2113 |
| Sperm-associated antigen 17 | If_Uni_26525 | 7816 |
| Testis-expressed sequence 2 protein | If_Uni_25971, If_Uni_25972, If_Uni_35550, If_Uni_41761 | 4046, 3144, 794, 3066 |
| Testis-expressed sequence 9 protein | If_Uni_20222, If_Uni_20223 | 1204, 1272 |
| Testis-expressed sequence 10 protein | If_Uni_20841, If_Uni_20842, If_Uni_20843 | 1211, 2781, 3189 |
| Testis-expressed sequence 11 protein | If_Uni_41841, | 360, 874 |
| Testis-expressed sequence 26 protein | If_Uni_16515, If_Uni_18909 | 1129, 2041 |
| Testis-expressed sequence 36 protein | If_Uni_38286 | 943 |
| Testis-expressed sequence 264 protein | If_Uni_30569 | 1418 |
| Testis-specific serine/threonine-protein kinase 2 | If_Uni_49266 | 1130 |
| Testis-specific serine/threonine-protein kinase3 | If_Uni_28983, If_Uni_31802 | 2103, 1805 |
| T-complex-associated testis-expressed protein 1 | If_Uni_33348, If_Uni_33349 | 3639, 1932 |
| Testis, prostate and placenta-expressed protein | If_Uni_41038, If_Uni_45296, If_Uni_45765 | 1832, 1090, 874 |
| Vitellogenin | If_Uni_31231 , If_Uni_31232 , If_Uni_34730, If_Uni_41030, | 1091, 1098, 734, 1975 |
| Vitelline envelope zona pellucida domain 14, partial | If_Uni_10103 | 1037 |
| Zonadhesin | If_Uni_00025, If_Uni_28016, If_Uni_33482, If_Uni_35531 | 832, 1274, 1366, 600 |
| LIM/homeobox protein Lhx2 | If_Uni_22589 | 1484 |
