# Supplementary material for: Transcriptome analysis of air-breathing land slug, Incilaria fruhstorferi reveals functional insights into growth, immunity, and reproduction
Source: BMC Genomics. 2019 Feb 26;20:154. doi: 10.1186/s12864-019-5526-3 (PMC6390351; doi:10.1186/s12864-019-5526-3)
Supplement: Supplementary file 14 — Table S4. Genes of interest related to growth in the land slug, Incilaria fruhstorferi. (DOCX 22 kb) [file 12864_2019_5526_MOESM14_ESM.docx]

**Additional file 14: Table S4**: Genes of interest related to growth in the land slug, *Incilaria fruhstorferi*

| Candidate genes  **Additional file 14: Table S4**: Genes of interest related to growth in the land slug, *Incilaria fruhstorferi* | Unigenes ID | Length (bp) |
| --- | --- | --- |
| Somatotrophic axis | | |
| Molluscan insulin-related peptide 3-like | If_Uni_45991 | 891 |
| Insulin-related peptide VII | If_Uni_44239, If_Uni_35150 | 1050, 731 |
| Epidermal growth factor receptor | If_Uni_05768, If_Uni_05769, If_Uni_05770, If_Uni_05771, If_Uni_24315, If_Uni_24316, If_Uni_24317, If_Uni_24318, If_Uni_24319, If_Uni_28915, If_Uni_28916, If_Uni_29221, If_Uni_31233, If_Uni_42437, If_Uni_43607 | 2359, 4886, 2277, 4968, 5392, 5227, 5309, 5286, 5344, 732, 1560, 1774, 2097, 998, 513 |
| Insulin receptor substrate 1 | If_Uni_44383 | 1014 |
| MIP-related peptides-like | If_Uni_41955 | 688 |
| Receptor for molluscan insulin-related peptide | If_Uni_36370, If_Uni_39403, If_Uni_42554, If_Uni_43241 | 1030, 274, 446, 338 |
| Mollusk-derived growth factor | If_Uni_15809, If_Uni_17091, If_Uni_26625, If_Uni_26626, If_Uni_32954 | 1461, 1901, 1294, 1940, 2041 |
| Tsetse EP protein | If_Uni_01454 | 299 |
| Cysteine-rich protein 1-like | If_Uni_07858, If_Uni_41055 | 1523, 2012 |
| Adenosine deaminase AGSA-like | If_Uni_00232, If_Uni_07458, If_Uni_45179 | 955, 1769, 1069 |
| Adenosine deaminase domain-containing protein 1-like | If_Uni_25413 | 2861 |
| POU domain, class 2, transcription factor 1 | If_Uni_33649, If_Uni_37270 | 2662, 2639 |
| POU domain, class 3, transcription factor 2 | If_Uni_43568 | 528 |
| POU domain, class 6, transcription factor 1 | If_Uni_15497, If_Uni_15498 | 1565, 1273 |
| Transcription factor Sox-2-like | If_Uni_29379 | 1869 |
| PAX3- and PAX7-binding protein 1-like | If_Uni_08833, If_Uni_10236, If_Uni_10237, If_Uni_39294, If_Uni_40823 | 1943, 2239, 2993, 2174, 1431 |
| PAX-interacting protein 1-like | If_Uni_44063 | 342 |
|  |  |  |
| Muscle growth | | |
| Actin | If_Uni_24222, If_Uni_24223, If_Uni_24226, If_Uni_24227, If_Uni_24228, If_Uni_24229, If_Uni_32868 | 628, 652, 514, 617, 908, 1442, 804 |
| Actin-1 | If_Uni_48246 | 369 |
| Actin-2-like | If_Uni_46459 | 897 |
| Actin-5C-like | If_Uni_12604 | 2547 |
| Actin, cytoplasmic | If_Uni_24221 | 1911 |
| Actin, adductor muscle-like | If_Uni_24230, If_Uni_29266, If_Uni_29267, If_Uni_34023 | 1497, 1995, 1672, 977 |
| Actin-like protein 6B | If_Uni_49673 | 1465 |
| Actin-interacting protein 1-like | If_Uni_38235, If_Uni_42654 | 1501, 964 |
| Actin-related protein 2-like | If_Uni_28972 | 3332 |
| Actin-related protein 2/3 complex | If_Uni_13597, If_Uni_22595, If_Uni_27842, If_Uni_45153, If_Uni_47955, If_Uni_50094 | 3942, 1454, 2811, 442, 1387, 1320 |
| Actin-related protein 3-like | If_Uni_09014 | 3112 |
| Actin-related protein 5-like | If_Uni_13117 | 2963 |
| Actin-related protein 6-like | If_Uni_18221, If_Uni_18222 | 1523, 2758 |
| Actin-related protein 8-like | If_Uni_03651, If_Uni_03826 | 2069, 2148 |
| Actin-binding protein IPP-like | If_Uni_29850, If_Uni_29851 | 2664, 1047 |
| Actin-binding protein anillin-like | If_Uni_30995, If_Uni_43520, If_Uni_43782 | 2388, 425, 327 |
| Actin-binding protein F-like | If_Uni_32065, If_Uni_38364 | 931, 767 |
| Actin-binding Rho-activating protein-like | If_Uni_42798 | 271 |
| Profilin-like | If_Uni_15850, If_Uni_25110, If_Uni_25111, If_Uni_25112 | 1005, 4456, 4449, 4513 |
| Profilin-4-like | If_Uni_15851, If_Uni_18729, If_Uni_47107, If_Uni_47167 | 1225, 1470, 870, 936 |
| Tropomyosin | If_Uni_06633, If_Uni_29387, If_Uni_37391, If_Uni_33585, If_Uni_33586 | 2784, 2920, 2741, 2883, 2836 |
| Tropomyosin-1 | If_Uni_06753, If_Uni_06754 | 2784, 2724 |
| Tropomyosin-2 | If_Uni_34989, If_Uni_36217, If_Uni_41608, If_Uni_45531 | 2468, 882, 3002, 522 |
| Growth/differentiation factor 8-like | If_Uni_48358 | 765 |
| Miscellaneous | | |
| Chitinase-like protein | If_Uni_00986, If_Uni_01277, If_Uni_11422, If_Uni_03370, If_Uni_13899, If_Uni_14759, If_Uni_17041, If_Uni_43048, If_Uni_17756, If_Uni_23341, If_Uni_24979, If_Uni_26112, If_Uni_26113, If_Uni_26114, If_Uni_26948, If_Uni_29418, If_Uni_33736, If_Uni_33737, If_Uni_43587, If_Uni_48457, If_Uni_49703, If_Uni_25075, If_Uni_10101, If_Uni_10102 | 2006, 1386, 3944, 1870, 2868, 2458, 2966, 2346, 1533, 1021, 2145, 1700, 1736, 1879, 3559, 1827, 2368, 2328, 279, 1779, 680, 3732, 524, 442 |
| Chitinase 3 | If_Uni_03027, If_Uni_05746, If_Uni_06062, If_Uni_08588, If_Uni_08589, If_Uni_15144, If_Uni_16700, If_Uni_16701, If_Uni_16702, If_Uni_16703, If_Uni_16704, If_16705, If_Uni_17502, If_Uni_18667, If_Uni_18668, If_Uni_18669, If_Uni_24166, If_Uni_24167, If_Uni_24170, If_Uni_24836, If_Uni_24837, If_Uni_24978, If_Uni_25012, If_Uni_25013, If_Uni_25014, If_Uni_25015, If_Uni_25016, If_Uni_25017, If_Uni_25854, If_Uni_25855, If_Uni_28758, If_Uni_37541, If_Uni_47731 | 2077, 3237, 390, 3237, 3024 2160, 2120, 1891, 1884, 2433, 2265, 1998, 602, 2981, 2768, 2371, 3636, 6795, 3021, 2353, 2293, 1184, 2962, 2792, 2946, 2777, 3002, 3071, 1014, 3438, 1394 |
| Endochitinase A | If_Uni_17663, If_Uni_37443 | 262, 2037 |
| Endochitinase-like | If_Uni_23342, If_Uni_23343, If_Uni_41456 | 1731, 2476, 937 |
| Chitinase domain-containing protein 1 | If_Uni_35132 | 1519 |
| Collagen-like protein | If_Uni_30984 | 1934 |
| Collagen alpha-1(I) chain-like | If_Uni_08744, If_Uni_08745, If_Uni_09485, If_Uni_09486, If_Uni_15928, If_Uni_27766, If_Uni_27767, If_Uni_29582, If_Uni_34918, If_Uni_37761, If_Uni_39405, If_Uni_39724 | 2643, 3984, 2558, 1269, 9047, 6483, 6691, 1652, 1063, 2081, 434, 2112 |
| Collagen alpha-1(II) chain-like | If_Uni_38954 | 320 |
| Collagen alpha-1(III) chain | If_Uni_17302, If_Uni_49593 | 503, 616 |
| Collagen alpha-1(IV) chain-like | If_Uni_18445 | 362 |
| Collagen alpha-1(XI) chain-like | If_Uni_28298 | 5791 |
| Collagen alpha-1(XII) chain-like | If_Uni_00436, If_Uni_01421, If_Uni_03271, If_Uni_25438, If_Uni_29701, If_Uni_29702, If_Uni_29703 | 1843, 1954, 1383, 4619, 2651, 2813, 2770 |
| Collagen alpha-1(XXII) chain-like | If_Uni_11799 | 1887 |
| Collagen alpha-2(IV) chain-like | If_Uni_05897, If_Uni_05898, If_Uni_24257, If_Uni_25608, If_Uni_25609, If_Uni_25610, If_Uni_25974, If_Uni_25975, If_Uni_27293, If_Uni_27561, If_Uni_28773, If_Uni_35362, If_Uni_36274 | 483, 1446, 1674, 5854, 2545, 4803, 2451, 6754, 714, 3428, 582, 743, 893 |
| Collagen alpha-2(XI) chain-like | If_Uni_25863, If_Uni_31669, If_Uni_37872, If_Uni_43365 | 3753, 2111, 1293, 447 |
| Collagen alpha-3(VI) chain-like | If_Uni_33369, If_Uni_37424, If_Uni_39048, If_Uni_49003 | 1846, 1813, 1756, 743 |
| Collagen alpha-4(VI) chain-like | If_Uni_40233 | 909 |
| Collagen alpha-5(VI) chain-like | If_Uni_19114, If_Uni_24479, If_Uni_27560, If_Uni_28004, If_Uni_35075, If_Uni_43843, If_Uni_48071 | 6006, 1243, 6579, 2780, 1178, 645, 519 |
| Collagen alpha-6(VI) chain-like | If_Uni_08184, If_Uni_09709, If_Uni_10293, If_Uni_15145, If_Uni_18925, If_Uni_18926, If_Uni_18927, If_Uni_26582, If_Uni_29141 | 2310, 2799, 2708, 3198, 3066, 940, 1854, 2834, 2671 |
| Collagen type IV alpha-3-binding protein-like | If_Uni_35586 | 611 |
| Yolk ferritin | If_Uni_22451, If_Uni_49605 | 2025, 1275 |
| Soma ferritin | If_Uni_23553, If_Uni_30675 | 1771, 1376 |
| Metallothionein-like | If_Uni_31828 | 310 |
| Metallothionein | If_Uni_49751 | 414 |
| Metallothionein isoform 20-1 | If_Uni_14651, If_Uni_45145, If_Uni_49717 | 2318, 1109, 1333 |
| Apolipophorins | If_Uni_16384, If_Uni_16385, If_Uni_42973 | 10902, 9355, 797 |
| Apolipoprotein B-100 | If_Uni_32444, If_Uni_39217 | 1138, 860 |
| Dermatopontin 2 | If_Uni_30657 | 856 |
| Dermatopontin 3 | If_Uni_32552, If_Uni_34403 | 1484, 653 |
| DNA topoisomerase 2-binding protein 1-A-like | If_Uni_24323, If_Uni_24324 | 5603, 3142 |
| DNA topoisomerase 2-alpha-like | If_Uni_31112 | 6022 |
| Perlucin-like protein | If_Uni_10721, If_Uni_10722, If_Uni_33177, If_Uni_34977, If_Uni_46081, If_Uni_46293, If_Uni_46336, If_Uni_46438, If_Uni_46522, If_Uni_49189, If_Uni_46138 | 2247, 2299, 824, 686, 951, 803, 731, 709, 703, 1189, 921 |
| Perlucin 5 | If_Uni_30825, If_Uni_31464, If_Uni_46117 | 883, 973, 773 |
| Calcitonin gene-related peptide type 1 receptor-like | If_Uni_45134, If_Uni_48682 | 723, 369 |
| Calcitonin receptor-like | If_Uni_49416 | 1141 |
